# State of shock – a systematic review of extracorporeal shockwave therapy in hand surgery

**DOI:** 10.3205/iprs000192

**Published:** 2025-12-22

**Authors:** Ibrahim Al-Mousllie, Peter M. Vogt, Andreas Jokuszies

**Affiliations:** 1 Department of Plastic, Hand and Reconstructive Surgery, Hannover Medical School, Hannover, Germany

**Keywords:** extracorporeal shockwave therapy, hand surgery, carpal tunnel syndrome, DeQuervain, trigger finger, osteonecrosis of the lunate, Kienböck

## Abstract

**Objective:**

As the number of elective hand surgeries has increased across the board, this systematic review aims to elucidate the effectiveness of extracorporeal shockwave therapy (ESWT) in hand surgery. Of interest are Dupuytren’s disease (DD), trigger finger (TF), De Quervain’s tenosynovitis, osteonecrosis of the lunate (ONL), and carpal tunnel syndrome (CTS).

**Methods:**

Qualitative analysis of the current evidence according to the Cochrane Handbook for systematic reviews of interventions and Preferred Reporting Items for Systematic Reviews and Meta-Analyses (PRISMA) statement using electronic databases, and quality assessment of the included studies using the Cochrane risk of bias in non-randomized Studies – of Interventions assessment tool, the Cochrane risk-of-bias tool for randomized trials and the measurement tool to assess systematic reviews 2 were performed.

**Results:**

ESWT with ≥5 sessions in DD improved functional status and symptom severity significantly and consistently, especially pain, in the short- and mid-term. In TF ESWT alleviated pain in the short-, mid- and long-term. Also, functional status and severity of triggering improved consistently in the mid- and long-term. Three sessions of ESWT are the optimal number in TF patients. In DQT ESWT improved pain and functional status in the short- and mid-term. One study showed LCI and ESWT to be equally effective in DQT patients. Another study showed ESWT as an effective treatment of the pain and progression of ONL. ESWT improved pain and functional status in CTS in the short- and mid-term, especially if ≥4 sessions of ESWT are performed. The long-term effectiveness of ESWT has been suggested but not sufficiently proven.

**Conclusions:**

ESWT is an effective and recommended treatment in DD, TF, and CTS to improve pain and functional status, especially, rESWT regarding CTS and likely also TF. It represents an equally effective option as local corticosteroid injections in TF, DD and CTS with fewer and less severe adverse effects. In the treatment of DQT, ESWT should be considered an option in the clinical setting. Further research is necessary to develop normed protocols and expand its scope of application.

**Trial registration:**

The review and search protocol were registered at PROSPERO (National Institute for Health and Care Research) – CRD42024598627.

## Zusammenfassung

**Zielsetzung:**

Da die Zahl der elektiven Handoperationen insgesamt gestiegen ist, zielt dieses systematische Review darauf ab, die Wirksamkeit der extrakorporalen Stoßwellentherapie (ESWT) in der Handchirurgie zu untersuchen. Dabei werden insbesondere Morbus Dupuytren (DD), Tendovaginitis stenosans (TF), die De Quervain Tendovaginitis (DQT), die Osteonekrose des Os lunatum (ONL), sowie das Karpaltunnelsyndrom (CTS) näher betrachtet.

**Methoden:**

Eine qualitative Analyse der aktuellen Literatur wurde gemäß dem Cochrane Handbuch für systematische Reviews von Interventionen („Cochrane Handbook for systematic reviews of interventions“) und dem Protokoll für bevorzugte Berichtsgegenstände für systematische Reviews und Metaanalysen („Preferred Reporting Items for Systematic Reviews and Meta-Analyses“) (PRISMA) unter Verwendung elektronischer Datenbanken durchgeführt. Zudem erfolgte eine qualitative Evaluation der eingeschlossenen Studien mithilfe des Cochrane Bewertungsinstruments des Risikos eines Bias bei nicht randomisierten Studien („Cochrane risk of bias in non randomized Studies - of interventions“) und des Risikos eines Bias bei randomisierten Studien („Cochrane risk of bias for randomized Studies of interventions“), sowie des Instruments zur Evaluation zur von systematischen Reviews („measurement tool to assess systematic reviews 2“) (AMSTAR-2).

**Ergebnisse:**

ESWT mit ≥5 Sitzungen bei DD verbesserte den funktionellen Status und die Schwere der Symptome, insbesondere den Schmerz, signifikant und konsequent sowohl kurz- als auch mittel- und langfristig. Bei TF wirkt ESWT kurz-, mittel- und langfristig schmerzlindernd. Zusätzlich verbesserte sich mittel- und langfristig konsequent der funktionelle Status und die Schwere des Fingerschnappens. Drei Sitzungen der ESWT ist die optimale Anzahl bei TF-Patienten. Bei DQT verbesserte ESWT kurz- und mittelfristig sowohl den Schmerz als auch den funktionellen Status. Eine Studie zeigte, dass LCI und ESWT bei DQT-Patienten gleichermaßen wirksam sind. Eine andere Studie ergab, dass ESWT eine effektive Therapie zur Schmerzlinderung und Verhinderung des Fortschreitens der ONL darstellt. ESWT verbesserte kurz- und mittelfristig den Schmerz und funktionellen Status bei CTS, insbesondere wenn ≥4 Sitzungen durchgeführt werden. Die langfristige Wirksamkeit von ESWT ist angedeutet, jedoch nicht ausreichend belegt.

**Schlussfolgerungen:**

ESWT ist eine effektive und zu empfehlende Therapieoption bei DD, TF und CTS zur Schmerzlinderung und Verbesserung des funktionellen Status, besonders radiale ESWT bei CTS und wahrscheinlich auch bei TF. Es stellt bei TF, DD und CTS eine ebenso wirksame Option wie lokale kortikosteroidhaltige Injektionen dar und hat weniger und schwächer ausgeprägte Nebenwirkungen. In der Behandlung der DQT sollte ESWT als Option in der klinischen Praxis in Betracht gezogen werden. Zur Entwicklung normierter Protokolle und Erweiterung des Anwendungsspektrums sind weitere Forschungsarbeiten erforderlich.

**Registrierung der Studie:**

Das Review- und Suchprotokoll wurde bei PROSPERO (National Institute for Health and Care Research) unter der Registrierungsnummer CRD42024598627 eingereicht.

## Background

This systematic review (SR) aims to answer two questions. First, if extracorporeal shockwave therapy (ESWT) is a viable and effective treatment option and second, if it could prevent a surgical intervention altogether. To do so, the current evidence of ESWT in the treatment of various hand conditions is analyzed and compared to already established treatment options providing a comprehensive overview of application possibilities and protocols. The hand conditions of interest in this review are Dupuytren’s disease, trigger finger, De Quervain’s disease, osteonecrosis of the lunate and carpal tunnel syndrome. 

As the number of elective hand surgeries has increased by 34% from 1990 to 2000 in the UK and by 16% in the US from 2011 to 2018 [1], [2] another effective nonsurgical treatment option would have great clinical impact. In the UK, the number of carpal tunnel syndrome surgeries increased by 88%, De Quervain and tenosynovitis surgeries by 41%, Dupuytren’s disease by 1.5% and trigger finger and thumb by 16% [2]. In the US, the Accreditation Council for Graduate Medical Education (ACGME) reported an increase of the number of surgeries for Dupuytren’s contracture by 28%, nerve decompressions by 26% and tendon conditions by 26% [1] from 2011 to 2018. Approximately 45–60% of all elective hand surgeries in the UK and US are for nerve decompression, De Quervain tenosysnovitis, Dupuytren’s contractures and trigger fingers [1], [2]. ESWT is a promising avenue in the treatment of hand conditions.

Extracorporeal shockwave therapy uses shockwaves emitted from a handpiece by accelerating a projectile that hits a metal applicator using compressed air to penetrate the respective tissue. Radial and focused are differentiated as two types of ESWT. Radial ESWT (rESWT) consists of low energy shock waves covering a larger volume of tissue while focused ESWT (fESWT) consists of high energy shock waves as they are directed onto a smaller surface area [3], [4], [5], [6]. The wave’s energy decreases by the square of the distance. Therefore, the highest pressure is exerted at the skin surface and the deeper the structure, the lower the shock wave’s energy. The energy flux density (EFD), frequency, focal volume, and penetration depth are adjustable [3], [7], [8], [9], [10], [11]. ESWT does not depend on imaging such as fluoroscopy or sonography and is a noninvasive procedure with no complications and side effects besides potentially some acute pain during and temporary reddening after a session [7], [12], [13]. Any ESWT treatment protocol must be developed and tested carefully. On the one hand, Lee et al. reported that the higher the EFD, the better was the therapeutic effect, which translated to less sessions but potentially more pain during the ESWT. The lower the pressure, the more sessions were necessary for the same therapeutic antinociceptive effect in plantar fasciitis [14]. On the other hand, Chen et al. have reported that higher EFD than 0.28 mJ/mm² damaged the Achilles tendon and paratenon in a rabbit model [15]. This suggests a ceiling effect of ESWT [16]. Another factor is the pain during a session. The higher the EFD, the stronger was the pain but if local anesthesia was used to numb the area of application it would hamper the positive effects of ESWT [17], [18], [19].

*Dupuytren’s disease* (DD) is one of the most common and debilitating diseases of the hand [20]. It is categorized as a superficial fibromatosis as is Ledderhose’s and Peyronie’s disease. ESWT has been shown to reduce pain in both, Ledderhose’s disease [21] and Peyronie’s disease [22], [23], [24]. That suggests a similar effect of ESWT in the treatment of DD and warrants further investigation. The current evidence on ESWT includes only a few comparative studies and only one SR of moderate quality. Therefore, the evidence has not been properly reviewed and analyzed. The assumed mechanism of action describes an increase of angiogenetic growth factors after ESWT stimulating neovascularization, along with cell proliferation and the expression of critical genes supporting the healing process of the tendon [25], [26], [27], [28]. The modulation of pain is hypothesized to be achieved via substance P or calcitonin gene related peptide (CGRP) [29].

*Trigger finger* (TF), also known as stenosing tendovaginitis, is treated by splinting, physical therapy, local corticosteroid injections, and a surgical release of the A1 pulley [30], [31], [32]. Surgical intervention is the treatment option for severe cases that exhausted conservative measures [33]. Surgery is associated with a longer recovery time and different possible complications such as tendon bowstringing, digital ulnar drift, nerve injuries [32] and painful scarring [34], [35]. The best treatment is still being discussed [16], [36], [37]. The current evidence on ESWT in TF includes only a few comparative studies but no SR to review and summarize the data on ESWT in TF. The hypothesized mechanism of action of ESWT in the treatment of TF is that the mechanical stimulus of the ESWT supports remodeling of the tissue by promoting inflammatory and catabolic processes that lead to the removal of damaged matrix constituents [15], [28], [38], [39], [40], [41], [42], [43], [44], [45], [46], [47], [48]. The modulation of pain is hypothesized to be achieved via substance P or CGRP [29], [49].

*De Quervain’s tenosynovitis* (DQT) is treated depending on its progression. In the final stage a surgical intervention is necessary [50], [51], [52], [53], [54], [55], [56], [57], [58], [59]. The current evidence on ESWT in DQT is very slim and no SR has been conducted until now. The assumed mechanism of action of ESWT is the same as described for DD.

*Osteonecrosis of the lunate* (ONL), also known as avascular necrosis or Kienböck’s disease, is mainly treated with splinting, analgesic or anti-inflammatory medication and physiotherapy which does not necessarily improve blood supply while surgical intervention is considered the last resort [60], [61]. The current evidence on ESWT in ONL, similar to DQT, is very slim and no SR has been conducted until now. This SR aims to contextualize the limited evidence by comparing in with other hand conditions, providing a basis for more concrete conclusions and directions for future research. ESWT has been shown to significantly reduce pain and the progression of osteonecrosis of the femoral head, a similar disease to ONL [62]. The hypothesis is that ESWT supports osteogenesis by stimulation of angiogenesis, release of growth factors and subsequent callus formation [63]. The modulation of pain is hypothesized to be achieved via substance P or CGRP [29], [49].

*Carpal tunnel syndrome* (CTS) is treated by splinting, physical therapy, local corticosteroid injections, or a release or endoscopic surgery. The current evidence on ESWT in CTS includes various RCTs and several SRs which are of different quality but did not make any conclusions regarding the specific protocol of ESWT. It is hypothesized that ESWT desensitizes the area of exposure; stimulating the expression of activating transcription factor 3 (ATF3) and growth associated phosphoprotein (GAP-43) which are markers for nerve injury and axonal regeneration [64]. The modulation of pain is hypothesized to be achieved via substance P or CGRP, reducing the number of neurons immunoreactive for substance P [29], [49].

The general consensus in the literature is that ESWT is effective in pain management and regeneration in bone diseases such as osteonecrosis of the femoral head [62], lunate bone [60] and nonunions [36] and tendinopathies such as epicondylitis [46], plantar fasciitis [4], [19] patellar tendinopathy [3], [65], shoulder calcific tendinitis [5], [7], [66], proximal hamstring tendinopathy [67], and medial tibial stress syndrome [8], [68]. As the role of ESWT in the treatment of patients grows, this study sheds some light on ESWT as a tool in the hand surgeon’s toolbox, new research angles and questions.

## Methods

This systematic review was performed in accordance with the Cochrane Handbook for systematic reviews of interventions [69] and the Preferred Reporting Items for Systematic Reviews and Meta-Analyses (PRISMA) statement [70] without meta-analysis.

### Search strategy

A systematic search of the electronic medical databases Web of Science Core Collection, ScienceDirect (Elsevier), PubMed, PubMed central, PubMed Clinical Queries (U.S. National Library of Medicine, National Institutes of Health), MEDLINE, CINAHL Ultimate, Cochrane Library, Google Scholar, Joanna Briggs Institute EBP Database (Ovid), Oxford Journals Current Content, BMC Musculoskeletal Disorders and Journal of Orthopaedic Surgery and Research (BioMed Central Ltd.) up to September 1^st^, 2024. Carefully constructed key words and search prompt using Boolean operators were employed. References of each included article were further researched to identify additional potential literature. Additionally, an expert on the subject matter was contacted. The search strategy was documented in detail in Attachment 1 using the template by the UNC Health Science Library [71].

### Inclusion and exclusion criteria

Amid the lack of literature for some conditions the inclusion criteria were broad in the interest of a complete representation of the current literature surrounding ESWT in the treatment of hand conditions. The following are the inclusion criteria:

1. A definitive diagnosis of CTS, trigger finger, osteonecrosis of the lunate, De Quervain’s and Dupuytren’s disease.
2. Any type of ESWT protocol.
3. Randomized controlled trials (RCTs).
4. SRs with meta-analyses including at least 3 RCTs.

Age, gender and nationality were not limited to. Observational studies are included in this review due to a lack of RCTs for most of the conditions in question.

Exclusion criteria were the following:

1. Trauma, fracture, tumor, infection, endocrine system disease etc. related pathogenesis of the condition of interest.
2. Existence of other hand conditions on the same hand.
3. Prior surgical intervention for the condition of interest.
4. Animal studies.
5. Articles not available in English or German.
6. Letters, comments, narrative, literature or umbrella reviews and case reports.

### Data extraction

One researcher conducted a literature search strictly following the inclusion and exclusion criteria. After identification, then screening, and detailed evaluation of a large number of studies the data of the included studies were extracted. The collected information includes name of the first author, year of publication, country, study design (determined according to Aggarwal and Ranganathan [72], [73]), study population, type and protocol of intervention (frequency, EFD, number of sessions), structure and composition of the control group, outcome measures, follow up intervals, and outcome. Any missing data is explicitly mentioned.

### Data synthesis

The data was categorized according to the condition of interest. Any missing data is explicitly mentioned and if the comprehension of the study is severely affected, the study was excluded if the request for more data from the authors was unsuccessful (Attachment 1). On account of the heterogeneity of the included studies regarding study design, treatment protocol and outcome measures a quantitative analysis or meta-analysis were not performed. Instead, qualitative analysis by determining the level of evidence and grade practice recommendations according to the American Society of Plastic Surgery (ASPS) were performed (Table S1 & S2 (Attachment 2)) [74], [75]. The main three outcomes of interest are symptom severity, especially pain, functional status of the involved joints and structures, and necessity of surgical intervention. To evaluate the three main outcomes, the outcome measures to quantify the effectiveness of ESWT used in the included studies were categorized as evaluating symptom severity, functional status and/or diagnostic parameters. That is necessary for a comprehensive overview of the effectiveness of ESWT as a multitude of different outcome measures were used in the included studies. Additionally, depending on the study design, reported comparisons with different treatment options are summarized. This approach aims to answer the two questions posed in the introduction.

The disabilities of the arm, shoulder, and hand (DASH) score [76], [77] evaluates symptom severity and functional status, as does the shorter version, the QuickDASH (qDASH) score [78]. The Michigan Hand Outcomes Questionnaire (MHQ) [79] and the Boston Carpal Tunnel Questionnaire (BCTQ) [80] evaluate symptom severity and functional status. The Mayo Wrist score [77] and the Roles and Maudsley score (R&Ms) [77], [81] primarily evaluates functional status and additionally pain. Unité Rhumatologique des Affections de la Main (URAM) evaluates symptom severity and functional status [82]. The 36-item Short Form Health Survey Questionnaire (SF-36) evaluates functional status and the overall health including pain [83]. The Activities of Daily Living (ADL) score evaluates functional status [84], [85]. In this SR, the Visual Analog Scale (VAS) [86], [87], [88] and the Numeric Rating Scale (NRS) [31], [89] were mostly used to evaluate symptom severity, especially pain, but can be used for functional status as well, and if so, is clearly marked. The Leeds Assessment of Neuropathic Symptoms and Signs (LANSS) evaluates symptom severity, more specifically pain [90]. The Ritchie’s tenderness scale evaluates symptom severity, more specifically tenderness [91]. The Trigger Finger Score by Quinnell [92], [93], the Froimson scale [31], [94] and the Lichtman classification [95] evaluate diagnostic parameters and functional status to some extent. The Trigger Finger Assessment Scale (TFA) including frequency (TFAfq), severity (TFAs) and functional impact (TFAfi) of triggering evaluates symptom severity [36], [96].

### Sensitivity and heterogeneity analysis

Sensitivity analyses were conducted by testing how different subgroups across multiple studies based on various criteria such as treatment groups and treatment protocol yield different results. This analysis identifies potential factors contributing to the variability and heterogeneity across the included studies.

### Risk of bias and quality assessment

The included studies were assessed for risk of bias (RoB) by one researcher dependent on their study design. To assess pre-post studies and case series the criteria of the Cochrane Risk of Bias in Nonrandomized Studies – of Interventions (ROBINS-I) assessment tool [97] was used and the criteria of the Cochrane risk of bias tool for randomized trials (RoB 2) [98] was employed to assess the RCTs. SRs were evaluated using the Measurement Tool to Assess systematic Reviews 2 (AMSTAR-2) [99]. The level of evidence for each condition and their treatment was determined using the evidence rating scale for therapeutic studies from the ASPS (Table S1 (Attachment 2 )). Any RCT with a moderate or worse RoB was a level of II assigned, only RCTs with a low RoB were considered level I. Pre-post studies (prospective comparative studies) were categorized as level II and case series as level IV.

## Results

**Figure 1:**
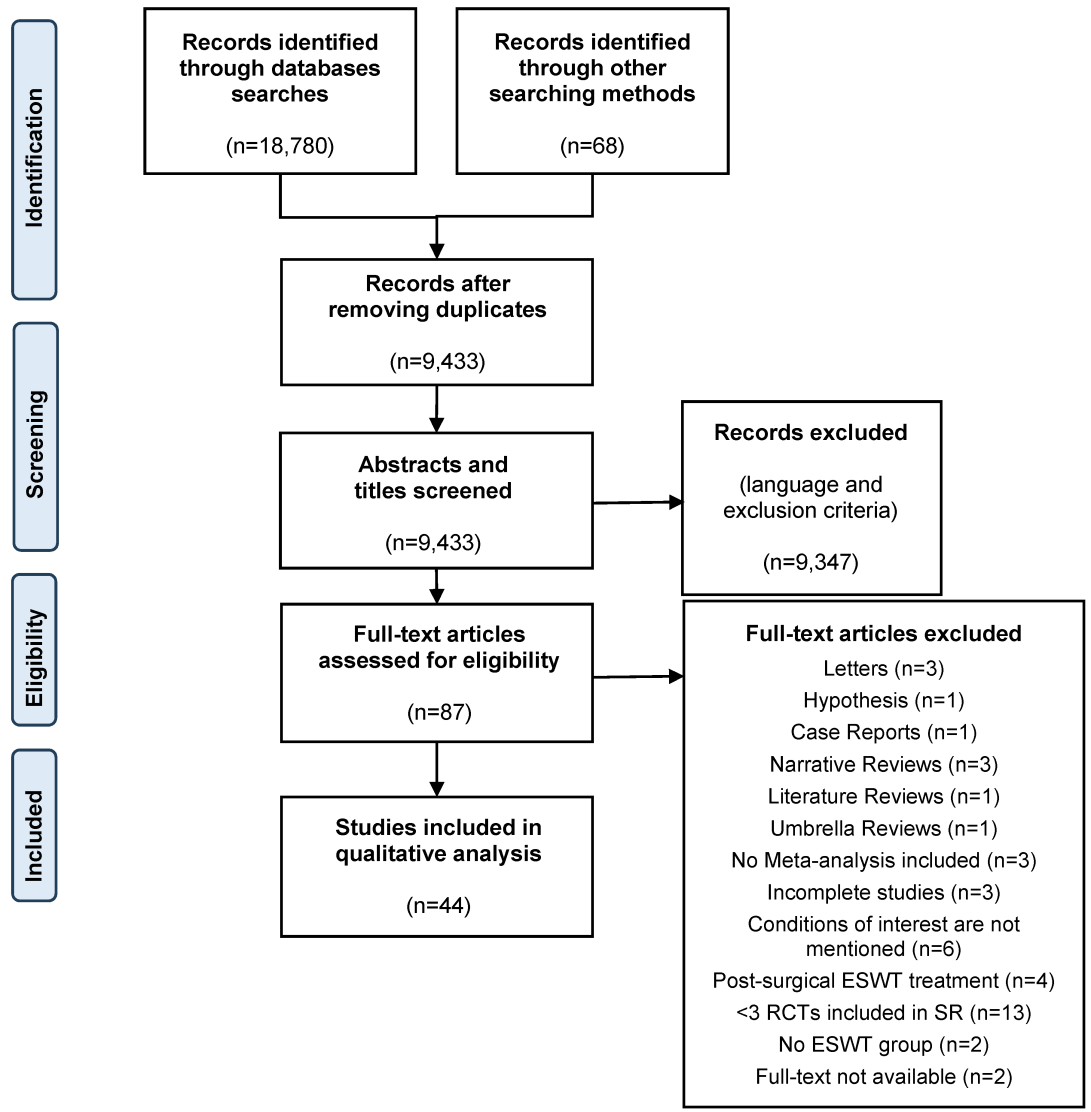
Flow chart

A total of 18,847 records were identified through database searches and other searching methods including contacting an expert, backwards and forwards reference searching. After removing duplicates, the abstract and title of 9,433 studies were screened according to exclusion criteria. Then, 87 full text articles were assessed for eligibility according to inclusion and exclusion criteria. A full list of excluded articles is presented in Attachment 1 . Finally, 44 studies were included in the qualitative analysis. The flow chart (Figure 1 (Fig. 1)) details the selection process according to the PRISMA guidelines for SRs.

### Study characteristics

Table S3 (Attachment 2) is an overview of the number of studies and their study designs for each condition discussed in this SR. The characteristics of each study are further detailed in Tables S4–S8 (Attachment 2). The included studies were published from 2011 to 2024 and conducted in 17 different countries. The total sample size of participants was over 16,623. The number of sessions depending on the treatment protocol ranged from a total of one to ten and the number of shocks from 500–5000. Depending on the treatment protocol the frequency ranged from 2 to 15 Hz and EFD from 0.006 to 1.24 mJ/mm². The heterogeneity is apparent in Tables S4–S8 (Attachment 2) regarding sample size, study design, treatment protocol, and outcome measures.

### Risk of bias and quality assessment

The assessment of the RoB of the included studies was illustrated in Tables S9–S11 (Attachment 2). RCTs were evaluated using the criteria of the Cochrane RoB 2 tool in Table S9 (Attachment 2). Pre-post studies and case series were evaluated using ROBINS-I tool in Table S10 (Attachment 2). SRs were evaluated using the AMSTAR-2 tool in Table S11 (Attachment 2). The level of evidence of each study according to the ASPS is recorded in Tables S12–S16 (Attachment 2) organized by condition.

### Outcomes

#### Dupuytren’s disease

Table S4 (Attachment 2) details the characteristics of the studies regarding DD and Table S12 (Attachment 2) illustrates the corresponding results. Three studies showed a significant improvement of pain and tenderness in the ESWT treated patients as early as six weeks and persisting up to 18 months posttreatment measured by the VAS score, MHQ pain domain or the MHQ tenderness domain [100], [101], [102]. One study was sham controlled and showed a significant increase of pain in the control group at three to 18 months compared to pretreatment baseline values and was significantly worse than the pain levels of the ESWT treated patients [100]. The functional status and symptom severity measured by the DASH or qDASH score recorded by three studies showed continuous and significant improvement in two of the three studies from six to 14 weeks posttreatment [100], [102], [103]. The MCP contraction angle recorded by only one study improved significantly and continuously from six to 14 weeks posttreatment [102]. The handgrip strength measured by three studies [100], [101], [103] significantly improve in only one [103] study and the URAM score did not change significantly [100]. One study showed in a comparison of ESWT to LIPUS (Low intensity ultrasound) that both significantly improved functional status and symptom severity, but ESWT was superior [103].

Fernando et al. evaluated eight studies (two case reports and two other studies that were assessed and excluded from this SR (Attachment 1)) as part of a SR and reported inconsistent pain reduction following ESWT. Only four of eight studies showed maintained reduction of pain measured by the VAS score. All four studies measuring ROM showed significant improvement but only one of the four studies showed improved handgrip strength. Additionally, R&Ms and MHQ were measured each in one study and improved significantly [104].

The three of four studies that showed significant improvement of symptom severity and functional status performed five sessions in one [101] and six sessions in two studies [102], [103], one per week. The fourth study performed three sessions over a span of three weeks and showed significant improvement of only pain [100]. No further sensitivity analysis was performed due to the small number of studies including regarding frequency and pressure of the ESWT. No adverse effects besides slight pain one to two days after sessions in one [102] study were reported.

Three [100], [102], [103] of the studies have a level of evidence of II and the fourth [101] of IV. The evidence regarding pain relief in the short and midterm is classified as a “B” on the grading scale from the ASPS, while the effectiveness in improving functional status applying ESWT at least in 5 sessions is classified as a “B”.

#### Trigger finger

Table S5 in Attachment 2 details the characteristics of the studies regarding TF and Table S13 in Attachment 2 illustrates the corresponding results. All six studies showed a significant improvement in pain in the ESWT treated patients measured on the VAS, NRS or R&Ms [12], [16], [31], [36], [105], [106]. The pain relief was recorded at different time points, one [105] study showed improvement of pain immediately after the last session and another [12], showed that the improvement persisted up to 12 months posttreatment. Three of four studies assessing the severity of triggering using the VAS, TFA or grading scale by Froimson showed significant improvement at different time points, the earliest was one month posttreatment [31], [36], [105]. Functional status was measured by using the qDASH score recorded by three studies [16], [36], [106], ROM, handgrip and pinch strength recorded by only one [106] study and the R&Ms and VAS each recorded by another study [12], [105]. Quantified by these measures functional status improved significantly measured over different time points compared to pretreatment baseline values and persisted 6–12 months posttreatment. One study showed that ESWT treatment is significantly more effective than the sham ESWT treatment [16], while another study showed no significant difference between ESWT and LCI treatment regarding pain relief and improvement of functional status [36]. No clear trend favoring rESWT or fESWT was apparent in the included studies. The protocol of ESWT in one study consisted of one session and showed significant improvement of pain [12], while three studies performing three sessions, one per week, showed significant and continuous improvement in pain and functional status [31], [36], [105]. Two studies’ protocol of ESWT consisted of four sessions, one per week, and ten sessions, two per week and showed significant improvement of pain and functional status but the improvement came into effect later, only three and six months posttreatment, than in the other studies [16], [106]. No further sensitivity analysis was performed due to the small number of studies including regarding frequency and pressure of the ESWT. The level of evidence of all 6 studies [12], [16], [31], [36], [105], [106] was level II and overall is classified as a “B” on the grading scale from the ASPS regarding pain relief and improvement of functional status in the short, mid, and long term.

#### De Quervain’s tenosynovitits

Table S6 (Attachment 2) details the characteristics of the studies regarding DQT and Table S14 (Attachment 2) illustrates the corresponding results. Both studies showed a significant and one study continuous improvement of pain and tenderness in the ESWT treated patients immediately after the last session persisting up to six months posttreatment measured by the VAS score and on the Ritchie’s tenderness scale. The functional status and symptom severity of the upper limb measured by DASH, or the overall health measured by SF-36 improved significantly in both studies in the ESWT treated patients. These improvements of functional status and pain were significantly better than the observed changes in the sham group but did not differ significantly from the LCI group [50], [107]. The handgrip strength in the ESWT treated patients significantly improved only in one study [107]. No adverse effects were reported. No further sensitivity analysis was performed due to small number of studies. The level of evidence of both studies [50], [107] was level II and is classified as a “C” on the grading scale from the ASPS regarding pain relief and improvement of functional status in the short and midterm but due to the small number of studies and no long term follow-up data it is not an “A” or “B” on the grading scale.

#### Osteonecrosis of the lunate

The search resulted in only one study [60]. In that study D’Agostino et al. measured pain with the VAS score and ROM which both significantly improved two months posttreatment compared to the pretreatment baseline values and did not further change significantly six months and one year posttreatment. The greatest improvement was observed in Lichtman’s stage I patients. MR imaging showed a reduction of bone marrow edema posttreatment and no changes in the already necrotic areas of the bone. No adverse effects were reported in this study [60]. No further sensitivity analysis was performed due to small number of studies. The level of evidence is level II but as it is only one study reported on the effects of ESWT in ONL no clinical implications should be derived.

#### Carpal tunnel syndrome

Table S8 (Attachment 2) details the characteristics regarding CTS and Table S16 (Attachment 2) illustrates the corresponding results. Symptoms of CTS such as pain, paresthesia and numbness recorded in 23 studies using VAS, BCTQs, qDASH, LANSS, R&Ms or GSS improved significantly in all 23 studies [108], [109], [110], [111], [112], [113], [114], [115], [116], [117], [118], [119], [120], [121], [122], [123], [124], [125], [126], [127], [128], [129], [130]. This improvement occurred as early as immediately after the last session by one study [115] or one week posttreatment by five studies [112], [120], [121], [124] and lasted up to six months posttreatment, the end of the studies [116], [117], [127]. The functional status was examined in 21 studies [108], [109], [110], [111], [112], [113], [114], [115], [116], [117], [118], [119], [120], [121], [122], [123], [124], [126], [128], [129], [130] using BCTQf, qDASH, SF-36, R&Ms, pinch or handgrip strength improved significantly in 20 of them [108], [109], [110], [111], [112], [113], [115], [116], [117], [118], [119], [120], [121], [122], [123], [124], [126], [128], [129], [130]. At 6 months posttreatment, the end of the study one study reported that 75% of patients receiving sham ESWT combined with LCIs needed surgical intervention which was significantly higher than the 40% of patients treated with ESWT combined with LCIs needing surgical intervention [127]. ESWT was compared to sham ESWT in nine [109], [111], [112], [118], [119], [126], [127], [128], [129] studies demonstrating that ESWT is superior in improving pain in eight [109], [111], [112], [118], [126], [127], [128], [129] of the nine studies and functional status in seven [111], [112], [118], [126], [127], [128], [129] of the nine studies. ESWT was compared with LCI treatment in five studies [108], [110], [114], [117], [121]. Two studies [110], [117] demonstrate that ESWT is superior in improving pain and functional status while no difference was found in two other studies [108], [114]. One study showed that LCI treatment is superior to ESWT in both, improving pain and functional status [121]. Another study showed that pain relief was greater in the group receiving ESWT combined with LCI than the group receiving sham ESWT combined with LCI but no difference in the functional status between both groups [127]. Also, ESWT was compared with physiotherapy as treatment in four studies. In all four studies, pain relief and the improvement of functional status was greater in the ESWT treated patients than in the patients receiving only physiotherapy [120], [122], [123], [124]. One 2x2 factorial RCT investigated splinting and ESWT and found improvement in all 4 groups regarding pain, functional status and electrodiagnostic parameters but did not differ significantly from each other in any category [126]. Also, ESWT was compared with a nutraceutical treatment and LLLT showing no significant difference between those treatments and ESWT [116]. One study compared ESWT with US and Cryo-US showing that ESWT is superior in pain relief [115] and another study compared ESWT to US combined with physiotherapy showing that ESWT is superior in pain relief and improvement of functional status [122]. Multiple electrodiagnostic parameters were measured across 19 studies [108], [109], [110], [111], [112], [113], [114], [116], [117], [118], [119], [120], [121], [123], [124], [126], [128], [129], [130]. The SNAPA significantly improved in four of six studies [124], [126], [129], [130] and SNAPL in 3 of 3 studies [110], [113], [118]. The CMAPA significantly improved in only four of 11 studies [108], [121], [124], [126] and CMAPL in 3 of 3 studies [110], [113], [118]. The DSL significantly improved in six of seven [117, 120, 126, 128-130] and DML in eight of 12 studies [108], [109], [116], [119], [120], [124], [128], [129]. The MNCV improved significantly only in one of four studies [126] and SNCV in nine of 11 studies [109], [112], [116], [119], [121], [123], [124], [128], [130]. The CSA significantly improved in four of five studies [109], [111], [112], [125]. No serious adverse effects were reported.

Additionally, six SRs were included [131], [132], [133], [134], [135], [136] (Table S8 (Attachment 2)). Their results are presented in Table S16 (Attachment 2).

Three SRs compared ESWT treatment to sham ESWT treatment. All three SRs concluded that ESWT treatment is superior in improving symptom severity, especially pain, measured by the BCTQs and VAS score in the short and long term (<1 month to >6 months). Functional status measured by the BCTQf improved significantly in the short and long term (<1 month to >6 months). Electrodiagnostic parameters such as DML, CMAPL, SNCV, and CSA only improved inconsistently over the three SRs [132], [134], [136]. Two SRs compared ESWT treatment to splinting alone. Both concluded that ESWT treatment is superior in reducing symptom severity, especially pain, measured by the BCTQs and VAS score, and improving functional status measured by the BCTQf score (and qDASH) but only in the short term (up to 6 weeks). Only one SR reported a significant improvement of SNCV and the other of CMAPA but no significant improvement of DSL or DML [131], [135]. Three SRs compared ESWT treatment to LCI treatment. All three SRs concluded that there is no significant difference between the two treatments regarding symptom severity and functional status in the short term. Only one SR reported significantly better scores for symptom severity and functional status in the mid and long term (>1 month to >6 months). Two SRs measured electrodiagnostic parameters such as DML, SNAPA, CMAPA, and CMAPL which only changed inconsistently over the two SRs [132], [133], [136]. Three SRs conducted a subgroup analysis to compare rESWT to fESWT [132], [134], [136]. Two SRs concluded that rESWT is more effective than fESWT in improving the VAS, BCTQs, and BCTQf score in the mid to long term (>1 month to >6 months) [134], [136]. Kim et al. reported no significant difference between rESWT and fESWT [132].

Only five studies [110], [113], [117], [120], [124] and no SRs reported serious adverse effects. The reported side effects were slight pain during and temporarily after the session [110], [113], [117], [120], one study reported additionally temporary reddening [120], and another reported some swelling and numbness in a few patients [124].

The data examined in this SR shows that at least 4 or more sessions, one per week, are required to significantly and consistently improve symptom severity and functional status compared to sham ESWT and physiotherapy. One study [110] that entailed nine sessions, three per week, showed significant improvement of symptom severity and functional status compared to LCIs even though the other four studies [108], [114], [117], [121] comparing ESWT to LCI and performing one, two or three sessions did not show any superiority of ESWT. The larger sample size for CTS than in the other conditions allowed for an analysis regarding EFD and frequency but no clear correlation of these two variables to outcome could be shown.

These studies have different levels of evidence. Seven studies [109], [110], [112], [115], [120], [125], [129] of them are classified as level I, 15 studies [108], [111], [113], [114], [116], [117], [118], [119], [121], [122], [123], [124], [126], [127], [128] of them are level II and one [130] of them is level IV. The body of evidence for the effectiveness of ESWT to relief pain and improve functional status in the short and midterm is classified as a “A” on the grading scale from the ASPS. The evidence for long-term and lasting effects of ESWT regarding pain relief and improving functional status is not as consistent as the short to midterm effects and are therefore classified as “B” on the ASPS grading scale.

## Discussion

This SR is about whether ESWT is a viable and effective treatment option and if it can prevent a surgical intervention or is solely a complementary tool in the conservative pre-operative treatment.

The effectiveness of ESWT in *Dupuytren’s disease* in pain relief in the short and midterm has been shown. Functional status and symptom severity improved only inconsistently. One study showed that ESWT is superior to LIPUS, an established treatment for DD. A recommendation of ESWT in pain management can be given. Regarding the improvement of functional status, the data is inconsistent if all the studies are included. Only including studies performing five or more sessions shows significant and more consistent improvements. Therefore, the application of ESWT to improve functional status can be recommended as well. Further research validating these findings and monitoring long-term effects are necessary due to the small numbers of studies. Also, the treatment protocol should entail five to six sessions but regarding EFD and frequency further testing and more comparative studies testing for superiority or equivalence with other established treatment options like US, LCI or vitamin E treatment should be conducted.

The effectiveness of ESWT in *trigger finger* for pain relief in the short, mid and long term has been shown. Functional status and severity of triggering improved consistently in the mid and long term. One study showed that ESWT does not differ significantly from LCI. Many other conservative treatment options like activity restriction, stretching, splinting, NSAIDs, LCIs [137] are available but one is not consistently better than the other [16]. Another effective treatment option for TF could be clinically impactful as for example LCIs report a high success rate only achieved by multiple injections increasing the risk for side effects and a high recurrence rate of the condition. Even though LCIs are generally cheaper than ESWT, the requirement of multiple LCIs and the only short-term effects make LCI treatment often non cost effective [138], [139], [140], [141], [142]. The data suggests a recommendation for ESWT to alleviate pain and improve functional status. The optimal number of sessions suggested by this SR’s analysis is three sessions, one per week. A higher number of sessions produced a worse outcome. This ceiling effect was not observed in any other condition and should be investigated further. Nevertheless, further research specifying the treatment protocol even more and validate the correlation and observations made by this SR. Additionally, it is necessary to validate the equivalence of ESWT to LCI and explore potential superiority of ESWT when optimizing the treatment protocol as seen in one study concerning CTS, as well as the comparison to other treatment options is necessary.

The treatment of *De Quervain’s tenosynovitis* with ESWT seems to be effective in pain relief and improving functional status in the short and midterm. LCI treatment is considered the best nonsurgical treatment option [143], [144], [145], [146]. One study showed LCI and ESWT to be equally effective. ESWT presents a clinical option in the treatment of DQT. Due to the small number of studies no recommendations or conclusions could be made regarding the treatment protocol (number of sessions, EFD, frequency). No studies researching the long-term effects were found. Further research focusing on the long-term effects and specifying the protocol, as well as the comparison of ESWT and LCI is necessary before a definite clinical recommendation can be made. 

The clinical impact of this SR in the treatment of *osteonecrosis of the lunate* is limited due to a lack of data. One study showed ESWT as an effective treatment of the pain and progression of the disease. Further research should be done especially in patients with Lichtman stage I ONL. Also, ESWT was suggested as a palliative treatment for advanced stages of osteonecrosis as it could be effective in alleviating pain [147]. Due to the small number of studies no recommendations or conclusions could be made regarding the treatment protocol (number of sessions, EFD, frequency). Further research is needed to explore the effects and benefits of ESWT in ONL.

It has been shown in this SR that ESWT is effective in relieving pain and improving functional status in the short and midterm in *carpal tunnel syndrome*. The long-term effectiveness of ESWT should be researched further, but this SR identifies a strong tendency towards a long-term effectiveness of ESWT as well. Therefore, ESWT is recommended in the treatment of CTS, especially in the short and midterm. Additionally, the data inconsistently suggests that rESWT is superior to fESWT in the treatment of CTS. Also, splinting alone has been shown to be effective in pain relief. Therefore, a combination of splinting and ESWT treatment is to be considered in a clinical setting. The analysis revealed that at least 4 sessions, one per week, should be performed for consistent results. Importantly, multiple studies showed that ESWT and LCI significantly improve pain and functional status but do not differ significantly. One study performing nine sessions, three per week, showed ESWT to be superior to LCI. Increasing the frequency of sessions and the number of sessions should be researched further. LCI is an established and useful tool in the treatment of CTS but is associated with several side effects, like allergic reactions, dermal or subcutaneous atrophy, transient hyperglycemia, hypopigmentation of the skin, infection, rupture of the tendon, allergic reactions or even a fear of injections (“needle phobia”) [138], [148], [149], [150]. Therefore, ESWT is a great alternative as most times no side effects are reported and if so, they are minimal ranging from slight pain to transient swelling and numbness. Moreover, one study reported that ESWT treated patients were significantly less likely to need surgical intervention at the end of the treatment regimen than LCI treated patients.

To answer the question of whether ESWT can prevent surgical intervention, there is a lack of data. Only one of the 44 included studies documented if surgical intervention became necessary after the end of the study. That study evaluated the effects of ESWT in CTS and reported that patients receiving ESWT combined with LCI are less likely to need surgery than patients receiving sham ESWT combined with LCI [127]. More research is needed to further explore ESWT as a surgery preventing intervention.

The pain alleviating effect of ESWT [17], [43], [151], [152], [153] is consistent in all the conditions of interest and was expected along with the low incidence of adverse effects and complications as many other studies have reported [50], [154], [155], [156], [157], [158]. Unexpected were the effects of ESWT on the functional status and in part progression of the disease or condition. Regarding the treatment protocol, the number of sessions seem to be more consequential than the EFD or frequency of ESWT. Leaning on this SR’s conclusions and existing work a minimum of three sessions, no local anesthesia before the treatment [17], [18], [19], and a generally high EFD [14] provide the optimal framework for future research.

## Study limitations

To our knowledge this is the first SR evaluating the effectiveness of ESWT as a presurgical treatment to reduce the number of elective hand surgeries. A limitation of this SR is the lack of data regarding some of the conditions of interest. Several conditions have not been researched at all regarding ESWT and some conditions were only researched in a few studies. A relatively large number of studies is necessary to evaluate recurrence rate, long-term effects and if a surgical intervention was prevented entirely or only delayed. Another limitation due to the lack of data is the limited comparison of different treatment protocols for each condition as they have multiple adjustable variables. Therefore, making a definite recommendation for a particular protocol is impossible. Also, important to mention is the very small number of studies comparing ESWT with already established treatment options for the conditions of interest and therefore limiting further conclusions. Additionally, this SR does not include a meta-analysis. Moreover, the inclusion and exclusion criteria of this SR limited this SR’s selection of studies. Of course, a publication bias should be anticipated limiting this and any SR in their findings.

## Conclusion

ESWT is a viable, effective and recommended treatment in DD, TF, and CTS regarding pain relief and improvement of functional status, especially, rESWT in the case of CTS and likely also TF. It represents an equally effective option as LCIs in TF, DD and CTS with fewer and less severe adverse effects. In the treatment of DQT, ESWT should be considered an option in the clinical setting. Further research is needed to validate the suggested effectiveness of ESWT in the treatment of ONL. For all conditions three ESWT sessions is the minimum amount, and for some conditions even more sessions. A ceiling effect was observed only in the case of TF. Therefore, ESWT is a useful tool to manage and improve these conditions before surgical intervention. Additionally, the use of ESWT should be considered in some conditions even with a small body of evidence as only very few and minor side effects are to be expected. Weather ESWT can reduce the number of elective hand surgeries cannot be definitively concluded by this SR due to a lack of data. Further research is required to answer this question and to develop normed protocols and expand its scope of application. Hence, this SR emphasizes the necessity of a prospective, multi-centered RCT, especially so ESWT for these hand conditions can eventually be covered by public health insurance in Germany, as it already is for plantar fasciitis.

## Abbreviations

- ADL: Activities of Daily Living score
- BCTQ: Boston Carpal Tunnel Questionnaire
- CMAPL: Compound muscle action potential latency
- CSA: Cross-sectional area
- CTS: Carpal tunnel syndrome
- DASH: Disabilities of the arm, shoulder, and hand score
- DD: Dupuytren’s disease
- DML: Distal motor latency
- DQT: De Quervain’s tenosynovitis
- DSL: Distal sensory latency
- EFD: Energy flux density
- ESWT: Extracorporeal shockwave therapy
- fESWT: Focused ESWT
- LANSS: Leeds Assessment of Neuropathic Symptoms and Signs
- LCI: Local corticosteroid injection
- MHQ: Michigan Hand Outcomes Questionnaire
- NRS: Numeric Rating Scale
- ONL: Osteonecrosis of the lunate
- qDASH: QuickDASH score
- R&Ms: Roles and Maudsley score
- RCT: Randomized-controlled trial
- rESWT: radial ESWT
- SF-36: 36-item Short Form Health Survey Questionnaire
- SNCV: Sensory nerve conduction velocity
- SR: Systematic review
- TF: Trigger finger
- TFA: Trigger Finger Assessment Scale
- TFAfi: Functional impact of triggering on the TFA
- TFAfq: Frequency of triggering on the TFA
- TFAs: Severity of triggering on the TFA
- URAM: Unité Rhumatologique des Affections de la Main
- US: Ultrasound/sonography
- VAS: Visual analog scale

## Notes

### Trial registration

This review and search protocol were registered at PROSPERO (National Institute for Health and Care Research) – CRD42024598627.

### Availability of data and materials

All data and materials generated or analyzed during this study are included in Attachment 1 and Attachment 2.

### Competing interests

The authors declare that they have no competing interests.

## Supplementary Material

Search strategy documentation

Supplementary tables
