# Supplementary material for: State of shock – a systematic review of extracorporeal shockwave therapy in hand surgery
Source: GMS Interdiscip Plast Reconstr Surg DGPW. 2025 Dec 22;14:Doc03. doi: 10.3205/iprs000192 (PMC12833726; doi:10.3205/iprs000192)
Supplement: Search strategy documentation [file IPRS-14-03-s-001.pdf]

Search Strategy Documentation

Extracorporeal Shockwave Therapy In Treatment Of Hand Conditions

Contents[1]

Summary by Source 2

Database Searches 3

Registry/Website Searches 17

Forward and Backward Reference Searching 17

Subject Matter Experts Contacted 18

Additional Notes 18

Summary by Source

	Source	Date Searched	Number of Results
Database Searches Only	PubMed (U.S. National Library of Medicine, National Institutes of Health)	September 1, 2024	2,210
	Web of Science Core Collection (Web of Science)	September 1, 2024	1,622
	MEDLINE (U.S. National Library of Medicine, National Institutes of Health)	September 2, 2024	285
	CINAHL Ultimate (EBSCO)	September 2, 2024	660
	Cochrane Library (Cochrane Collaboration)	September 2, 2024	423
	Google Scholar (Google LLC)	September 2, 2024	2,206
	ScienceDirect (Elsevier)	September 3, 2024	3,080
	PubMed Clinical Queries (U.S. National Library of Medicine, National Institutes of Health)	September 3, 2024	671
	PubMed Central (U.S. National Library of Medicine, National Institutes of Health)	September 4, 2024	7,509
	Joanna Briggs Institute EBP Database (Ovid)	September 4, 2024	21
	Oxford Journals Current Content (Oxford University Press)	September 4, 2024	15
	BMC Musculoskeletal Disorders (BioMed Central Ltd)	September 4, 2024	48
	Journal of Orthopaedic Surgery and Research (BioMed Central Ltd)	September 4, 2024	30
	Total (Including Duplicates)		**18,780**
	Total (After Removing Duplicates)		**9,370**
Other Searching Methods	Forward and Backward Searches	September 4, 2024	12
	Experts Contacted	September 11, 2023	56
	Total (Including Duplicates)		**68**
	Total (After Removing Duplicates)		**63**
**All Searching Methods Combined**	**Total (Including Duplicates)**		**18,848**
	**Total (After Removing Duplicates)**		**9,433**

Database Searches

PubMed (U.S. National Library of Medicine, National Institutes of Health): September 1, 2024

Search	Query	Number of Results
1	("Extracorporeal Shockwave Therapy"[Mesh] OR extracorporeal shockwave therapy[tiab] OR shockwave therapy[tiab] OR ESWT[tiab] OR SWT[tiab] AND (hand condition[tiab] OR hand conditions[tiab] OR hand disease[tiab] OR hand disease[tiab] OR hand surgery[tiab] OR hand operation[tiab] OR hand[tiab]))	102
2	("Hand"[Mesh] AND ("Extracorporeal Shockwave Therapy"[Mesh] OR extracorporeal shockwave therapy[tiab] OR shock wave[tiab] OR ESWT[tiab] OR SWT[tiab]) AND (overview[tiab] OR review[tiab] OR conservative[tiab] OR conservative treatment[tiab] OR non-surgical[tiab]))	2
3	(("Extracorporeal Shockwave Therapy"[Mesh] OR "Hand"[Mesh] OR extracorporeal shockwave therapy[tiab] OR shock wave[tiab] OR ESWT[tiab] OR SWT[tiab]) AND (carpal tunnel syndrome[tiab] OR CTS[tiab] OR nerve compression syndrome[tiab] OR compression neuropathy[tiab] OR carpal canal syndrome[tiab] OR thenar of carpal origin[tiab] OR amyotrophy carpal origin[tiab] OR distal median nerve compression[tiab] OR distal median nerve entrapment[tiab] OR entrapment neuropathy[tiab] OR median neuropathy[tiab])) (only clinical trials, meta-analyses, random controlled trials, reviews, systematic reviews	222
4	(("Extracorporeal Shockwave Therapy"[Mesh] OR "Hand"[Mesh] OR extracorporeal shockwave therapy[tiab] OR shock wave[tiab] OR ESWT[tiab] OR SWT[tiab]) AND (Trigger finger[tiab] OR digital stenosing tenosynovitis[tiab] OR stenosing tenosynovitis[tiab] OR trigger thumb[tiab] OR tendovaginitis stenosans[tiab] OR digital tendovaginitis stenosans[tiab] OR digitus saltans[tiab] OR digital flexor tenosynovitis[tiab] OR tendovaginitis finger[tiab] OR tenosynovitis stenosans[tiab] OR stenosing tendovaginitis[tiab] OR tenosynovitis finger[tiab] OR trigger digit[tiab] OR locked finger[tiab]))	654
5	(("Extracorporeal Shockwave Therapy"[Mesh] OR "Hand"[Mesh] OR extracorporeal shockwave therapy[tiab] OR shock wave[tiab] OR ESWT[tiab] OR SWT[tiab]) AND (Dupuytren's Disease[tiab] OR Dupuytren Disease[tiab] OR Dupytren's contracture[tiab] OR Dupuytren contracture[tiab] OR Morbus Dupuytren[tiab] OR contraction of palmar fascia[tiab] OR familial palmar fibromatosis[tiab] OR palmar fascial fibromatosis[tiab] OR palmar fibromatosis[tiab] OR Viking's Disease[tiab] OR Viking Disease[tiab] OR Celtic hand[tiab]))	342
6	(("Extracorporeal Shockwave Therapy"[Mesh] OR "Hand"[Mesh] OR extracorporeal shockwave therapy[tiab] OR shock wave[tiab] OR ESWT[tiab] OR SWT[tiab]) AND (DeQuervain's Syndrome[tiab] OR DeQuervain's Disease[tiab] OR DeQuervain's tendinopathy[tiab] OR DeQuervain's tenosynovitis[tiab] OR DeQuervain Syndrome[tiab] OR DeQuervain Disease[tiab] OR DeQuervain tendinopathy[tiab] OR DeQuervain tenosynovitis[tiab] OR De Quervain Syndrome[tiab] OR De Quervain disease[tiab] OR De Quervain tendinopathy[tiab] OR De Quervain tenosynovitis[tiab] OR Black berry thumb[tiab] OR texting thumb[tiab] OR gamer's thumb[tiab] OR gamer thumb[tiab] OR radial styloid tenosynovitis[tiab] OR washerman's sprain[tiab] OR washerwoman sprain[tiab] OR mother's wrist[tiab] OR mother wrist[tiab] OR mommy thumb[tiab] OR designer's thumb[tiab] OR designer thumb[tiab] OR DeQuervain's tendinitis[tiab] OR DeQuervain tendinitis[tiab] OR De Quervain tendinitis[tiab]))	854
7	(("Extracorporeal Shockwave Therapy"[Mesh] OR "Hand"[Mesh] OR extracorporeal shockwave therapy[tiab] OR shock wave[tiab] OR ESWT[tiab] OR SWT[tiab]) AND (Kienböck's disease[tiab] OR Kienböck disease[tiab] OR Kienbock disease[tiab] OR Kienbock[tiab] OR Kienböck[tiab] OR osteonecrosis of lunate[tiab] OR osteonecrosis lunate bone[tiab] OR osteonecrosis carpal lunate[tiab] OR avascular necrosis lunate[tiab] OR aseptic necrosis lunate[tiab] OR lunatomalacia[tiab] OR osteochondrisis lunate[tiab] OR progressive avascular necrosis lunate[tiab] OR progressive aseptic necrosis lunate [tiab] OR progressive osteonecrosis lunate[tiab]))	34
	**Total**	**2,210**

Web of Science Core Collection (Web of Science): September 1, 2024

Search	Query	Number of Results
1	(AB=(extracorporeal shockwave therapy) OR AB=(shockwave therapy) OR AB=(ESWT) OR AB=(SWT) OR AB=(shock wave) OR TI=(extracorporeal shockwave therapy) OR TI=(shockwave therapy) OR TI=(ESWT) OR TI=(SWT) OR TI=(shock wave) OR TS=(extracorporeal shockwave therapy) OR TS=(shockwave therapy) OR TS=(ESWT) OR TS=(SWT) OR TS=(shock wave)) AND (AB=(hand condition) OR AB=(hand conditions) OR AB=(hand disease) OR AB=(hand disease) OR AB=(hand surgery) OR AB=(hand operation) OR AB=(hand) OR TI=(hand condition) OR TI=(hand conditions) OR TI=(hand disease) OR TI=(hand disease) OR TI=(hand surgery) OR TI=(hand operation) OR TI=(hand) OR TS=(hand condition) OR TS=(hand conditions) OR TS=(hand disease) OR TS=(hand disease) OR TS=(hand surgery) OR TS=(hand operation) OR TS=(hand))	1399
	(AB=(extracorporeal shockwave therapy) OR AB=(shockwave therapy) OR AB=(ESWT) OR AB=(SWT) OR AB=(shock wave) OR TI=(extracorporeal shockwave therapy) OR TI=(shockwave therapy) OR TI=(ESWT) OR TI=(SWT) OR TI=(shock wave) OR TS=(extracorporeal shockwave therapy) OR TS=(shockwave therapy) OR TS=(ESWT) OR TS=(SWT) OR TS=(shock wave)) AND (AB=(hand condition) OR AB=(hand conditions) OR AB=(hand disease) OR AB=(hand disease) OR AB=(hand surgery) OR AB=(hand operation) OR AB=(hand) OR TI=(hand condition) OR TI=(hand conditions) OR TI=(hand disease) OR TI=(hand disease) OR TI=(hand surgery) OR TI=(hand operation) OR TI=(hand) OR TS=(hand condition) OR TS=(hand conditions) OR TS=(hand disease) OR TS=(hand disease) OR TS=(hand surgery) OR TS=(hand operation) OR TS=(hand)) AND (AB=(overview) OR AB=(review) OR AB=(conservative) OR AB=(conservative treatment) OR AB=(non-surgical) OR TI=(overview) OR TI=(review) OR TI=(conservative) OR TI=(conservative treatment) OR TI=(non-surgical) OR TS=(overview) OR TS=(review) OR TS=(conservative) OR TS=(conservative treatment) OR TS=(non-surgical))	117
2	(AB=(extracorporeal shockwave therapy) OR AB=(shockwave therapy) OR AB=(ESWT) OR AB=(SWT) OR AB=(shock wave) OR TI=(extracorporeal shockwave therapy) OR TI=(shockwave therapy) OR TI=(ESWT) OR TI=(SWT) OR TI=(shock wave) OR TS=(extracorporeal shockwave therapy) OR TS=(shockwave therapy) OR TS=(ESWT) OR TS=(SWT) OR TS=(shock wave)) AND (AB=(carpal tunnel syndrome) OR AB=(CTS) OR AB=(nerve compression syndrome) OR AB=(compression neuropathy) OR AB=(carpal canal syndrome) OR AB=(thenar carpal origin) OR AB=(amyotrophy carpal origin) OR AB=(distal median nerve compression) OR AB=(distal median nerve entrapment) OR AB=(entrapment neuropathy) OR AB=(median neuropathy) OR TI=(carpal tunnel syndrome) OR TI=(CTS) OR TI=(nerve compression syndrome) OR TI=(compression neuropathy) OR TI=(carpal canal syndrome) OR TI=(thenar carpal origin) OR TI=(amyotrophy carpal origin) OR TI=(distal median nerve compression) OR TI=(distal median nerve entrapment) OR TI=(entrapment neuropathy) OR TI=(median neuropathy) OR TS=(carpal tunnel syndrome) OR TS=(CTS) OR TS=(nerve compression syndrome) OR TS=(compression neuropathy) OR TS=(carpal canal syndrome) OR TS=(thenar carpal origin) OR TS=(amyotrophy carpal origin) OR TS=(distal median nerve compression) OR TS=(distal median nerve entrapment) OR TS=(entrapment neuropathy) OR TS=(median neuropathy))	67
3	(AB=(extracorporeal shockwave therapy) OR AB=(shockwave therapy) OR AB=(ESWT) OR AB=(SWT) OR AB=(shock wave) OR TI=(extracorporeal shockwave therapy) OR TI=(shockwave therapy) OR TI=(ESWT) OR TI=(SWT) OR TI=(shock wave) OR TS=(extracorporeal shockwave therapy) OR TS=(shockwave therapy) OR TS=(ESWT) OR TS=(SWT) OR TS=(shock wave)) AND (AB=(Trigger finger) OR AB=(digital stenosing tenosynovitis) OR AB=(stenosing tenosynovitis) OR AB=(trigger thumb) OR AB=(tendovaginitis stenosans) OR AB=(digital tendovaginitis stenosans) OR AB=(digitus saltans) OR AB=(digital flexor tenosynovitis) OR AB=(tendovaginitis finger) OR AB=(tenosynovitis stenosans) OR AB=(stenosing tendovaginitis) OR AB=(tenosynovitis finger) OR AB=(trigger digit) OR AB=(locked finger) OR TI=(Trigger finger) OR TI=(digital stenosing tenosynovitis) OR TI=(stenosing tenosynovitis) OR TI=(trigger thumb) OR	17

Search	Query	Number of Results
	TI=(tendovaginitis stenosans) OR TI=(digital tendovaginitis stenosans) OR TI=(digitus saltans) OR TI=(digital flexor tenosynovitis) OR TI=(tendovaginitis finger) OR TI=(tenosynovitis stenosans) OR TI=(stenosing tendovaginitis) OR TI=(tenosynovitis finger) OR TI=(trigger digit) OR TI=(locked finger) OR TS=(Trigger finger) OR TS=(digital stenosing tenosynovitis) OR TS=(stenosing tenosynovitis) OR TS=(trigger thumb) OR TS=(tendovaginitis stenosans) OR TS=(digital tendovaginitis stenosans) OR TS=(digitus saltans) OR TS=(digital flexor tenosynovitis) OR TS=(tendovaginitis finger) OR TS=(tenosynovitis stenosans) OR TS=(stenosing tendovaginitis) OR TS=(tenosynovitis finger) OR TS=(trigger digit) OR TS=(locked finger))	
4	(AB=(extracorporeal shockwave therapy) OR AB=(shockwave therapy) OR AB=(ESWT) OR AB=(SWT) OR AB=(shock wave) OR TI=(extracorporeal shockwave therapy) OR TI=(shockwave therapy) OR TI=(ESWT) OR TI=(SWT) OR TI=(shock wave) OR TS=(extracorporeal shockwave therapy) OR TS=(shockwave therapy) OR TS=(ESWT) OR TS=(SWT) OR TS=(shock wave)) AND (AB=(Dupuytren's disease) OR AB=(Dupuytren disease) OR AB=(Dupuytren's contracture) OR AB=(Dupuytren contracture) OR AB=(Morbus Dupuytren) OR AB=(contraction of palmar fascia) OR AB=(familial palmar fibromatosis) OR AB=(palmar fascial fibromatosis) OR AB=(palmar fibromatosis) OR AB=(Viking's disease) OR AB=(Viking disease) OR AB=(Celtic hand) OR TI=(Dupuytren's disease) OR TI=(Dupuytren disease) OR TI=(Dupuytren's contracture) OR TI=(Dupuytren contracture) OR TI=(Morbus Dupuytren) OR TI=(contraction of palmar fascia) OR TI=(familial palmar fibromatosis) OR TI=(palmar fascial fibromatosis) OR TI=(palmar fibromatosis) OR TI=(Viking's disease) OR TI=(Viking disease) OR TI=(Celtic hand) OR TS=(Dupuytren's disease) OR TS=(Dupuytren disease) OR TS=(Dupuytren's contracture) OR TS=(Dupuytren contracture) OR TS=(Morbus Dupuytren) OR TS=(contraction of palmar fascia) OR TS=(familial palmar fibromatosis) OR TS=(palmar fascial fibromatosis) OR TS=(palmar fibromatosis) OR TS=(Viking's disease) OR TS=(Viking disease) OR TS=(Celtic hand))	19
5	(AB=(extracorporeal shockwave therapy) OR AB=(shockwave therapy) OR AB=(ESWT) OR AB=(SWT) OR AB=(shock wave) OR TI=(extracorporeal shockwave therapy) OR TI=(shockwave therapy) OR TI=(ESWT) OR TI=(SWT) OR TI=(shock wave) OR TS=(extracorporeal shockwave therapy) OR TS=(shockwave therapy) OR TS=(ESWT) OR TS=(SWT) OR TS=(shock wave)) AND (AB=(DeQuervain's Syndrome) OR AB=(DeQuervain's Disease) OR AB=(DeQuervain's tendinopathy) OR AB=(DeQuervain's tenosynovitis) OR AB=(DeQuervain Syndrome) OR AB=(DeQuervain Disease) OR AB=(DeQuervain tendinopathy) OR AB=(DeQuervain tenosynovitis) OR AB=(De Quervain Syndrome) OR AB=(De Quervain disease) OR AB=(De Quervain tendinopathy) OR AB=(De Quervain tenosynovitis) OR AB=(Black berry thumb) OR AB=(texting thumb) OR AB=(gamer's thumb) OR AB=(gamer thumb) OR AB=(radial styloid tenosynovitis) OR AB=(washerman's sprain) OR AB=(washerwoman sprain) OR AB=(mother's wrist) OR AB=(mother wrist) OR AB=(mommy thumb) OR AB=(designer's thumb) OR AB=(designer thumb) OR AB=(DeQuervain's tendinitis) OR AB=(DeQuervain tendinitis) OR AB=(De Quervain tendinitis) OR TI=(DeQuervain's Syndrome) OR TI=(DeQuervain's Disease) OR TI=(DeQuervain's tendinopathy) OR TI=(DeQuervain's tenosynovitis) OR TI=(DeQuervain Syndrome) OR TI=(DeQuervain Disease) OR TI=(DeQuervain tendinopathy) OR TI=(DeQuervain tenosynovitis) OR TI=(De Quervain Syndrome) OR TI=(De Quervain disease) OR TI=(De Quervain tendinopathy) OR TI=(De Quervain tenosynovitis) OR TI=(Black berry thumb) OR TI=(texting thumb) OR TI=(gamer's thumb) OR TI=(gamer thumb) OR TI=(radial styloid tenosynovitis) OR TI=(washerman's sprain) OR TI=(washerwoman sprain) OR TI=(mother's wrist) OR TI=(mother wrist) OR TI=(mommy thumb) OR TI=(designer's thumb) OR TI=(designer thumb) OR TI=(DeQuervain's tendinitis) OR TI=(DeQuervain tendinitis) OR TI=(De Quervain tendinitis) OR TS=(DeQuervain's Syndrome) OR TS=(DeQuervain's Disease) OR TS=(DeQuervain's tendinopathy) OR TS=(DeQuervain's tenosynovitis) OR TS=(DeQuervain Syndrome) OR TS=(DeQuervain Disease) OR TS=(DeQuervain tendinopathy) OR TS=(DeQuervain tenosynovitis) OR TS=(De Quervain Syndrome) OR TS=(De Quervain disease)	3

Search	Query	Number of Results
	OR TS=(De Quervain tendinopathy) OR TS=(De Quervain tenosynovitis) OR TS=(Black berry thumb) OR TS=(texting thumb) OR TS=(gamer's thumb) OR TS=(gamer thumb) OR TS=(radial styloid tenosynovitis) OR TS=(washerman's sprain) OR TS=(washerwoman sprain) OR TS=(mother's wrist) OR TS=(mother wrist) OR TS=(mommy thumb) OR TS=(designer's thumb) OR TS=(designer thumb) OR TS=(DeQuervain's tendinitis) OR TS=(DeQuervain tendinitis) OR TS=(De Quervain tendinitis))	
6	(AB=(extracorporeal shockwave therapy) OR AB=(shockwave therapy) OR AB=(ESWT) OR AB=(SWT) OR AB=(shock wave) OR TI=(extracorporeal shockwave therapy) OR TI=(shockwave therapy) OR TI=(ESWT) OR TI=(SWT) OR TI=(shock wave) OR TS=(extracorporeal shockwave therapy) OR TS=(shockwave therapy) OR TS=(ESWT) OR TS=(SWT) OR TS=(shock wave)) AND (AB=(Kienböck's disease) OR AB=(Kienböck disease) OR AB=(Kienbock disease) OR AB=(Kienbock) OR AB=(Kienböck) OR AB=(osteonecrosis of lunate) OR AB=(osteonecrosis lunate bone) OR AB=(aseptic necrosis lunate) OR AB=(osteonecrosis carpal lunate) OR AB=(avascular necrosis lunate) OR AB=(lunatomalacia) OR AB=(osteochondrisis lunate) OR AB=(progressive avascular necrosis lunate) OR AB=(progressive aseptic necrosis lunate) OR AB=(progressive osteonecrosis lunate) OR TI=(Kienböck's disease) OR TI=(Kienböck disease) OR TI=(Kienbock disease) OR TI=(Kienbock) OR TI=(Kienböck) OR TI=(osteonecrosis of lunate) OR TI=(osteonecrosis lunate bone) OR TI=(aseptic necrosis lunate) OR TI=(osteonecrosis carpal lunate) OR TI=(avascular necrosis lunate) OR TI=(lunatomalacia) OR TI=(osteochondrisis lunate) OR TI=(progressive avascular necrosis lunate) OR TI=(progressive aseptic necrosis lunate) OR TI=(progressive osteonecrosis lunate) OR TS=(Kienböck's disease) OR TS=(Kienböck disease) OR TS=(Kienbock disease) OR TS=(Kienbock) OR TS=(Kienböck) OR TS=(osteonecrosis of lunate) OR TS=(osteonecrosis lunate bone) OR TS=(aseptic necrosis lunate) OR TS=(osteonecrosis carpal lunate) OR TS=(avascular necrosis lunate) OR TS=(lunatomalacia) OR TS=(osteochondrisis lunate) OR TS=(progressive avascular necrosis lunate) OR TS=(progressive aseptic necrosis lunate) OR TS=(progressive osteonecrosis lunate))	4
	**Total**	1,622

MEDLINE (U.S. National Library of Medicine, National Institutes of Health): September 2, 2024

Search	Query	Number of Results
1	(Extracorporeal Shockwave Therap* OR shock wave OR ESWT OR SWT) AND (hand condition* OR hand disease* OR hand surger* OR hand operation* OR hand*)	173
2	Hand* AND (Extracorporeal Shockwave Therap* OR shock wave OR ESWT OR SWT) AND (overview* OR review OR conservative* OR conservative treatment* OR non-surgical)	52
3	(Extracorporeal Shockwave Therap* OR shock wave OR ESWT OR SWT) AND (carpal tunnel syndrome* OR CTS OR nerve compression* syndrome* OR compression neuropathy OR carpal canal syndrome* OR thenar* of carpal origin* OR amyotrophy carpal origin* OR distal median nerve* compression* OR distal median nerve* entrapment* OR entrapment* neuropathy OR median neuropathy)	36
4	(Extracorporeal Shockwave Therap* OR shock wave OR ESWT OR SWT) AND (Trigger finger* OR digital stenosing tenosynovitis OR stenosing tenosynovitis OR trigger thumb* OR tendovaginitis stenosans OR digital tendovaginitis stenosans OR digitus saltans OR digital flexor tenosynovitis OR tendovaginitis finger* OR tenosynovitis stenosans OR stenosing tendovaginitis OR tenosynovitis finger* OR trigger digit* OR lock* finger*)	11
5	(Extracorporeal Shockwave Therap* OR shock wave OR ESWT OR SWT) AND (Dupuytren* Disease* OR Dupuytren* contracture* OR Morbus Dupuytren OR contraction* of palmar fascia OR familial palmar fibromatosis OR palmar fascial fibromatosis OR palmar fibromatosis OR Viking* Disease* OR Celtic* hand*)	9

Search	Query	Number of Results
6	(Extracorporeal Shockwave Therap* OR shock wave OR ESWT OR SWT) AND (DeQuervain* Syndrome* OR DeQuervain* Disease OR DeQuervain* tendinopath* OR DeQuervain* tenosynovitis OR De Quervain* Syndrome* OR De Quervain* disease* OR De Quervain* tendinopath* OR De Quervain* tenosynovitis OR Black berry thumb* OR texting thumb* OR gamer* thumb* OR radial styloid tenosynovitis OR washerman* sprain* OR mother* wrist* OR momm* thumb* OR designer* thumb* OR DeQuervain* tendinitis OR De Quervain* tendinitis)	2
7	(Extracorporeal Shockwave Therap* OR shock wave OR ESWT OR SWT) AND (Kienb#ck* disease OR Kienb#ck OR osteonecrosis of lunate OR osteonecrosis lunate bone OR osteonecrosis carpal lunate OR avascular necrosis lunate OR aseptic necrosis lunate OR lunatomalacia OR osteochondrisis lunate OR progressive avascular necrosis lunate OR progressive aseptic necrosis lunate OR progressive osteonecrosis lunate)	2
	**Total**	**285**

CINAHL Ultimate (EBSCO): September 2, 2024

Search	Query	Number of Results
1	(extracorporeal shockwave therapy OR shockwave therapy OR ESWT OR SWT) AND (hand condition OR hand conditions OR hand disease OR hand disease OR hand surgery OR hand operation OR hand)	40
2	(Extracorporeal Shockwave Therapy OR shock wave OR ESWT OR SWT) AND (overview OR review OR conservative OR conservative treatment OR non-surgical)	551
3	(extracorporeal shockwave therapy OR shock wave OR ESWT OR SWT) AND (carpal tunnel syndrome OR CTS OR nerve compression syndrome OR compression neuropathy OR carpal canal syndrome OR thenar of carpal origin OR amyotrophy carpal origin OR distal median nerve compression OR distal median nerve entrapment OR entrapment neuropathy OR median neuropathy)	28
4	(extracorporeal shockwave therapy OR shock wave OR ESWT OR SWT) AND (Trigger finger OR digital stenosing tenosynovitis OR stenosing tenosynovitis OR trigger thumb OR tendovaginitis stenosans OR digital tendovaginitis stenosans OR digitus saltans OR digital flexor tenosynovitis OR tendovaginitis finger OR tenosynovitis stenosans OR stenosing tendovaginitis OR tenosynovitis finger OR trigger digit OR locked finger)	9
5	(extracorporeal shockwave therapy OR shock wave OR ESWT OR SWT) AND (Dupuytren's Disease OR Dupuytren Disease OR Dupytren's contracture OR Dupuytren contracture OR Morbus Dupuytren OR contraction of palmar fascia OR familial palmar fibromatosis OR palmar fascial fibromatosis OR palmar fibromatosis OR Viking's Disease OR Viking Disease OR Celtic hand)	5
6	(extracorporeal shockwave therapy OR shock wave OR ESWT OR SWT) AND (DeQuervain's Syndrome OR DeQuervain's Disease OR DeQuervain's tendinopathy OR DeQuervain's tenosynovitis OR DeQuervain Syndrome OR DeQuervain Disease OR DeQuervain tendinopathy OR DeQuervain tenosynovitis OR De Quervain Syndrome OR De Quervain disease OR De Quervain tendinopathy OR De Quervain tenosynovitis OR Black berry thumb OR texting thumb OR gamer's thumb OR gamer thumb OR radial styloid tenosynovitis OR washerman's sprain OR washerwoman sprain OR mother's wrist OR mother wrist OR mommy thumb OR designer's thumb OR designer thumb OR DeQuervain's tendinitis OR DeQuervain tendinitis OR De Quervain tendinitis)	2
7	(extracorporeal shockwave therapy OR shock wave OR ESWT OR SWT) AND (Kienböck's disease OR Kienböck disease OR Kienbock disease OR Kienbock OR Kienböck OR osteonecrosis of lunate OR osteonecrosis lunate bone OR osteonecrosis carpal lunate OR avascular necrosis lunate OR aseptic necrosis lunate OR lunatomalacia OR osteochondrisis lunate OR progressive avascular necrosis lunate OR progressive aseptic necrosis lunate OR progressive osteonecrosis	25

Search	Query	Number of Results
	lunate)	
	**Total**	660

Cochrane Library (Cochrane Collaboration): September 2, 2024

Search	Query	Number of Results
1	(Extracorporeal Shockwave Therap* OR shock wave OR ESWT OR SWT) AND (hand condition* OR hand disease* OR hand surger* OR hand operation* OR hand*)	251
2	Hand* AND (Extracorporeal Shockwave Therap* OR shock wave OR ESWT OR SWT) AND (overview* OR review OR conservative* OR conservative treatment* OR non-surgical)	52
3	(Extracorporeal Shockwave Therap* OR shock wave OR ESWT OR SWT) AND (carpal tunnel syndrome* OR CTS OR nerve compression* syndrome* OR compression neuropathy OR carpal canal syndrome* OR thenar* of carpal origin* OR amyotrophy carpal origin* OR distal median nerve* compression* OR distal median nerve* entrapment* OR entrapment* neuropathy OR median neuropathy)	81
4	(Extracorporeal Shockwave Therap* OR shock wave OR ESWT OR SWT) AND (Trigger finger* OR digital stenosing tenosynovitis OR stenosing tenosynovitis OR trigger thumb* OR tendovaginitis stenosans OR digital tendovaginitis stenosans OR digitus saltans OR digital flexor tenosynovitis OR tendovaginitis finger* OR tenosynovitis stenosans OR stenosing tendovaginitis OR tenosynovitis finger* OR trigger digit* OR lock* finger*)	24
5	(Extracorporeal Shockwave Therap* OR shock wave OR ESWT OR SWT) AND (Dupuytren* Disease* OR Dupuytren* contracture* OR Morbus Dupuytren OR contraction* of palmar fascia OR familial palmar fibromatosis OR palmar fascial fibromatosis OR palmar fibromatosis OR Viking* Disease* OR Celtic* hand*)	7
6	(Extracorporeal Shockwave Therap* OR shock wave OR ESWT OR SWT) AND (DeQuervain* Syndrome* OR DeQuervain* Disease OR DeQuervain* tendinopath* OR DeQuervain* tenosynovitis OR De Quervain* Syndrome* OR De Quervain* disease* OR De Quervain* tendinopath* OR De Quervain* tenosynovitis OR Black berry thumb* OR texting thumb* OR gamer* thumb* OR radial styloid tenosynovitis OR washerman* sprain* OR mother* wrist* OR momm* thumb* OR designer* thumb* OR DeQuervain* tendinitis OR De Quervain* tendinitis)	8
7	(Extracorporeal Shockwave Therap* OR shock wave OR ESWT OR SWT) AND (Kienb#ck* disease OR Kienb#ck OR osteonecrosis of lunate OR osteonecrosis lunate bone OR osteonecrosis carpal lunate OR avascular necrosis lunate OR aseptic necrosis lunate OR lunatomalacia OR osteochondrisis lunate OR progressive avascular necrosis lunate OR progressive aseptic necrosis lunate OR progressive osteonecrosis lunate)	0
	**Total**	423

Google Scholar (Google LLC): September 2, 2024

Search	Query	Number of Results
1	(extracorporeal shockwave therapy OR shockwave therapy OR ESWT OR SWT) AND (hand condition OR hand conditions OR hand disease OR hand disease OR hand surgery OR hand operation OR hand) NOT (books AND comment AND document)	1,290
2	(Extracorporeal Shockwave Therapy OR shock wave OR ESWT OR SWT) AND (overview OR review OR conservative OR conservative treatment OR non-surgical) NOT (books AND comment AND document)	851
3	(extracorporeal shockwave therapy OR shock wave OR ESWT OR SWT) AND (carpal tunnel syndrome OR CTS OR nerve compression syndrome OR compression neuropathy OR carpal	56

Search	Query	Number of Results
	canal syndrome OR thenar of carpal origin OR amyotrophy carpal origin OR distal median nerve compression OR distal median nerve entrapment OR entrapment neuropathy OR median neuropathy)	
4	(extracorporeal shockwave therapy OR shock wave OR ESWT OR SWT) AND (Trigger finger OR digital stenosing tenosynovitis OR stenosing tenosynovitis OR trigger thumb OR tendovaginitis stenosans OR digital tendovaginitis stenosans OR digitus saltans OR digital flexor tenosynovitis OR tendovaginitis finger OR tenosynovitis stenosans OR stenosing tendovaginitis OR tenosynovitis finger OR trigger digit OR locked finger)	2
5	(extracorporeal shockwave therapy OR shock wave OR ESWT OR SWT) AND (Dupuytren’s Disease OR Dupuytren Disease OR Dupytren’s contracture OR Dupuytren contracture OR Morbus Dupuytren OR contraction of palmar fascia OR familial palmar fibromatosis OR palmar fascial fibromatosis OR palmar fibromatosis OR Viking’s Disease OR Viking Disease OR Celtic hand)	0
6	(extracorporeal shockwave therapy OR shock wave OR ESWT OR SWT) AND (DeQuervain’s Syndrome OR DeQuervain’s Disease OR DeQuervain’s tendinopathy OR DeQuervain’s tenosynovitis OR DeQuervain Syndrome OR DeQuervain Disease OR DeQuervain tendinopathy OR DeQuervain tenosynovitis OR De Quervain Syndrome OR De Quervain disease OR De Quervain tendinopathy OR De Quervain tenosynovitis OR Black berry thumb OR texting thumb OR gamer’s thumb OR gamer thumb OR radial styloid tenosynovitis OR washerman’s sprain OR washerwoman sprain OR mother’s wrist OR mother wrist OR mommy thumb OR designer’s thumb OR designer thumb OR DeQuervain’s tendinitis OR DeQuervain tendinitis OR De Quervain tendinitis)	1
7	(extracorporeal shockwave therapy OR shock wave OR ESWT OR SWT) AND (Kienböck’s disease OR Kienböck disease OR Kienbock disease OR Kienbock OR Kienböck OR osteonecrosis of lunate OR osteonecrosis lunate bone OR osteonecrosis carpal lunate OR avascular necrosis lunate OR aseptic necrosis lunate OR lunatomalacia OR osteochondrisis lunate OR progressive avascular necrosis lunate OR progressive aseptic necrosis lunate OR progressive osteonecrosis lunate)	6
	**Total**	2,206

ScienceDirect (Elsevier): September 3, 2024

Search	Query	Number of Results
1	(extracorporeal shockwave therapy OR ESWT OR SWT) AND (hand condition OR hand disease OR hand surgery OR hand operation)	871
2	(extracorporeal shockwave therapy OR ESWT OR SWT) AND (carpal tunnel syndrome OR CTS OR nerve compression syndrome OR compression neuropathy OR carpal canal syndrome)	379
3	(extracorporeal shockwave therapy OR ESWT OR SWT) AND (thenar of carpal origin OR amyotrophy carpal origin OR distal median nerve compression OR distal median nerve entrapment OR entrapment neuropathy OR median neuropathy)	229
4	(extracorporeal shockwave therapy OR shock wave OR ESWT OR SWT) AND (Trigger finger OR trigger thumb OR trigger digit OR locked finger)	1,132
5	(extracorporeal shockwave therapy OR shock wave OR ESWT OR SWT) AND (stenosing tenosynovitis OR digital flexor tenosynovitis OR tendovaginitis finger OR stenosing tendovaginitis OR tenosynovitis finger)	53
6	(Extracorporeal Shockwave Therapy OR shock wave OR ESWT OR SWT) AND (dupuytren OR dupuytren's contracture OR palmar fibromatosis OR Morbus Dupuytren)	308
7	(extracorporeal shockwave therapy OR ESWT OR SWT) AND (DeQuervain Syndrome OR DeQuervain Disease OR DeQuervain tendinopathy OR De Quervain Syndrome OR De Quervain	29

Search	Query	Number of Results
	disease OR De Quervain tendinopathy)	
8	(extracorporeal shockwave therapy OR ESWT OR SWT) AND (texting thumb OR gamer thumb OR washerwoman sprain OR mother wrist OR mommy thumb OR designer thumb)	54
9	(extracorporeal shockwave therapy OR ESWT OR SWT) AND (Kienböck OR Kienbock disease OR Kienbock OR osteonecrosis lunate OR osteonecrosis carpal lunate)	17
10	(extracorporeal shockwave therapy OR ESWT OR SWT) AND (avascular necrosis lunate OR aseptic necrosis lunate OR lunatomalacia OR osteochondrisis lunate)	8
	**Total**	**3,080**

PubMed Clinical Queries (U.S. National Library of Medicine, National Institutes of Health): September 3, 2024

Search	Query	Number of Results
1	("Extracorporeal Shockwave Therapy"[Mesh] OR extracorporeal shockwave therapy[tiab] OR shockwave therapy[tiab] OR ESWT[tiab] OR SWT[tiab] AND (hand condition[tiab] OR hand conditions[tiab] OR hand disease[tiab] OR hand disease[tiab] OR hand surgery[tiab] OR hand operation[tiab] OR hand[tiab]))	63
2	(“Hand”[Mesh] AND ("Extracorporeal Shockwave Therapy"[Mesh] OR extracorporeal shockwave therapy[tiab] OR shock wave[tiab] OR ESWT[tiab] OR SWT[tiab]) AND (overview[tiab] OR review[tiab] OR conservative[tiab] OR conservative treatment[tiab] OR non-surgical[tiab]))	2
3	(("Extracorporeal Shockwave Therapy"[Mesh] OR "Hand"[Mesh] OR extracorporeal shockwave therapy[tiab] OR shock wave[tiab] OR ESWT[tiab] OR SWT[tiab]) AND (carpal tunnel syndrome[tiab] OR CTS[tiab] OR nerve compression syndrome[tiab] OR compression neuropathy[tiab] OR carpal canal syndrome[tiab] OR thenar of carpal origin[tiab] OR amyotrophy carpal origin[tiab] OR distal median nerve compression[tiab] OR distal median nerve entrapment[tiab] OR entrapment neuropathy[tiab] OR median neuropathy[tiab]))	240
4	(("Extracorporeal Shockwave Therapy"[Mesh] OR "Hand"[Mesh] OR extracorporeal shockwave therapy[tiab] OR shock wave[tiab] OR ESWT[tiab] OR SWT[tiab]) AND (Trigger finger[tiab] OR digital stenosing tenosynovitis[tiab] OR stenosing tenosynovitis[tiab] OR trigger thumb[tiab] OR tendovaginitis stenosans[tiab] OR digital tendovaginitis stenosans[tiab] OR digitus saltans[tiab] OR digital flexor tenosynovitis[tiab] OR tendovaginitis finger[tiab] OR tenosynovitis stenosans[tiab] OR stenosing tendovaginitis[tiab] OR tenosynovitis finger[tiab] OR trigger digit[tiab] OR locked finger[tiab]))	137
5	(("Extracorporeal Shockwave Therapy"[Mesh] OR "Hand"[Mesh] OR extracorporeal shockwave therapy[tiab] OR shock wave[tiab] OR ESWT[tiab] OR SWT[tiab]) AND (Dupuytren’s Disease[tiab] OR Dupuytren Disease[tiab] OR Dupytren’s contracture[tiab] OR Dupuytren contracture[tiab] OR Morbus Dupuytren[tiab] OR contraction of palmar fascia[tiab] OR familial palmar fibromatosis[tiab] OR palmar fascial fibromatosis[tiab] OR palmar fibromatosis[tiab] OR Viking’s Disease[tiab] OR Viking Disease[tiab] OR Celtic hand[tiab]))	53
6	(("Extracorporeal Shockwave Therapy"[Mesh] OR "Hand"[Mesh] OR extracorporeal shockwave therapy[tiab] OR shock wave[tiab] OR ESWT[tiab] OR SWT[tiab]) AND (DeQuervain’s Syndrome[tiab] OR DeQuervain’s Disease[tiab] OR DeQuervain’s tendinopathy[tiab] OR DeQuervain’s tenosynovitis[tiab] OR DeQuervain Syndrome[tiab] OR DeQuervain Disease[tiab] OR DeQuervain tendinopathy[tiab] OR DeQuervain tenosynovitis[tiab] OR De Quervain Syndrome[tiab] OR De Quervain disease[tiab] OR De Quervain tendinopathy[tiab] OR De Quervain tenosynovitis[tiab] OR Black berry thumb[tiab] OR texting thumb[tiab] OR gamer’s thumb[tiab] OR gamer thumb[tiab] OR radial styloid tenosynovitis[tiab] OR washerman’s sprain[tiab] OR washerwoman sprain[tiab] OR mother’s wrist[tiab] OR mother wrist[tiab] OR mommy thumb[tiab] OR designer’s thumb[tiab] OR designer thumb[tiab] OR DeQuervain’s	171

Search	Query	Number of Results
	tendinitis[tiab] OR DeQuervain tendinitis[tiab] OR De Quervain tendinitis[tiab]))	
7	(("Extracorporeal Shockwave Therapy"[Mesh] OR "Hand"[Mesh] OR extracorporeal shockwave therapy[tiab] OR shock wave[tiab] OR ESWT[tiab] OR SWT[tiab]) AND (Kienböck's disease[tiab] OR Kienböck disease[tiab] OR Kienbock disease[tiab] OR Kienbock[tiab] OR Kienböck[tiab] OR osteonecrosis of lunate[tiab] OR osteonecrosis lunate bone[tiab] OR osteonecrosis carpal lunate[tiab] OR avascular necrosis lunate[tiab] OR aseptic necrosis lunate[tiab] OR lunatomalacia[tiab] OR osteochondrisis lunate[tiab] OR progressive avascular necrosis lunate[tiab] OR progressive aseptic necrosis lunate [tiab] OR progressive osteonecrosis lunate[tiab]))	5
	**Total**	**671**

PubMed Central (U.S. National Library of Medicine, National Institutes of Health): September 4, 2024

Search	Query	Number of Results
1	"Extracorporeal Shockwave Therapy"[Mesh] OR ("extracorporeal shockwave therapy"[MeSH Terms] OR ("extracorporeal"[All Fields] AND "shockwave"[All Fields] AND "therapy"[All Fields]) OR "extracorporeal shockwave therapy"[All Fields]) OR shockwave therapy[All Fields] OR ESWT[All Fields] OR SWT[All Fields] AND (hand condition[All Fields] OR hand conditions[All Fields] OR hand disease[All Fields] OR hand disease[All Fields] OR hand surgery[All Fields] OR hand operation[All Fields] OR ("hand"[MeSH Terms] OR "hand"[All Fields]))	3,053
2	"Hand"[Mesh] AND ("Extracorporeal Shockwave Therapy"[Mesh] OR ("extracorporeal shockwave therapy"[MeSH Terms] OR ("extracorporeal"[All Fields] AND "shockwave"[All Fields] AND "therapy"[All Fields]) OR "extracorporeal shockwave therapy"[All Fields]) OR shock wave[All Fields] OR ESWT[All Fields] OR SWT[All Fields]) AND (overview[All Fields] OR ("review"[All Fields] OR "review literature as topic"[MeSH Terms] OR "review"[All Fields]) OR conservative[All Fields] OR ("conservative treatment"[MeSH Terms] OR ("conservative"[All Fields] AND "treatment"[All Fields]) OR "conservative treatment"[All Fields]) OR non-surgical[All Fields])	9
3	("Extracorporeal Shockwave Therapy"[Mesh] OR "Hand"[Mesh] OR ("extracorporeal shockwave therapy"[MeSH Terms] OR ("extracorporeal"[All Fields] AND "shockwave"[All Fields] AND "therapy"[All Fields]) OR "extracorporeal shockwave therapy"[All Fields]) OR shock wave[All Fields] OR ESWT[All Fields] OR SWT[All Fields]) AND (("carpal tunnel syndrome"[MeSH Terms] OR ("carpal"[All Fields] AND "tunnel"[All Fields] AND "syndrome"[All Fields]) OR "carpal tunnel syndrome"[All Fields]) OR CTS[All Fields] OR ("nerve compression syndromes"[MeSH Terms] OR ("nerve"[All Fields] AND "compression"[All Fields] AND "syndromes"[All Fields]) OR "nerve compression syndromes"[All Fields] OR ("nerve"[All Fields] AND "compression"[All Fields] AND "syndrome"[All Fields]) OR "nerve compression syndrome"[All Fields]) OR ("Tomaculous neuropathy"[All Fields] OR "compression neuropathy"[All Fields] OR "nerve compression syndromes"[MeSH Terms] OR ("nerve"[All Fields] AND "compression"[All Fields] AND "syndromes"[All Fields]) OR "nerve compression syndromes"[All Fields] OR ("compression"[All Fields] AND "neuropathy"[All Fields])) OR carpal canal syndrome[All Fields] OR ("carpal tunnel syndrome"[MeSH Terms] OR ("carpal"[All Fields] AND "tunnel"[All Fields] AND "syndrome"[All Fields]) OR "carpal tunnel syndrome"[All Fields] OR ("distal"[All Fields] AND "median"[All Fields] AND "nerve"[All Fields] AND "compression"[All Fields]) OR "distal median nerve compression"[All Fields]) OR ("carpal tunnel syndrome"[MeSH Terms] OR ("carpal"[All Fields] AND "tunnel"[All Fields] AND "syndrome"[All Fields]) OR "carpal tunnel syndrome"[All Fields] OR ("distal"[All Fields] AND "median"[All Fields] AND "nerve"[All Fields] AND "entrapment"[All Fields])) OR ("nerve compression syndromes"[MeSH Terms] OR ("nerve"[All Fields] AND "compression"[All Fields] AND "syndromes"[All Fields]) OR "nerve compression syndromes"[All Fields] OR ("entrapment"[All Fields] AND "neuropathy"[All	2,026

Search	Query	Number of Results
	Fields]) OR "entrapment neuropathy"[All Fields]) OR ("median neuropathy"[MeSH Terms] OR ("median"[All Fields] AND "neuropathy"[All Fields]) OR "median neuropathy"[All Fields]))	
4	("Extracorporeal Shockwave Therapy"[Mesh] OR "Hand"[Mesh] OR ("extracorporeal shockwave therapy"[MeSH Terms] OR ("extracorporeal"[All Fields] AND "shockwave"[All Fields] AND "therapy"[All Fields]) OR "extracorporeal shockwave therapy"[All Fields]) OR shock wave[All Fields] OR ESWT[All Fields] OR SWT[All Fields]) AND (("trigger finger disorder"[MeSH Terms] OR ("trigger"[All Fields] AND "finger"[All Fields] AND "disorder"[All Fields]) OR "trigger finger disorder"[All Fields] OR ("trigger"[All Fields] AND "finger"[All Fields]) OR "trigger finger"[All Fields]) OR digital stenosing tenosynovitis[All Fields] OR ("tendon entrapment"[MeSH Terms] OR ("tendon"[All Fields] AND "entrapment"[All Fields]) OR "tendon entrapment"[All Fields] OR ("stenosing"[All Fields] AND "tenosynovitis"[All Fields]) OR "stenosing tenosynovitis"[All Fields]) OR ("trigger finger disorder"[MeSH Terms] OR ("trigger"[All Fields] AND "finger"[All Fields] AND "disorder"[All Fields]) OR "trigger finger disorder"[All Fields] OR ("trigger"[All Fields] AND "thumb"[All Fields]) OR "trigger thumb"[All Fields]) OR tendovaginitis stenosans[All Fields] OR digitus saltans[All Fields] OR ("trigger finger disorder"[MeSH Terms] OR ("trigger"[All Fields] AND "finger"[All Fields] AND "disorder"[All Fields]) OR "trigger finger disorder"[All Fields] OR ("digital"[All Fields] AND "flexor"[All Fields] AND "tenosynovitis"[All Fields]) OR "digital flexor tenosynovitis"[All Fields]) OR ("de quervain disease"[MeSH Terms] OR ("de"[All Fields] AND "quervain"[All Fields] AND "disease"[All Fields]) OR "de quervain disease"[All Fields] OR ("tenosynovitis"[All Fields] AND "stenosans"[All Fields]) OR "tenosynovitis stenosans"[All Fields]) OR ("tendon entrapment"[MeSH Terms] OR ("tendon"[All Fields] AND "entrapment"[All Fields]) OR "tendon entrapment"[All Fields] OR ("stenosing"[All Fields] AND "tendovaginitis"[All Fields]) OR "stenosing tendovaginitis"[All Fields]) OR ("trigger finger disorder"[MeSH Terms] OR ("trigger"[All Fields] AND "finger"[All Fields] AND "disorder"[All Fields]) OR "trigger finger disorder"[All Fields] OR ("trigger"[All Fields] AND "digit"[All Fields]) OR "trigger digit"[All Fields]) OR locked finger[All Fields])	1,513
5	("Extracorporeal Shockwave Therapy"[Mesh] OR "Hand"[Mesh] OR ("extracorporeal shockwave therapy"[MeSH Terms] OR ("extracorporeal"[All Fields] AND "shockwave"[All Fields] AND "therapy"[All Fields]) OR "extracorporeal shockwave therapy"[All Fields]) OR shock wave[All Fields] OR ESWT[All Fields] OR SWT[All Fields]) AND (("dupuytren contracture"[MeSH Terms] OR ("dupuytren"[All Fields] AND "contracture"[All Fields]) OR "dupuytren contracture"[All Fields] OR ("dupuytren's"[All Fields] AND "disease"[All Fields]) OR "dupuytren's disease"[All Fields]) OR ("dupuytren contracture"[MeSH Terms] OR ("dupuytren"[All Fields] AND "contracture"[All Fields]) OR "dupuytren contracture"[All Fields] OR ("dupuytren"[All Fields] AND "disease"[All Fields]) OR "dupuytren disease"[All Fields]) OR ("dupuytren contracture"[MeSH Terms] OR ("dupuytren"[All Fields] AND "contracture"[All Fields]) OR "dupuytren contracture"[All Fields] OR ("dupytren's"[All Fields] AND "contracture"[All Fields]) OR "dupytren's contracture"[All Fields]) OR ("dupuytren contracture"[MeSH Terms] OR ("dupuytren"[All Fields] AND "contracture"[All Fields]) OR "dupuytren contracture"[All Fields]) OR Morbus Dupuytren[All Fields] OR ("dupuytren contracture"[MeSH Terms] OR ("dupuytren"[All Fields] AND "contracture"[All Fields]) OR "dupuytren contracture"[All Fields] OR ("palmar"[All Fields] AND "fascial"[All Fields] AND "fibromatosis"[All Fields]) OR "palmar fascial fibromatosis"[All Fields]) OR ("dupuytren contracture"[MeSH Terms] OR ("dupuytren"[All Fields] AND "contracture"[All Fields]) OR "dupuytren contracture"[All Fields] OR ("palmar"[All Fields] AND "fibromatosis"[All Fields]) OR "palmar fibromatosis"[All Fields]) OR Viking's Disease[All Fields] OR Viking Disease[All Fields])	317
6	("Extracorporeal Shockwave Therapy"[Mesh] OR "Hand"[Mesh] OR ("extracorporeal shockwave therapy"[MeSH Terms] OR ("extracorporeal"[All Fields] AND "shockwave"[All Fields] AND "therapy"[All Fields]) OR "extracorporeal shockwave therapy"[All Fields]) OR shock wave[All Fields] OR ESWT[All Fields] OR SWT[All Fields]) AND (DeQuervain's Syndrome[All	96

Search	Query	Number of Results
	Fields] OR DeQuervain's Disease[All Fields] OR DeQuervain's tenosynovitis[All Fields] OR DeQuervain Syndrome[All Fields] OR DeQuervain Disease[All Fields] OR DeQuervain tenosynovitis[All Fields] OR De Quervain Syndrome[All Fields] OR ("de quervain disease"[MeSH Terms] OR ("de"[All Fields] AND "quervain"[All Fields] AND "disease"[All Fields]) OR "de quervain disease"[All Fields]) OR De Quervain tendinopathy[All Fields] OR ("de quervain disease"[MeSH Terms] OR ("de"[All Fields] AND "quervain"[All Fields] AND "disease"[All Fields]) OR "de quervain disease"[All Fields] OR ("de"[All Fields] AND "quervain"[All Fields] AND "tenosynovitis"[All Fields]) OR "de quervain tenosynovitis"[All Fields]) OR texting thumb[All Fields] OR gamer's thumb[All Fields] OR ("de quervain disease"[MeSH Terms] OR ("de"[All Fields] AND "quervain"[All Fields] AND "disease"[All Fields]) OR "de quervain disease"[All Fields] OR ("radial"[All Fields] AND "styloid"[All Fields] AND "tenosynovitis"[All Fields]) OR "radial styloid tenosynovitis"[All Fields]) OR mother's wrist[All Fields] OR De Quervain tendinitis[All Fields])	
7	("Extracorporeal Shockwave Therapy"[Mesh] OR "Hand"[Mesh] OR ("extracorporeal shockwave therapy"[MeSH Terms] OR ("extracorporeal"[All Fields] AND "shockwave"[All Fields] AND "therapy"[All Fields]) OR "extracorporeal shockwave therapy"[All Fields]) OR shock wave[All Fields] OR ESWT[All Fields] OR SWT[All Fields]) AND (("osteonecrosis"[MeSH Terms] OR "osteonecrosis"[All Fields] OR ("kienbock's"[All Fields] AND "disease"[All Fields]) OR "kienbock's disease"[All Fields]) OR ("osteonecrosis"[MeSH Terms] OR "osteonecrosis"[All Fields] OR ("kienbock"[All Fields] AND "disease"[All Fields]) OR "kienbock disease"[All Fields]) OR ("osteonecrosis"[MeSH Terms] OR "osteonecrosis"[All Fields] OR ("kienbock"[All Fields] AND "disease"[All Fields]) OR "kienbock disease"[All Fields]) OR Kienbock[All Fields] OR Kienbock[All Fields] OR ("osteonecrosis"[MeSH Terms] OR "osteonecrosis"[All Fields] OR "lunatomalacia"[All Fields]))	495
	**Total**	**7,509**

Joanna Briggs Institute EBP Database (Ovid): September 4, 2024

Search	Query	Number of Results
1	(Extracorporeal Shockwave Therap* OR shock wave OR ESWT OR SWT) AND (hand condition* OR hand disease* OR hand surger* OR hand operation* OR hand*)	9
2	Hand* AND (Extracorporeal Shockwave Therap* OR shock wave OR ESWT OR SWT) AND (overview* OR review OR conservative* OR conservative treatment* OR non-surgical)	9
3	(Extracorporeal Shockwave Therap* OR shock wave OR ESWT OR SWT) AND (carpal tunnel syndrome* OR CTS OR nerve compression* syndrome* OR compression neuropathy OR carpal canal syndrome* OR thenar* of carpal origin* OR amyotrophy carpal origin* OR distal median nerve* compression* OR distal median nerve* entrapment* OR entrapment* neuropathy OR median neuropathy)	2
4	(Extracorporeal Shockwave Therap* OR shock wave OR ESWT OR SWT) AND (Trigger finger* OR digital stenosing tenosynovitis OR stenosing tenosynovitis OR trigger thumb* OR tendovaginitis stenosans OR digital tendovaginitis stenosans OR digitus saltans OR digital flexor tenosynovitis OR tendovaginitis finger* OR tenosynovitis stenosans OR stenosing tendovaginitis OR tenosynovitis finger* OR trigger digit* OR lock* finger*)	0
5	(Extracorporeal Shockwave Therap* OR shock wave OR ESWT OR SWT) AND (Dupuytren* Disease* OR Dupuytren* contracture* OR Morbus Dupuytren OR contraction* of palmar fascia OR familial palmar fibromatosis OR palmar fascial fibromatosis OR palmar fibromatosis OR Viking* Disease* OR Celtic* hand*)	1
6	(Extracorporeal Shockwave Therap* OR shock wave OR ESWT OR SWT) AND (DeQuervain* Syndrome* OR DeQuervain* Disease OR DeQuervain* tendinopath* OR DeQuervain*	0

Search	Query	Number of Results
	tenosynovitis OR De Quervain* Syndrome* OR De Quervain* disease* OR De Quervain* tendinopath* OR De Quervain* tenosynovitis OR Black berry thumb* OR texting thumb* OR gamer* thumb* OR radial styloid tenosynovitis OR washerman* sprain* OR mother* wrist* OR momm* thumb* OR designer* thumb* OR DeQuervain* tendinitis OR De Quervain* tendinitis)	
7	(Extracorporeal Shockwave Therap* OR shock wave OR ESWT OR SWT) AND (Kienb#ck* disease OR Kienb#ck OR osteonecrosis of lunate OR osteonecrosis lunate bone OR osteonecrosis carpal lunate OR avascular necrosis lunate OR aseptic necrosis lunate OR lunatomalacia OR osteochondrisis lunate OR progressive avascular necrosis lunate OR progressive aseptic necrosis lunate OR progressive osteonecrosis lunate)	0
	**Total**	21

Oxford Journals Current Content (Oxford University Press): September 4, 2024

Search	Query	Number of Results
1	(extracorporeal shockwave therapy OR shockwave therapy OR ESWT OR SWT) AND (hand condition OR hand conditions OR hand disease OR hand disease OR hand surgery OR hand operation OR hand)	6
2	(Extracorporeal Shockwave Therapy OR shock wave OR ESWT OR SWT) AND (overview OR review OR conservative OR conservative treatment OR non-surgical)	9
3	(extracorporeal shockwave therapy OR shock wave OR ESWT OR SWT) AND (carpal tunnel syndrome OR CTS OR nerve compression syndrome OR compression neuropathy OR carpal canal syndrome OR thenar of carpal origin OR amyotrophy carpal origin OR distal median nerve compression OR distal median nerve entrapment OR entrapment neuropathy OR median neuropathy)	0
4	(extracorporeal shockwave therapy OR shock wave OR ESWT OR SWT) AND (Trigger finger OR digital stenosing tenosynovitis OR stenosing tenosynovitis OR trigger thumb OR tendovaginitis stenosans OR digital tendovaginitis stenosans OR digitus saltans OR digital flexor tenosynovitis OR tendovaginitis finger OR tenosynovitis stenosans OR stenosing tendovaginitis OR tenosynovitis finger OR trigger digit OR locked finger)	0
5	(extracorporeal shockwave therapy OR shock wave OR ESWT OR SWT) AND (Dupuytren's Disease OR Dupuytren Disease OR Dupytren's contracture OR Dupuytren contracture OR Morbus Dupuytren OR contraction of palmar fascia OR familial palmar fibromatosis OR palmar fascial fibromatosis OR palmar fibromatosis OR Viking's Disease OR Viking Disease OR Celtic hand)	0
6	(extracorporeal shockwave therapy OR shock wave OR ESWT OR SWT) AND (DeQuervain's Syndrome OR DeQuervain's Disease OR DeQuervain's tendinopathy OR DeQuervain's tenosynovitis OR DeQuervain Syndrome OR DeQuervain Disease OR DeQuervain tendinopathy OR DeQuervain tenosynovitis OR De Quervain Syndrome OR De Quervain disease OR De Quervain tendinopathy OR De Quervain tenosynovitis OR Black berry thumb OR texting thumb OR gamer's thumb OR gamer thumb OR radial styloid tenosynovitis OR washerman's sprain OR washerwoman sprain OR mother's wrist OR mother wrist OR mommy thumb OR designer's thumb OR designer thumb OR DeQuervain's tendinitis OR DeQuervain tendinitis OR De Quervain tendinitis)	0
7	(extracorporeal shockwave therapy OR shock wave OR ESWT OR SWT) AND (Kienböck's disease OR Kienböck disease OR Kienbock disease OR Kienbock OR Kienböck OR osteonecrosis of lunate OR osteonecrosis lunate bone OR osteonecrosis carpal lunate OR avascular necrosis lunate OR aseptic necrosis lunate OR lunatomalacia OR osteochondrisis lunate OR progressive avascular necrosis lunate OR progressive aseptic necrosis lunate OR progressive osteonecrosis lunate)	0

Search	Query	Number of Results
	**Total**	15

BMC Musculoskeletal Disorders (BioMed Central Ltd): September 4, 2024

Search	Query	Number of Results
1	(extracorporeal shockwave therapy OR shockwave therapy OR ESWT OR SWT) AND (hand condition OR hand conditions OR hand disease OR hand disease OR hand surgery OR hand operation OR hand)	22
2	(Extracorporeal Shockwave Therapy OR shock wave OR ESWT OR SWT) AND (overview OR review OR conservative OR conservative treatment OR non-surgical)	26
3	(extracorporeal shockwave therapy OR shock wave OR ESWT OR SWT) AND (carpal tunnel syndrome OR CTS OR nerve compression syndrome OR compression neuropathy OR carpal canal syndrome OR thenar of carpal origin OR amyotrophy carpal origin OR distal median nerve compression OR distal median nerve entrapment OR entrapment neuropathy OR median neuropathy)	0
4	(extracorporeal shockwave therapy OR shock wave OR ESWT OR SWT) AND (Trigger finger OR digital stenosing tenosynovitis OR stenosing tenosynovitis OR trigger thumb OR tendovaginitis stenosans OR digital tendovaginitis stenosans OR digitus saltans OR digital flexor tenosynovitis OR tendovaginitis finger OR tenosynovitis stenosans OR stenosing tendovaginitis OR tenosynovitis finger OR trigger digit OR locked finger)	0
5	(extracorporeal shockwave therapy OR shock wave OR ESWT OR SWT) AND (Dupuytren’s Disease OR Dupuytren Disease OR Dupytren’s contracture OR Dupuytren contracture OR Morbus Dupuytren OR contraction of palmar fascia OR familial palmar fibromatosis OR palmar fascial fibromatosis OR palmar fibromatosis OR Viking’s Disease OR Viking Disease OR Celtic hand)	0
6	(extracorporeal shockwave therapy OR shock wave OR ESWT OR SWT) AND (DeQuervain’s Syndrome OR DeQuervain’s Disease OR DeQuervain’s tendinopathy OR DeQuervain’s tenosynovitis OR DeQuervain Syndrome OR DeQuervain Disease OR DeQuervain tendinopathy OR DeQuervain tenosynovitis OR De Quervain Syndrome OR De Quervain disease OR De Quervain tendinopathy OR De Quervain tenosynovitis OR Black berry thumb OR texting thumb OR gamer’s thumb OR gamer thumb OR radial styloid tenosynovitis OR washerman’s sprain OR washerwoman sprain OR mother’s wrist OR mother wrist OR mommy thumb OR designer’s thumb OR designer thumb OR DeQuervain’s tendinitis OR DeQuervain tendinitis OR De Quervain tendinitis)	0
7	(extracorporeal shockwave therapy OR shock wave OR ESWT OR SWT) AND (Kienböck’s disease OR Kienböck disease OR Kienbock disease OR Kienbock OR Kienböck OR osteonecrosis of lunate OR osteonecrosis lunate bone OR osteonecrosis carpal lunate OR avascular necrosis lunate OR aseptic necrosis lunate OR lunatomalacia OR osteochondrisis lunate OR progressive avascular necrosis lunate OR progressive aseptic necrosis lunate OR progressive osteonecrosis lunate)	0
	**Total**	48

Journal of Orthopaedic Surgery and Research (BioMed Central Ltd): September 4, 2024

Search	Query	Number of Results
1	(extracorporeal shockwave therapy OR shockwave therapy OR ESWT OR SWT) AND (hand condition OR hand conditions OR hand disease OR hand disease OR hand surgery OR hand operation OR hand)	10
2	(Extracorporeal Shockwave Therapy OR shock wave OR ESWT OR SWT) AND (overview OR review OR conservative OR conservative treatment OR non-surgical)	20
3	(extracorporeal shockwave therapy OR shock wave OR ESWT OR SWT) AND (carpal tunnel syndrome OR CTS OR nerve compression syndrome OR compression neuropathy OR carpal canal syndrome OR thenar of carpal origin OR amyotrophy carpal origin OR distal median nerve compression OR distal median nerve entrapment OR entrapment neuropathy OR median neuropathy)	0
4	(extracorporeal shockwave therapy OR shock wave OR ESWT OR SWT) AND (Trigger finger OR digital stenosing tenosynovitis OR stenosing tenosynovitis OR trigger thumb OR tendovaginitis stenosans OR digital tendovaginitis stenosans OR digitus saltans OR digital flexor tenosynovitis OR tendovaginitis finger OR tenosynovitis stenosans OR stenosing tendovaginitis OR tenosynovitis finger OR trigger digit OR locked finger)	0
5	(extracorporeal shockwave therapy OR shock wave OR ESWT OR SWT) AND (Dupuytren's Disease OR Dupuytren Disease OR Dupytren's contracture OR Dupuytren contracture OR Morbus Dupuytren OR contraction of palmar fascia OR familial palmar fibromatosis OR palmar fascial fibromatosis OR palmar fibromatosis OR Viking's Disease OR Viking Disease OR Celtic hand)	0
6	(extracorporeal shockwave therapy OR shock wave OR ESWT OR SWT) AND (DeQuervain's Syndrome OR DeQuervain's Disease OR DeQuervain's tendinopathy OR DeQuervain's tenosynovitis OR DeQuervain Syndrome OR DeQuervain Disease OR DeQuervain tendinopathy OR DeQuervain tenosynovitis OR De Quervain Syndrome OR De Quervain disease OR De Quervain tendinopathy OR De Quervain tenosynovitis OR Black berry thumb OR texting thumb OR gamer's thumb OR gamer thumb OR radial styloid tenosynovitis OR washerman's sprain OR washerwoman sprain OR mother's wrist OR mother wrist OR mommy thumb OR designer's thumb OR designer thumb OR DeQuervain's tendinitis OR DeQuervain tendinitis OR De Quervain tendinitis)	0
7	(extracorporeal shockwave therapy OR shock wave OR ESWT OR SWT) AND (Kienböck's disease OR Kienböck disease OR Kienbock disease OR Kienbock OR Kienböck OR osteonecrosis of lunate OR osteonecrosis lunate bone OR osteonecrosis carpal lunate OR avascular necrosis lunate OR aseptic necrosis lunate OR lunatomalacia OR osteochondrisis lunate OR progressive avascular necrosis lunate OR progressive aseptic necrosis lunate OR progressive osteonecrosis lunate)	0
	**Total**	**30**

Registry/Website Searches

Website Name: Website URL (Date Searched)	Query
Cochrane https://training.cochrane.org/handbook/current/chapter-08 (September 4, 2024)	Assessing risk of bias in a randomized trial
Cochrane https://training.cochrane.org/handbook/current/chapter-25 (September 4, 2024)	Assessing risk of bias in a non-randomized study
American Society of Plastic Surgeons (ASPS) https://www.plasticsurgery.org/documents/medical-professionals/health-policy/evidence-practice/ASPS-Rating-Scale-March-2011.pdf (September 4, 2024)	Evidence Rating Scale for Therapeutic Studies from the American Society of Plastic Surgeons
American Society of Plastic Surgeons (ASPS) https://www.plasticsurgery.org/documents/medical-professionals/health-policy/evidence-practice/ASPS-Scale-for-Grading-Recommendations.pdf (September 4, 2024)	Scale for Grading Recommendations from the American Society of Plastic Surgeons
Jones, E.P. & Cawley, M. (2022). *Search Strategy Documentation Template.* University of North Carolina at Chapel Hill (UNC) Health Sciences Library. https://guides.lib.unc.edu/systematic-reviews/search (September 4, 2024)	Search Strategy Documentation

Forward and Backward Reference Searching

Citation	Date Searched	Source / Method*	Number of Results	
			Forward	Backward
Haghighat S, Vahdatpour B, Ataei E . The Effect of Extracorporeal Shockwave Therapy on de Quervain Tenosynovitis; a Clinical Trial. Shiraz E-Med J. 2021;22(8):e106559. https://doi.org/10.5812/semj.106559.	September 4, 2024	Google Scholar	0	4
Gesslbauer C, Mickel M, Schuhfried O, Huber D, Keilani M, Crevenna R. Effectiveness of focused extracorporeal shock wave therapy in the treatment of carpal tunnel syndrome: A randomized, placebo-controlled pilot study. Wien Klin Wochenschr. 2021 Jun;133(11-12):568-577. doi: 10.1007/s00508-020-01785-9. Epub 2020 Dec 22. PMID: 33351153; PMCID: PMC7754699.	September 4, 2024	PubMed	1	6
Fernando JJ, Fowler C, Graham T, Terry K, Grocott P, Sandford F. Pre-operative hand therapy management of Dupuytren's disease: A systematic review. Hand Ther. 2024 Jun;29(2):52-61. doi: 10.1177/17589983241227162. Epub 2024 Jan 28. PMID: 38827652; PMCID: PMC11143942.	September 17, 2024	PubMed	0	1
**Total Results from Forward and Backward Reference Searches**			1	11

*Forward searching refers to list of publications that have referenced the citation since its publication. Backward searching refers to the references included in the citation's bibliography. Various databases can be used to execute forward citation searches including Scopus, Google Scholar, or PubMed Central. The number of forward citations identified per reference will vary by database, so it is important to specify the source used. Backward citation searching can be completed by analyzing an article's reference list or by using a database such as Scopus. This guide from University of Wisconsin Libraries provides further explanation on forward and backward searching.

Subject Matter Experts Contacted

Expert Name and Affiliation	Contact Information	Date Contacted	Number of Results
Prof. Dr. med. Andreas Jokuszies (Hannover Medical School)	Jokuszies.Andreas@mh-hannover.de	September 11, 2023	56
		**Total Results Provided by Subject Matter Experts**	**56**

Additional Notes

**1.**	**Indicate process for removing duplicates.**	Results were de-duplicated using RefWorks.
**2.**	**List filters or limits applied.**	Language = English<br>Publication Date: from start of database to present
**3.**	**Was this search based on a previous search such as one published in a prior review? (If yes, provide citation or source.)**	No.
**4.**	**Was this search peer-reviewed? If so, by whom and what are their qualifications and area of expertise?**	No.
**5.**	**Other Notes**	NA

Excluded studies:

Authors	Explanation
[1]	SR- Incomplete study
[2]	RCT - ESWT as a post-surgical measure
[3]	RCT - ESWT as a post-surgical measure
[4]	"overview" – not enough RCTs included (1)
[5]	Letter
[6]	Case report
[7]	Hypothesis
[8]	SR – not enough RCTs (1)
[9]	Narrative review
[10]	SR - Conditions of interest are not mentioned
[11]	SR – not enough RCTs (1)
[12]	RCT- no mention of ESWT
[13]	SR – not enough RCTs (1)
[14]	RCT – no mention of ESWT
[15]	Dissertation – incomplete
[16]	Review – Conditions of interest are not mentioned
[17]	Comprehensive review – not enough RCTs (2)

Authors	Explanation
[18]	Literature review
[19]	Narrative review
[20]	SR – not enough RCTs mentioned (1)
[21]	SR – not enough RCTs mentioned (0)
[22]	SR – not enough RCTs mentioned (0)
[23]	Review – not enough RCTs mentioned (0)
[24]	Letter
[25]	Letter
[26]	Letter
[27]	SR – not enough RCTs mentioned (1)
[28]	SR – Conditions of interest are not mentioned
[29]	Narrative review
[30]	Review – Conditions of interest are not mentioned
[31]	Review – ESWT as a post-surgical measure
[32]	Review – not enough RCTs mentioned (0)
[33]	Review - ESWT as a post-surgical measure
[34]	Review – Conditions of interest are not mentioned
[35]	Review – not enough RCTs mentioned (0)
[36]	Review – Conditions of interest are not mentioned
[37]	Review – Conditions of interest are not mentioned
[38]	Full-text not available
[39]	Full-text not available
[40]	Unclear method and documentation (incomplete)
[41]	No meta-analysis
[13]	No meta-analysis
[42]	No meta-analysis
[43]	Umbrella review

(1) Karjalanen T, Raatikainen S, Jaatinen K, Lusa V. Update on Efficacy of Conservative Treatments for Carpal Tunnel Syndrome. J Clin Med 2022 -02-11;11(4):950.

(2) Haghighat S, Zarezadeh A, Khosrawi S, Oreizi A. Extracorporeal Shockwave Therapy in Pillar Pain after Carpal Tunnel Release: A Prospective Randomized Controlled Trial. Adv Biomed Res 2019;8:31.

(3) Turgut MC, Saglam G, Toy S. Efficacy of extracorporeal shock wave therapy for pillar pain after open carpal tunnel release: a double-blind, randomized, sham-controlled study. Korean J Pain 2021 -07-01;34(3):315–321.

(4) Matthews A, Smith K, Read L, Nicholas J, Schmidt E. Trigger finger: An overview of the treatment options. JAAPA 2019 -01;32(1):17–21.

(5) Chen C, Zeng Z. Letter to the editor regarding "A randomized controlled trial: comparing extracorporeal shock wave therapy versus local corticosteroid injection for the treatment of carpal tunnel syndrome". Med Oncol 2020 -09-22;37(10):92.

(6) Brunelli S, Bonanni C, Traballesi M, Foti C. Radial extracorporeal shock wave therapy: a novel approach for the treatment of Dupuytren's contractures: A case report. Medicine (Baltimore) 2020 -06-12;99(24):e20587.

(7) Knobloch K, Kuehn M, Vogt PM. Focused extracorporeal shockwave therapy in Dupuytren's disease--a hypothesis. Med Hypotheses 2011 -05;76(5):635–637.

(8) R M, S H, W K. Recent Surgical and Medical Advances in the Treatment of Dupuytren's Disease - A Systematic Review of the Literature. Open Orthop J 2012;6:77–82.

(9) De la Corte-Rodríguez H, Román-Belmonte JM, Rodríguez-Damiani BA, Vázquez-Sasot A, Rodríguez-Merchán EC. Extracorporeal Shock Wave Therapy for the Treatment of Musculoskeletal Pain: A Narrative Review. Healthcare (Basel) 2023 -10-26;11(21):2830.

(10) Auersperg V, Trieb K. Extracorporeal shock wave therapy: an update. EFORT Open Rev 2020 -10;5(10):584–592.

(11) Häußer J, Wieber J, Catalá-Lehnen P. The use of extracorporeal shock wave therapy for the treatment of bone marrow oedema - a systematic review and meta-analysis. J Orthop Surg Res 2021 -06-09;16(1):369.

(12) Sharma R, Aggarwal AN, Bhatt S, Kumar S, Bhargava SK. Outcome of low level lasers versus ultrasonic therapy in de Quervain's tenosynovitis. Indian J Orthop 2015;49(5):542–548.

(13) Chong HH, Pradhan A, Dhingra M, Liong W, Hau MYT, Shah R. Advancements in de Quervain Tenosynovitis Management: A Comprehensive Network Meta-Analysis. J Hand Surg Am 2024 -06;49(6):557–569.

(14) Suwannaphisit S, Suwanno P, Fongsri W, Chuaychoosakoon C. Comparison of the effect of ketorolac versus triamcinolone acetonide injections for the treatment of de Quervain's tenosynovitis: a double-blind randomized controlled trial. BMC Musculoskelet Disord 2022 -09-01;23(1):831.

(15) Seely A. The Efficacy of Extracorporeal Shock Wave Therapy in Comparison to Low-level Laser Therapy for Decreasing Pain and Symptom Severity and Increasing Functional Status in Adult Patients with Carpal Tunnel Syndrome: A Systematic Review and Meta-analysis. : State University, Fresno; 2021.

(16) Poenaru D, Ojoga F, Sandulescu M, Cinteza D. Conservative therapy in ulnar neuropathy at the elbow (Review). Exp Ther Med 2022 -08;24(2):517.

(17) Urits I, Gress K, Charipova K, Orhurhu V, Kaye AD, Viswanath O. Recent Advances in the Understanding and Management of Carpal Tunnel Syndrome: a Comprehensive Review. Curr Pain Headache Rep 2019 -08-01;23(10):70.

(18) Wielemborek PT, Kapica-Topczewska K, Pogorzelski R, Bartoszuk A, Kochanowicz J, Kułakowska A. Carpal tunnel syndrome conservative treatment: a literature review. Postep Psychiatr Neurol 2022 -06;31(2):85–94.

(19) Ferrara PE, Codazza S, Maccauro G, Zirio G, Ferriero G, Ronconi G. Physical therapies for the conservative treatment of the trigger finger: a narrative review. Orthop Rev (Pavia) 2020 June 26;12(Suppl 1):8680.

(20) Fleckenstein J, Banzer W. A review of hands-on based conservative treatments on pain in recreational and elite athletes. Science & Sports 2019;34(2):e77–e100.

(21) Khan M, Shanmugaraj A, Prada C, Patel A, Babins E, Bhandari M. The Role of Hyaluronic Acid for Soft Tissue Indications: A Systematic
Review and Meta-Analysis. Sports Health 2022 February 3;15(1):86–96.

(22) Valen PA, Foxworth J. Evidence supporting the use of physical modalities in the treatment of upper extremity musculoskeletal conditions. Curr Opin Rheumatol 2010 -03;22(2):194–204.

(23) Al-Zamil M, Minenko IA, Kulikova NG, Mansur N, Nuvakhova MB, Khripunova OV, et al. Efficiency of Direct Transcutaneous Electroneurostimulation of the Median Nerve in the Regression of Residual Neurological Symptoms after Carpal Tunnel Decompression Surgery. Biomedicines 2023 -08-27;11(9):2396.

(24) Xue H, Zhang B, Rong J. Effect of extracorporeal shock wave therapy on carpal tunnel syndrome based on medical big data. Minerva Surg 2024 -02;79(1):115–117.

(25) Chang K, Hung C, Özçakar L. Snapping Thumb and Superficial Radial Nerve Entrapment in De Quervain Disease: Ultrasound Imaging/Guidance Revisited. Pain Med 2015 -11;16(11):2214–2215.

(26) Ogihara H, Ariie T, Tsujimoto Y. Major Concerns Regarding the Study of Extracorporeal Shockwave Therapy in the Treatment of Trigger Finger. Arch Phys Med Rehabil 2022 -06;103(6):1240.

(27) Shen P, Chou S, Lu C, Fu Y, Lu C, Liu W, et al. Comparative effectiveness of various treatment strategies for trigger finger by pairwise meta-analysis. Clin Rehabil 2020 -09;34(9):1217–1229.

(28) Wigley CH, Janssen TJ, Mosahebi A. Shock Wave Therapy in Plastic Surgery: A Review of the Current Indications. Aesthet Surg J 2023 -02-21;43(3):370–386.

(29) Steere HK, DeLuca S, Borg-Stein J, Malanga GA, Tenforde AS. A Narrative Review Evaluating Extracorporeal Shockwave Therapy as a Potential Regenerative Treatment for Musculoskeletal Conditions in Military Personnel. Mil Med 2021 -07-01;186(7-8):682–706.

(30) Bratsun D, Mizev A, Mosheva E, Kostarev K. Shock-wave-like structures induced by an exothermic neutralization reaction in miscible fluids. Phys Rev E 2017 -11;96(5-1):053106.

(31) Sprangers PN, Westenberg RF, Langer MF, Oflazoglu K, van der Heijden EPA. State of the art review. Complications after carpal tunnel release. J Hand Surg Eur Vol 2024 -02;49(2):201–214.

(32) Huang HH, Qureshi AA, Biundo JJ. Sports and other soft tissue injuries, tendinitis, bursitis, and occupation-related syndromes. Curr Opin Rheumatol 2000 -03;12(2):150–154.

(33) Ambroziak M. Application of ESWT in post-operative treatment in Carpal Tunnel Syndrome - a review. Pol Przegl Chir 2020 -04-16;92(3):39–43.

(34) Frizziero A, Trainito S, Oliva F, Nicoli Aldini N, Masiero S, Maffulli N. The role of eccentric exercise in sport injuries rehabilitation. Br Med Bull 2014 -06;110(1):47–75.

(35) Tettenborn B, Mehnert S, Reuter I. [Peripheral Nerve Injuries in Sports]. Fortschr Neurol Psychiatr 2016 -09;84(9):551–567.

(36) Schroeder AN, Tenforde AS, Jelsing EJ. Extracorporeal Shockwave Therapy in the Management of Sports Medicine Injuries. Curr Sports Med Rep 2021 -06-01;20(6):298–305.

(37) Romeo P, Lavanga V, Pagani D, Sansone V. Extracorporeal Shock Wave Therapy in Musculoskeletal Disorders: A Review. Medical Principles and Practice 2013 November 5;23(1):7–13.

(38) Elerian A. EFFECT OF SHOCKWAVES VERSUS TRADITIONAL PHYSIOTHERAPY IN TREATING DE QUERVAIN TENOSYNOVITIS. 2016.

(39) Notarnicola A, Maccagnano G, Rifino F, Pesce V, Gallone MF, Covelli I, et al. Short-term effect of shockwave therapy, temperature controlled high energy adjustable multi-mode emission laser or stretching in Dupuytren's disease: a prospective randomized clinical trial. J Biol Regul Homeost Agents 2017;31(3):775–784.

(40) Aykut S, Aydın C, Öztürk K, Arslanoğlu F, Kılınç CY. Extracorporeal Shock Wave Therapy in Dupuytren's Disease. Sisli Etfal Hastan Tip Bul 2018;52(2):124–128.

(41) Yazdani A, Nasri P, Baradaran Mahdavi S. The Effects of Shock Wave Therapy on the Symptoms and Function of Individuals With Dupuytren Disease: A Systematic Review. Arch Phys Med Rehabil 2024 -06-10:S0003–5.

(42) Huisstede BMA, Coert JH, Fridén J, Hoogvliet P. Consensus on a multidisciplinary treatment guideline for de Quervain disease: results from the European HANDGUIDE study. Phys Ther 2014 -08;94(8):1095–1110.

(43) Dabbagh A, Ziebart C, MacDermid JC, Packham T, Grewal R. The effectiveness of biophysical agents in the treatment of carpal tunnel syndrome- an umbrella review. BMC Musculoskelet Disord 2023 -08-10;24(1):645.
