# Supplementary material for: State of shock – a systematic review of extracorporeal shockwave therapy in hand surgery
Source: GMS Interdiscip Plast Reconstr Surg DGPW. 2025 Dec 22;14:Doc03. doi: 10.3205/iprs000192 (PMC12833726; doi:10.3205/iprs000192)
Supplement: Supplementary tables [file IPRS-14-03-s-002.pdf]

Attachment 2: Supplementary tables

Level of evidence	Qualifying studies
I	High-quality, multi-centered or single-centered, randomized controlled trial with adequate power; or systematic review of these studies
II	Lesser quality, randomized controlled trial; prospective cohort or comparative study; or systematic review of these studies
III	Retrospective cohort or comparative study; case-control study; or systematic review of these studies
IV	Case series with pre/post-test; or only post test
V	Expert opinion developed via consensus process; case report or clinical example; or evidence based on physiology, bench research or “first principles”

Table S1: Evidence Rating Scale for Therapeutic Studies by the American Society of Plastic Surgeons

Grade	Descriptor	Qualifying evidence	Implications for practice
A	Strong recommendation	Level I evidence or consistent findings from multiple studies of levels II, III, or IV	Clinicians should follow a strong recommendation unless a clear and compelling rationale for an alternative approach is present.
B	Recommendation	Levels II, III, or IV evidence and findings are generally consistent	Generally, clinicians should follow a recommendation but should remain alert to new information and sensitive to patient preferences.
C	Option	Levels II, III, or IV evidence, but findings are inconsistent	Clinicians should be flexible in their decision making regarding appropriate practice, although they may set bounds on alternatives; patient preference should have a substantial influencing role.
D	Option	Level V: Little or no systematic empirical evidence	Clinicians should consider all options in their decision-making and be alert to new published evidence that clarifies the balance of benefit versus harm; patient preference should have a substantial influencing role.

Table S2: Scale for Grading Recommendations by the American Society of Plastic Surgeons

**Condition**	**Study designs**	**Number of studies**	**Total number of participants**
Dupuytren’s disease	RCT	2	ca. 223
	PPS	1	
	CS	1	
	SR	1	
Trigger finger	RCT	2	208
	PPS	4	
DeQuervain’s tenosynovitis	RCT	2	56
Osteonecrosis of the lunate	PPS	1	22
Carpal tunnel syndrome	RCT	22	6,814
	CS	1	
	SR	7	
**Total**	**-**	**44**	**7,323**

RTC = randomized-controlled trial, PPS = pre-post study, CS = case series, SR = systematic review

Table S3: Overview of the included studies

Authors (Country, year)	Study design	Groups	No. of participants (No. of fingers)	Age [years] (SD)	Sex [F/M]	ESWT protocol					Follow-up	Primary outcome (secondary outcome)
						Type of ESWT	Pressure [bar] or energy flux density [mJ/mm²]	Frequency [Hz]	No. of shocks	No. of sessions		
Knobloch et al. (Germany, 2022)	RCT	ESWT<br>Sham ESWT	27 (NA)<br>25 (NA)	57.6 (8.1)<br>58.9 (10.9)	12/15<br>8/17	fESWT	0.35 mJ/mm²	3 Hz	2,000	3/3 weeks	Pret. baseline, month 3, 6, 12, 18 postt.	Pain VAS (DASH, MHQ, URAM, hand-grip strength)
Saad et al. (Egypt, 2021)	RCT	ESWT + Splinting + Stretching<br>LIPUS + Splinting + Physiotherapy	15 (NA)<br>15 (NA)	55.4 (4.91)<br>55 (5.61)	1/14<br>2/13	NA	0.0018 mJ/mm²	6-8 Hz	1,700	6/6 weeks	Pret. baseline, week 6 postt.	qDASH, hand-grip strength
Abdulsalam et al. (Kuwait, 2019)	Case series	ESWT	4 (7)	59.75 (NA)	2/2	fESWT	1.24 mJ/mm²	3 Hz	2,000	5/5 weeks	Pret. baseline, week 8 postt.	MHQ (for pain & tenderness), hand-grip strength
Taheri et al. (Iran, 2022)	Pre-post study	ESWT	20 (32)	66.6 (7.11)	9/11	fESWT	1.24 mJ/mm²	3 Hz	2,000	6/6 weeks	Pret. baseline, week 6, 14 postt.	Pain VAS, DASH, MCP contraction angle
Fernando et al. (UK, 2024)	SR	ESWT vs. Stretching, LCI, Splinting, US, CFM	17 studies	NA	NA	fESWT + rESWT	NA	NA	NA	NA	Multiple and various time points	Pain VAS, ROM, hand-grip strength, R&Ms

RCT = randomized-controlled trial; SR = systematic review; MA = meta-analysis; SD = standard deviation; ESWT = extracorporeal shockwave therapy; fESWT = focused extracorporeal shockwave therapy; rESWT = radial extracorporeal shockwave therapy; LCI = local corticosteroid injection; US = ultrasonography; LIPUS = low-intensity pulsed ultrasound; CFM = cross-frictional massage; Pret. baseline = pre-treatment baseline; postt. = post-treatment; VAS = visual analog scale; DASH = Disabilities of the arm, shoulder, and hand score; qDASH = quick DASH; SF-36 = 36-item Short Form Health Survey Questionnaire; ROM = Range of Motion; MHQ = Michigan Health Questionnaire; URAM = Unité Rhumatologique des Affections de la Main; MCP contraction angle = metacarpophalangeal contraction angle; R&Ms = Roles and Maudsley score; NA = not available.

Table S4: Dupuytren’s disease study characteristics

Authors (Country, year)	Study design	Groups	No. of participants (No. of fingers)	Age [years] (SD)	Sex [F/M]	ESWT protocol					Follow-up	Primary outcome (secondary outcome)
						Type of ESWT	Pressure [bar] or energy flux density [mJ/mm²]	Frequency [Hz]	No. of shocks	No. of sessions		
Vahdatpour et al. (Iran, 2020)	Pre-post study	ESWT	18 (18)	NA	12/6	rESWT (peripheral tissue of the nodule)<br>fESWT (directly on the nodule)	2.1 bar<br>0.1 bar	15 Hz<br>4 Hz	1,000<br>500	3/3 weeks<br>3/3 weeks	Pret. baseline, week 0, 6, 18 postt.	Pain VAS, Triggering VAS, Function VAS, Trigger Finger Score by Quinnell, DASH
Malliaro-poulos et al. (Greece, 2016)	Pre-post study	ESWT	44 (49)	49.06 (9.99)	29/15	rESWT	1-3 bar	5-6 Hz	2,000	1/1 week as long as symptoms persisted	Pret. baseline, month 1, 3, 12 postt.	Pain VAS, R&Ms
Chen et al. (Taiwan, 2021)	RCT	High-energy ESWT<br>Low-energy ESWT<br>Sham ESWT	18 (18)<br>19 (19)<br>19 (19)	56.2 (8.9)<br>55.6 (7.3)<br>54.8 (13.4)	13/5<br>13/6<br>15/4	fESWT	5.8 bar, 0.01mJ/mm²<br>3 bar, 0.006 mJ/mm²	NA<br>NA	1,500<br>1,500	4/4 weeks	Pret. baseline, month 1, 3, 6 postt.	Pain VAS, TFA, qDASH
Yildirim et al. (Turkey, 2016)	RCT	ESWT<br>LCI (0.5 mL betamethasone diproionate/ sodium phosphate + 2% lidocaine)	20 (20)<br>20 (20)	55 (8)<br>54 (9)	16/4<br>17/3	NA	2.1 bar	15 Hz	1,000	3/3 weeks	Pret. baseline, month 1, 3, 6 postt.	Pain VAS, TFA, qDASH
Zyluk and Mosiejczuk (Poland, 2020)	Pre-post study	ESWT	32 (50)	60 (NA)	28/4	NA	2 bar	8 Hz	2,000	3/3 weeks	Pret. baseline, week 1, month 3 postt.	Pain NRS, Triggering on Froimson scale
Dogru et al. (Turkey, 2020)	Pre-post study	ESWT	18 (20)	59 (NA)	15/3	rESWT	2 bar	10 Hz	2,000	10/5 weeks	Pret. baseline, 0, 3 months post.	Pain NRS, qDASH, hand-grip, pinch strength, ROM

RCT = randomized-controlled trial; SD = standard deviation; ESWT = extracorporeal shockwave therapy; fESWT = focused extracorporeal shockwave therapy; rESWT = radial extracorporeal shockwave therapy; LCI = local corticosteroid injection; Pret. baseline = pre-treatment baseline; postt. = post-treatment; VAS = visual analog scale; NRS = numeric rating scale; DASH = disabilities of the arm, shoulder, and hand score; qDASH = quick DASH; ROM = range of motion; R&Ms = Roles and Maudsley score; TFA = trigger finger assessment; NA = not available.

Table S5: Trigger finger study characteristics

Authors (Country, Year)	Study design	Groups	No. of participants (No. of hands)	Age [years] (SD)	Sex [F/M]	ESWT protocol					Follow-up	Primary outcome (secondary outcome)
						Type of ESWT	Pressure [bar] or energy flux density [mJ/mm²]	Frequency [Hz]	No. of shocks	No. of sessions		
Haghighat et al. (Iran, 2020)	RCT	ESWT + Splinting + Celecoxib<br>Sham ESWT + Splinting + Celecoxib	13 (13)<br>13 (13)	44.61 (11.36)<br>48.23 (14.45)	7/6<br>9/4	NA	3 bar	2 Hz	1,000	3/3 weeks	Pret. baseline, week 0, 3, 6 postt.	DASH, Pain VAS, hand-grip strength
Notarnicola et al. (Italy, 2022)	RCT	ESWT<br>LCI (1 mL methylprednisolone 40mg + 2% lidocaine)	15 (15)<br>15 (15)	56.4 (11.5)	26/4	fESWT	0.03 -0.14 mJ/mm² (according to patient's tolerance)	4 Hz	1,600	3/3 weeks	Pret. baseline, 3, 6 months postt.	Ritchie's tenderness scale, hand-grip strength, Pain VAS, DASH, SF-36

RCT = Randomized-Controlled Trial; SD = standard deviation; ESWT = Extracorporeal Shockwave Therapy; fESWT = focused Extracorporeal Shockwave Therapy; LCI = Local Corticosteroid Injection; Pret. baseline = pre-treatment baseline; postt. = post-treatment; VAS = Visual Analog Scale; DASH = Disabilities of the arm, shoulder, and hand score; SF-36 = 36-item Short Form Health Survey Questionnaire; NA = not available.

Table S6: De Quervain's tenosynovitis study characteristics

Authors (Country, Year)	Study design	Groups	No. of participants (No. of wrists)	Age [years] (SD)	Sex [F/M]	ESWT-Protocol					Follow-up	Primary outcome (secondary outcome)
						Type of ESWT	Pressure [bar] or energy flux density [mJ/mm²]	Frequency [Hz]	No. of shocks	No. of sessions		
D'Agostino et al. (Italy, 2011)	Pre-post-study	ESWT + Splinting	22 (22)	37.4 (NA)	6/16	NA	0.35-0.4 mJ/mm²	4 Hz	4,000	3/3 months	Pret. baseline, month 0, 2, 6, year 1 postt.	Pain VAS, ROM, bone edema

SD = standard deviation; ESWT = extracorporeal shockwave therapy; Pret. baseline = pre-treatment baseline; postt. = post-treatment; VAS = visual analog scale; ROM = range of motion; NA = not available.

Table S7: Osteonecrosis of the lunate study characteristics

Authors (Country, Year)	Study design	Groups	No. of participants (No. of wrists)	Age [years] (SD)	Sex [F/M]	ESWT-Protocol					Follow-up	Primary outcome (secondary outcome)
						Type of ESWT	Pressure [bar] or energy flux density [mJ/mm²]	Frequency [Hz]	No. of shocks	No. of sessions		
Vahdatpour et al. (Iran, 2015)	RCT	ESWT + Splinting<br>Sham ESWT + Splinting	28 (30)<br>27 (30)	51.5 (8.5)<br>49 (7.3)	51/9	fESWT	0.05–0.15 mJ/mm²	3 Hz	800–1,100	4/4 weeks	Pret. baseline, month 3, 6 postt.	Pain VAS, BCTQ, electrodiagnostic studies
Atthakomol et al. (Thailand, 2018)	RCT	ESWT<br>LCI (1mL triamcinolone acetonide 10mg + 1% lidocaine)	13 (13)<br>12 (12)	46 (9)<br>53 (12)	8/2<br>11/1	rESWT	4 Bar	15 Hz	5,000	1/1 week	Pret. baseline, week 1, 4, 12, 24	BCTQ (Pain VAS, electrodiagnostic studies)
Notarnicola et al. (Italy, 2015)	RCT	ESWT<br>Nutraceutical group (mainly ALA + GLA + Echinacea)	34 (34)<br>26 (26)	57.1 (9.5)<br>60.2 (6.6)	NA<br>NA	fESWT	0.03 mJ/mm²	4 Hz	1600	3/5 weeks	Pret. baseline, month 1, 2, 4, 6 postt.	Pain VAS, BCTQ, R&Ms, electrodiagnostic studies
Paoloni et al. (Italy, 2016)	RCT	ESWT<br>US<br>Cryo-US	8 (12)<br>8 (13)<br>9 (17)	59.1 (12.5)<br>56.5 (9.4)<br>54.7 (9.2)	11/1<br>15/2<br>12/1	fESWT	0.05 mJ/mm²	NA	2500	4/4 weeks	Pret. baseline, week 0, 4, 12 postt.	Pain & Paresthesia VAS, BCTQ
Seok and Kim (Italy, 2013)	RCT	ESWT<br>LCI (1mL triamcinolone acetonide 40mg + lidocaine)	15 (18)<br>16 (18)	54.03 (19.47)<br>49.67 (18.83)	12/3<br>14/2	fESWT	0.09-0.29 mJ/mm²	6 Hz	1000	1/1 week	Pret. baseline, month 1, 3 postt.	Pain VAS, BCTQ, electrodiagnostic studies
Raissi et al. (Iran, 2016)	RCT	ESWT + Splinting<br>Splinting	20 (20)<br>20 (20)	46.1 (1.95)<br>46.65 (2.23)	18/2<br>19/1	rESWT	1.5 Bar	6 Hz	1000	3/3 weeks	Pret. baseline, week 3, 8, 12 postt.	Pain VAS (qDASH, electrodiagnostic studies)

Wu et al. (China, 2015)	RCT	ESWT + Splinting<br>Sham ESWT + Splinting	17 (20)<br>17 (20)	54.7 (7.96)<br>57.8 (6.51)	18/2<br>17/3	rESWT	4 Bar	5 Hz	2000	3/3 weeks	Pret. baseline, week 1, 4, 8, 12 postt.	Pain VAS (BCTQ, Finger pinch strength, electrodiagnostic studies)
Ke et al. (China, 2016)	RCT	ESWT + Splinting (3 sessions)<br>ESWT + Splinting (1 session)<br>Sham ESWT + Splinting	23 (30)<br>22 (29)<br>23 (30)	56.33 (1.48)<br>55.45 (1.38)<br>58.13 (1.13)	24/6<br>23/6<br>25/5	rESWT	4 Bar	5 Hz	2000	3/3 weeks	Pret. baseline, week 2, 8, 12 postt.	BCTQ (electrodiagnostic studies)
Xu et al. (China, 2019)	RCT	ESWT + Splinting<br>LCI + Splinting (1 mL betamethason e 40mg + lidocaine)	30 (30)<br>25 (25)	47.2 (1.86)<br>46.9 (1.76)	25/5<br>21/4	rESWT	1.5 Bar	6 Hz	1000	9/3 weeks	Pret. baseline, week 3, 9, 12 postt.	Pain VAS, BCTQ, electrodiagnostic studies
Chang et al. (China, 2019)	RCT	ESWT + PRP<br>Sham ESWT + PRP	20 (32)<br>20 (32)	56.47 (6.31)<br>58.63 (7.69)	29/3<br>30/2	rESWT	4 Bar	5 Hz	2000	1/1 week	Pret. baseline, month 1, 3, 6 postt.	BCTQ (electrodiagnostic studies)
Sweilam et al. (Egypt, 2019)	RCT	ESWT<br>LCI (Triamcinolone acetonide 40 mg + lidocaine)	25 (25)<br>28 (28)	37.6 (8.5)<br>36.8 (8.8)	21/4<br>23/5	NA	2 Bar	10 Hz	2500	2/2 week	Pret. baseline, week 2, 4 postt.	Pain VAS, BCTQ, electrodiagnostic studies
Gesslbauer et al. (Austria, 2020)	RCT	ESWT<br>Sham ESWT	10 (NA)<br>10 (NA)	55.8 (4.66)<br>54 (17.4)	8/2<br>6/4	fESWT	0.05 mJ/mm²	4 Hz	500	3/3 weeks	Pret. baseline, week 3, 12 postt.	Pain VAS (electrodiagnostic studies, hand-grip strength, BCTQ, SF-36)
Habibzadeh et al. (Iran, 2022)	RCT	(point) ESWT + physiotherapy<br>(sweep) ESWT + physiotherapy<br>Physiotherapy	20 (NA)<br>20 (NA)<br>20 (NA)	45.4 (11.49)<br>50.55 (11.99)<br>51 (7.77)	18/2<br>18/2<br>15/5	rESWT<br>rESWT	1.5 Bar	6 Hz	1500 (applied on carpal tunnel)<br>1000 (on carpal	4/4 weeks<br>4/4 weeks	Pret. baseline, week 1, 4 postt.	Pain & Paresthesia VAS, electrodiagnostic studies (BCTQ)

									tunnel) & 500 (on median nerve on palmar surface			
Durmaz et al. (Turkey, 2022)	RCT	ESWT<br>LCI<br>Splinting	33 (NA)<br>28 (NA)<br>31 (NA)	51.1 (7.1)<br>54.1 (9.6)<br>50.4 (9.8)	23/10<br>20/8<br>27/4	rESWT	4 Bar	5 Hz	2000	3/3 weeks	Pret. baseline, week 1, 12 postt.	BCTQ (Pain & Numbness VAS, hand-grip strength, electrodiagnostic studies)
Razali et al. (Malaysia, 2022)	RCT	US + Physiotherapy<br>ESWT + Physiotherapy<br>Physiotherapy	20 (30)<br>20 (31)<br>20 (29)	NA<br>NA<br>NA	38/14	NA	2 Bar	3 Hz	1000	8/4 weeks	Pret. baseline, week 2, 4 of treatment, 4 weeks postt.	BCTQ
Saglam et al. (Turkey, 2022)	RCT	Splinting + Exercise<br>ESWT + Exercise<br>Physiotherapy	32 (42)<br>32 (42)<br>32 (42)	55.8 (11.3)<br>53.8 (11.8)<br>53.4 (10.9)	32/10<br>34/8<br>29/12	rESWT	4 Bar	5 Hz	2000	3/3 weeks	Pret. baseline, week 3, 12 postt.	Pain VAS, BCTQ, LANSS, electrodiagnostic studies
Zhang et al. (China, 2023)	RCT	ESWT<br>Nerve mobilization + Splinting	47 (NA)<br>45 (NA)	47.57 (3.67)<br>46.57 (3.24)	32/15<br>29/16	NA	0.16 mJ/mm²	NA	Min. 2000	8/4 weeks	Pret. baseline, week 1, 2 postt.	Pain VAS, BCTQ, ADL, GSS, electrodiagnostic studies
Ghasemi et al. (Iran, 2023)	RCT	ESWT<br>LLLT	12 (18)<br>13 (18)	41.9 (9.7)<br>41.8 (6.6)	15/3<br>16/2	rESWT	4 Bar	15 Hz	900	4/2 weeks	Pret. baseline, week 2 postt.	Pain VAS, CSA
Ulucaköy et al. (Turkey, 2020)	RCT	Splinting<br>Splinting + ESWT<br>ESWT<br>Splinting + Sham ESWT	47 (NA)<br>47 (NA)<br>45 (NA)<br>50 (NA)	48.1 (10.1)<br>48.4 (10.1)<br>50 (8.6)	40/7<br>39/8<br>41/4<br>47/3	rESWT	0.05 mJ/mm²	5 Hz	1000	3/3 weeks	Pret. baseline, month 1, 3 postt.	Pain VAS, BCTQ, LANSS, pinch strength, electrodiagnostic studies

				48.5 (9.8)								
Gholipour et al. (Iran, 2023)	RCT	ESWT + LCI (1 mL triamcinolone acetone + lidocaine)<br>Sham ESWT + LCI (1 mL triamcinolone acetone + lidocaine)	20 (20)<br>20 (20)	45.15 (9.22)<br>44.9 (10.42)	15/5<br>18/2	rESWT	0.03 mJ/mm²	4 Hz	Min. 2600	4/4 weeks	Pret. baseline, month 1, 3, 6 postt.	Pain VAS, GSS
Menekseoglu et al. (Turkey, 2022)	RCT	ESWT + Splinting + Exercise<br>Sham ESWT + Splinting + Exercise	23 (33)<br>22 (33)	43.8 (8.3)<br>46.9 (9.3)	NA<br>NA	rESWT	1.6 Bar	6 Hz	2000	3/3 weeks	Pret. baseline, month 1 postt.	Pain VAS, BCTQ, LANSS, electrodiagnostic studies
Vongvachvasin et al. (Thailand, 2024)	RCT	ESWT + Splinting + Exercise<br>Sham ESWT + Splinting + Exercise	12 (12)<br>12 (12)	60.25 (6.37)<br>58 (10.49)	12/0<br>12/0	fESWT	0.01-0.15 mJ/mm² (depending on pain tolerance)	4-5 Hz	1500	3/3 weeks	Pret. baseline, week 3, 6 postt.	BCTQ (electrodiagnostic studies)
Zong et al. (China, 2023)	Case series	ESWT	16 (27)	54.8 (13.6)	14/2	rESWT	1.5 Bar	10 Hz	1500	5/5 weeks	Pret. baseline, week 1 postt.	Electrodiagnostic studies
Bula-Oyola et al. (Spain, 2017)	SR + MA	ESWT vs. LLLT, US, static and pulsed magnetic fields, PPNL, SWD	1,766 (38 RCTs)	NA	NA	fESWT + rESWT	NA	NA	NA	NA	Multiple and various time points	BCTQ, Pain VAS, hand-grip strength, pinch strength
Huisstede et al. (Netherlands, 2018)	SR	ESWT vs. US, heat wrap therapy, hyperthermia, iontophoresis, phonophoresis, PRF, SWD,	1652 (22 RCTs)	NA	NA	fESWT + rESWT	NA	NA	NA	NA	Multiple and various time points	BCTQ, Pain VAS, hand-grip strength, pinch strength

		TENS, magnets										
Kim et al. (South Korea, 2019)	SR + MA	ESWT vs. Splinting, LCI, Sham ESWT	281 (6 RCTs)	NA	NA	fESWT + rESWT	NA	NA	NA	NA	Multiple and various time points	BCTQ, DASH, R&Ms, electrodiagnostic studies
Li et al. (China, 2020)	SR + MA	ESWT vs. LCI	204 (5 RCTs)	NA	NA	fESWT + rESWT	NA	NA	NA	NA	Multiple and various time points	BCTQ, Pain VAS, electrodiagnostic studies
Xie et al. (China, 2022)	SR + MA	ESWT vs. Splinting, Sham ESWT, PRP, LCI, Cryo-US, nutraceutical supplements	433 (10 RCTs)	NA	NA	fESWT + rESWT	NA	NA	NA	NA	Multiple and various time points	BCTQ, Pain VAS, qDASH, electrodiagnostic studies
Chen et al. (Taiwan, 2022)	SR + MA	ESWT vs. Splinting, nutraceutical supplements	376 (7 RCTs)	NA	NA	fESWT + rESWT	NA	NA	NA	NA	Pret. baseline, week 3-4, 8-10, 12-14 postt.	BCTQ, Pain VAS, electrodiagnostic studies
Zhang et al (China, 2023)	SR + MA	ESWT vs. Sham ESWT, US, Cryo-US, nutraceutical supplements, LCI	857 (19 RCTs)	NA	NA	fESWT + rESWT	NA	NA	NA	NA	Pret. baseline, month 1, 3 6 postt.	BCTQ, Pain VAS, electrodiagnostic studies

RCT = randomized-controlled trial; SR = systematic review; MA = meta-analysis; SD = standard deviation; ESWT = extracorporeal shockwave therapy; fESWT = focused extracorporeal shockwave therapy; rESWT = radial extracorporeal shockwave therapy; LCI = local corticosteroid injection; US = ultrasonography; LLLT = low-level laser therapy; PRP = platelet-rich plasma; ALA = alpha lipoic acid; GLA = gamma linolenic acid; PPNL = polarized polychromatic noncoherent light therapy; transcutaneous Electrical Nerve Stimulation Therapy; SWD = short-wave diathermy therapy; Microwave Diathermy Therapy; PRF = Pulsed Radiofrequency; Pret. baseline = pre-treatment baseline; postt. = post-treatment; VAS = Visual Analog Scale; DASH = Disabilities of the arm, shoulder, and hand score; qDASH = quick DASH; SF-36 = 36-item Short Form Health Survey Questionnaire; R&Ms = Roles and Maudsley score; BCTQ = Boston Carpal Tunnel Questionnaire; ADL = Activities of Daily Activity; GSS = Global Symptoms/System Severity; LANSS = Leeds Assessment of Neuropathic Symptoms and Signs; CSA = nerve cross-sectional area; NA = not available.

Table S8: Carpal tunnel syndrome study characteristics

Authors	Randomization				Intervention								Outcome data					Measurement of outcome						Selection of results				Overall RoB judgment
-	1	2	3	RoB judgment	4	5	6	7	8	9	10	RoB judgment	11	12	13	14	RoB judgment	15	16	17	18	19	RoB judgment	20	21	22	RoB judgment	-
Haghighat et al.	Y	Y	N	Low	N	Y	N	/	/	Y	/	Low	Y	/	/	/	Low	N	N	NI	PY	PN	Moderate	Y	N	N	Low	Moderate
Notarnicola et al. (2022)	Y	Y	N	Low	Y	Y	N	/	/	NI	N	Moderate	NI	N	PY	PN	Moderate	N	N	NI	PY	PN	Moderate	Y	N	N	Low	High
Knobloch et al.	Y	NI	N	Moderate	N	NI	Y	N	/	N	N	Moderate	N	N	PY	PN	Moderate	N	N	Y	PY	PN	Moderate	Y	N	N	Low	High
Saad et al.	Y	NI	N	Moderate	Y	Y	N	/	/	Y	/	Low	Y	/	/	/	Low	N	N	NI	PY	PN	Moderate	Y	N	N	Low	Moderate
Chen et al.	Y	Y	N	Low	N	N	Y	N	/	Y	/	Low	Y	/	/	/	Low	N	N	NI	PY	PN	Moderate	Y	N	N	Low	Moderate
Yildirim et al.	Y	Y	N	Low	Y	Y	Y	N	/	Y	/	Moderate	N	N	N	/	Low	N	N	NI	PY	PN	Moderate	Y	N	N	Low	Moderate
Vahdatpour et al.	Y	NI	N	Moderate	N	Y	Y	N	/	N	N	Moderate	N	N	PY	PN	Moderate	N	N	Y	PY	PN	Moderate	Y	N	N	Low	High
Atthakomol et al.	Y	Y	N	Low	N	Y	Y	N	/	NI	N	Moderate	N	N	N	/	Low	N	N	Y	PY	PN	Moderate	Y	N	N	Low	Moderate
Notarnicola et al. (2015)	Y	NI	N	Moderate	Y	Y	N	/	/	Y	/	Low	Y	/	/	/	Low	N	PY	/	/	/	High	Y	N	N	Low	High
Paoloni et al.	Y	Y	N	Low	Y	Y	N	/	/	Y	/	Low	Y	/	/	/	Low	N	N	N	/	/	Low	Y	N	N	Low	Low
Seok and Kim	Y	Y	N	Low	Y	Y	Y	N	/	N	N	Moderate	N	N	PY	PN	Moderate	N	N	N	/	/	Low	Y	N	N	Low	Moderate
Raissi et al.	Y	NI	N	Moderate	N	Y	N	/	/	Y	/	Low	Y	/	/	/	Low	N	N	N	/	/	Low	Y	N	N	Low	Moderate
Wu et al.	Y	Y	N	Low	N	N	/	/	/	Y	/	Low	Y	/	/	/	Low	N	N	N	/	/	Low	Y	N	N	Low	Low
Ke et al.	Y	Y	N	Low	N	Y	Y	N	/	N	N	Moderate	Y	/	/	/	Low	N	N	NI	PY	PN	Moderate	Y	N	N	Low	Moderate
Xu et al.	Y	Y	N	Low	Y	Y	N	/	/	Y	/	Low	Y	/	/	/	Low	N	N	PN	/	/	Low	Y	N	N	Low	Low
Chang et al.	Y	Y	N	Low	N	N	/	/	/	Y	/	Low	Y	/	/	/	Low	N	N	N	/	/	Low	Y	N	N	Low	Low
Sweilam et al.	Y	NI	N	Moderate	NI	NI	Y	PY	PN	N	N	High	N	N	PY	PN	Moderate	N	N	NI	PY	PN	Moderate	NI	N	N	Moderate	High
Gesslbauer et al.	Y	Y	N	Low	N	Y	Y	PY	Y	N	N	Moderate	N	PY	/	/	Low	N	N	N	/	/	Low	Y	N	N	Low	Moderate
Habibzadeh et al.	Y	Y	N	Low	N	Y	N	/	/	Y	/	Low	Y	/	/	/	Low	N	N	N	/	/	Low	Y	N	N	Low	Low
Durmaz et al.	Y	Y	N	Low	Y	Y	Y	N	/	N	N	Moderate	N	N	PY	PN	Moderate	N	N	Y	PY	PN	Moderate	Y	N	N	Low	High

Razali et al.	Y	Y	N	Low	Y	Y	N	/	/	N	N	Moderate	N	N	PY	PN	Moderate	N	N	NI	PY	PN	Moderate	Y	N	N	Low	High
Saglam et al.	Y	Y	N	Low	Y	Y	N	/	/	N	N	Moderate	Y	/	/	/	Low	N	N	N	/	/	Low	Y	N	N	Low	Moderate
Zhang et al.	Y	Y	N	Low	Y	Y	N	/	/	Y	/	Low	Y	/	/	/	Low	N	N	NI	PY	PN	Moderate	Y	N	N	Low	Moderate
Ghasemi et al.	Y	Y	N	Low	N	Y	N	/	/	Y	/	Low	Y	/	/	/	Low	N	N	N	/	/	Low	Y	N	N	Low	Low
Ulucaköy et al.	Y	Y	N	Low	N	N	/	/	/	N	N	Moderate	N	N	PY	PN	Moderate	N	N	NI	PY	PN	Moderate	Y	N	N	Low	High
Gholipour et al.	Y	Y	N	Low	N	NI	N	/	/	Y	/	Low	Y	/	/	/	Low	N	N	NI	PY	PN	Moderate	Y	N	N	Low	Moderate
Menekseoglu et al.	Y	Y	N	Low	N	N	/	/	/	N	N	Moderate	N	N	PY	PN	Moderate	N	N	NI	PY	PN	Moderate	Y	N	N	Low	High
Vongvachvasin et al.	Y	Y	N	Low	Y	Y	N	/	/	Y	/	Low	Y	/	/	/	Low	N	N	N	/	/	Low	Y	N	N	Low	Low

1 = Was the allocation sequence random?, **2** = Was the allocation sequence concealed until participants were enrolled and assigned to interventions?, **3** = Did baseline differences between intervention groups suggest a problem with the randomization process?, **4** = Were participants aware of their assigned intervention during the trial?, **5** = Were carers and people delivering the interventions aware of participants' assigned intervention during the trial?, **6** = If Y/PY/NI to 4 or 5: Were there deviations from the intended intervention that arose because of the trial context?, **7** = If Y/PY to 6: Were these deviations likely to have affected the outcome?, **8** = If Y/PY/NI to 7: Were these deviations from intended intervention balanced between groups?, **9** = Was an appropriate analysis used to estimate the effect of assignment to intervention?, **10** = If N/PN/NI to 9: Was there potential for a substantial impact (on the result) of the failure to analyze participants in the group to which they were randomized?, **11** = Were data for this outcome available for all, or nearly all, participants randomized?, **12** = If N/PN/NI to 11: Is there evidence that the result was not biased by missing outcome data?, **13** = If N/PN to 12: Could missingness in the outcome depend on its true value?, **14** = If Y/PY/NI to 13: Is it likely that missingness in the outcome depended on its true value?, **15** = Was the method of measuring the outcome inappropriate?, **16** = Could measurement or ascertainment of the outcome have differed between intervention groups?, **17** = If N/PN/NI to 15 and 16: Were outcome assessors aware of the intervention received by study participants?, **18** = If Y/PY/NI to 17: Could assessment of the outcome have been influenced by knowledge of intervention received?, **19** = If Y/PY/NI to 18: Is it likely that assessment of the outcome was influenced by knowledge of intervention received?, **20** = Were the data that produced this result analyzed in accordance with a pre-specified analysis plan that was finalized before unblinded outcome data were available for analysis?, **21** = Is the numerical result being assessed likely to have been selected, on the basis of the results, from multiple eligible outcome measurements (e.g. scales, definitions, time points) within the outcome domain?, **22** = Is the numerical result being assessed likely to have been selected, on the basis of the results, from multiple eligible analyses of the data?, RoB judgment = Risk of Bias judgment (low, moderate (=some concerns) and high), Overall RoB judgement = low (all domains have low RoB), moderate (no high RoB domains, only 2 of 5 domains have moderate RoB) and high (3 of 5 domains have moderate RoB).

Table S9: Cochrane risk-of-bias tool for randomized trials (RoB 2)

Authors	Bias due to confounding				Bias in selection of participants into the study						Bias in classification of intervention		Bias due to deviations from intended interventions		Bias due to missing data		Bias in measurement of the outcome			Bias in selection of the reported result				Overall RoB Judgment
-	1	2	3	Domain RoB	4	5	6	7	8	Domain RoB	9	Domain RoB	10	Domain RoB	11	Domain RoB	12	13	Domain RoB	14	15	16	Domain RoB	-
D'Agostino et al.	Y	N	PY	Low	N	/	/	Y	/	Low	NI	NI	PY	Moderate	N	Low	Y	N	Low	N	NI	N	Moderate	Moderate
Abdulsalam et al.	N	N	PN	Critical	N	/	/	Y	/	Low	NI	NI	NI	NI	N	Low	Y	N	Low	N	NI	N	Moderate	Critical
Taheri et al.	PY	N	Y	Low	N	/	/	Y	/	Low	NI	NI	Y	Low	N	Low	Y	N	Low	N	NI	N	Moderate	Moderate
Vahdatpour et al.	Y	N	PY	Low	N	/	/	Y	/	Low	NI	NI	Y	Low	N	Low	Y	N	Low	N	NI	N	Moderate	Moderate
Malliaro-poulos et al.	Y	N	PY	Low	N	/	/	Y	/	Low	NI	NI	PY	Moderate	N	Low	Y	N	Low	N	NI	N	Moderate	Moderate
Zyluk and Mosiejczuk	PY	N	PN	Moderate	N	/	/	Y	/	Low	NI	NI	NI	NI	N	Low	Y	N	Low	N	NI	N	Moderate	Serious
Dogru et al.	PY	N	PY	Low	N	/	/	Y	/	Low	NI	NI	PY	Moderate	Y	Critical	Y	N	Low	N	NI	N	Moderate	Critical
Zong et al.	N	N	NI	Critical	N	/	/	Y	/	Low	NI	NI	N	Critical	N	Low	Y	N	Low	N	NI	N	Moderate	Critical

1 = Were the measurements of outcomes made at a sufficient pre-intervention time points to permit characterization of pre-intervention trends and patterns?, **2** = Were there extraneous events or changes in context around the time of the intervention that could have influenced the outcome?, **3** = Did the study authors use an appropriate analysis method that accounts for time trends and patterns, and controls for all the important confounding domains?, **4** = Was selection of participants into the study (or into the analysis) based on participant characteristics observed after the start of intervention? If N/PN skip to 7., **5** = If Y/PY to 4: Were the post-intervention variables that influenced selection likely to be associated with intervention?, **6** = If Y/PY to 5: Were the post-intervention variables that influenced selection likely to be influenced by the outcome or a cause of the outcome?, **7** = Do start of follow-up and start of intervention coincide for most participants?, **8** = If Y/PY to 5 and 6, or N/PN to 7: Were adjustment techniques used that are likely to correct for the presence of selection biases?, **9** = Could specification of the distinction between pre-intervention time points and post-intervention time points have been influenced by the outcome data?, **10** = Were the effects of any preparatory (pre-interruption) phases of the intervention appropriately accounted for?, **11** = Were outcome data missing for whole clusters (units of multiple individuals) as well as for individual participants?, **12** = Were methods of outcome assessment comparable before and after the intervention?, **13** = Were there changes in systematic errors in measurement of the outcome coincident with implementation of the intervention?, **14** = Is the reported effect estimate likely to be selected, on the basis of the results, from multiple outcome measurements within the outcome domain?, **15** = Is the reported effect estimate likely to be selected, on the basis of the results, from multiple analyses of the intervention-outcome relationship?, **16** = Is the reported effect estimate likely to be selected, on the basis of the results, from different subgroups?

Table S10: The Risk Of Bias In Non-randomized Studies – of Interventions (ROBINS-I) assessment tool, specifically for interrupted time series and case report

Authors	1	2	3	4	5	6	7	8	9	10	11	12	13	14	15	16	AMSTAR-2 Rating
**Dupuytren’s Disease**																	
Fernando et al. (2024)	Y	Y	N	Y	Y	NA	Y	PY	PY	N	/	/	Y	Y	/	Y	Moderate quality
**Carpal Tunnel Syndrome**																	
Bula-Oyola et al. (2017)	Y	Y	N	PY	Y	Y	N	N	Y	N	Y	N	N	Y	N	Y	Critically low quality
Kim et al. (2019)	Y	N	Y	Y	Y	Y	Y	Y	Y	Y	Y	N	N	Y	N	Y	Critically low quality
Li et al. (2020)	Y	Y	Y	Y	Y	Y	N	Y	Y	Y	Y	Y	N	Y	Y	Y	Low quality
Xie et al. (2022)	Y	Y	Y	PY	Y	Y	N	Y	Y	N	Y	N	N	Y	N	Y	Critically low quality
Chen et al. (2022)	Y	N	Y	Y	Y	Y	Y	Y	Y	Y	Y	Y	Y	Y	N	N	Critically low quality
Zhang et al (2023)	Y	N	Y	Y	Y	Y	PY	Y	Y	Y	Y	Y	Y	Y	N	Y	Critically low quality

Table S11: The AMSTAR-2 rating systems
green = yes, orange = partially yes, red = no, grey = not available.
AMSTAR-2 is a tool to assess systematic reviews. It uses 16 items to evaluate a systematic review:
“1. Did the research questions and inclusion criteria for the review include the components of PICO?
2. Did the report of the review contain an explicit statement that the review methods were established prior to the conduct of the review and did the report justify any significant deviations from the protocol?
3. Did the review authors explain their selection of the study designs for inclusion in the review?
4. Did the review authors use a comprehensive literature search strategy?
5. Did the review authors perform study selection in duplicate?
6. Did the review authors perform data extraction in duplicate?
7. Did the review authors provide a list of excluded studies and justify the exclusions?
8. Did the review authors describe the included studies in adequate detail?
9. Did the review authors use a satisfactory technique for assessing the risk of bias (RoB) in individual studies that were included in the review?
10. Did the review authors report on the sources of funding for the studies included in the review?
11. If meta-analysis was performed, did the review authors use appropriate methods for statistical combination of results?
12. If meta-analysis was performed, did the review authors assess the potential impact of RoB in individual studies on the results of the meta-analysis or other evidence synthesis?
13. Did the review authors account for RoB in primary studies when interpreting/discussing the results of the review?
14. Did the review authors provide a satisfactory explanation for, and discussion of, any heterogeneity observed in the results of the review?
15. If they performed quantitative synthesis did the review authors carry out an adequate investigation of publication bias (small study bias) and discuss its likely impact on the results of the review?
16. Did the review authors disclose any potential sources of conflict of interest, including any funding they received for conducting the review?”
Seven of those items (items 2, 4, 7, 9, 11, 13, 15) are considered critically and the other 9 items (items 1, 3, 5, 6, 8, 10, 12, 14, 16) are non-critical. A SR is considered high or moderate quality if only one or more non-critical items are not fulfilled. Low and critically low quality is established if one or more critical items with or without non-critical weaknesses are not fulfilled.

Authors (Country, Year)	Level of Evidence	Postt. follow-up	Significant changes		No significant changes		Adverse effects	Notes
Knobloch et al. (Germany, 2022)	II	-	**ESWT (A)**	**Sham ESWT (B)**	**ESWT group**	**Sham ESWT**	No	/
		**Month 3**	Pain VAS ↓ (cb, ccB)	Pain VAS ↑ (cb)	Hand-grip strength, URAM, DASH, MHQ	Hand-grip strength, URAM, DASH, MHQ		
		**Month 6**	Pain VAS ↓ (cb, ccB)	Pain VAS ↑ (cb)				
		**Month 12**	Pain VAS ↓ (cb, ccB)	Pain VAS ↑ (cb)				
		**Month 18**	Pain VAS ↓ (cb, ccB)	Pain VAS ↑ (cb)				
Saad et al. (Egypt, 2021)	II	-	**ESWT + Splinting + Stretching (A)**	**LIPUS + Splinting + Physiotherapy (B)**	**ESWT + Splinting + Stretching**	**LIPUS + Splinting + Physiotherapy**	No	/
		**Week 6**	qDASH ↓ (cb, ccB), hand-grip strength ↑ (cb, ccB)	qDASH ↓ (cb), hand-grip strength ↑ (cb)	/	/		
Abdulsalam et al. (Kuweit, 2019)	IV	**Week 8**	MHQ for tenderness ↓ (cb), MHQ for pain ↓ (cb)		Hand-grip strength		No	/
Taheri et al. (Iran, 2022)	II	**Week 6**	Pain VAS ↓ (cb), DASH ↓ (cb), MCP contraction angle ↓ (cb)		/		Slight pain 1-2 days after the session	/
		**Week 14**	Pain VAS ↓ (cb, cp), DASH ↓ (cb, cp), MCP contraction angle ↓ (cb, cp)					
Fernando et al. (UK, 2024)	III	**Up to week 14**	*Included 8 studies evaluating ESWT<br>Pain VAS ↓ (4 of 8 studies), ROM ↑ (4 of 4 studies), Hand-grip strength ↑ (1 of 4 studies), R&Ms and MHQ ↑ (one study each)		/		No	SR included all the other 4 studies.

ESWT = Extracorporeal Shockwave Therapy; LIPUS = Low-intensity pulsed ultrasound; postt. = post-treatment; VAS = Visual Analog Scale; DASH = Disabilities of the arm, shoulder, and hand score; MHQ = Michigan Health Questionnaire; URAM = Unité Rhumatologique des Affections de la Main; MCP contraction angle = metacarpophalangeal contraction angle; NA = Not Available.

There were no significant differences at baseline between the groups. The following abbreviations and symbols specify in which relation the specific outcome measure is significant: cb = compared to baseline significantly different; cp = compared to previous value significantly different; ccX = compared to group X (if existent) at the same follow-up; ↑ = significant increase; ↓ = significant decrease. *Example: VAS ↓ (cp, cc) = The VAS score is significantly lower than the previously recorded VAS score of the same group and is significantly lower than the VAS score of the other group at the same follow-up.* In the column of "No significant changes" are only outcome measures that yielded no significant changes at all in respective group.

Table S12: Dupuytren's disease study results

Authors (Country, Year)	Level of Evidence	Postt. follow-up	Significant changes			No significant changes			Adverse effects	Notes
Vahdatpour et al. (Iran, 2020)	II	**Week 0**	Pain VAS ↓ (cb), Function VAS ↓ (cb)			/			No	Authors did not report the result of the Quinnell Trigger finger and DASH score.
		**Week 6**	Pain VAS ↓ (cb, cp), Triggering VAS ↓ (cb, cp), Function VAS ↓ (cb, cp)							
		**Week 18**	Pain VAS ↓ (cb), Triggering VAS ↓ (cb), Function VAS ↓ (cb)							
Malliaropoulos et al. (Greece, 2016)	II	**Month 1**	Pain VAS ↓ (cb)			/			/	Strong, positive, significant correlation between pre-ESWT treatment symptoms duration and no. of sessions required. No intra-group comparison between the post-treatment follow-up values.
		**Month 3**	Pain VAS ↓ (cb)							
		**Month 12**	Pain VAS ↓ (cb),<br>R&Ms = 73.5% "excellent", 20.4% "good", 6.1% "fair"							
Chen et al. (Taiwan, 2021)	II	**-**	**High-energy ESWT (A)**	**Low-energy ESWT (B)**	**Sham ESWT (C)**	**High-energy ESWT**	**Low-energy ESWT**	**Sham ESWT**	No	/
		**Month 1**	/	/	/	TFA	Pain VAS, qDASH, TFA	Pain VAS, qDASH, TFA		
		**Month 3**	/	/	/					
		**Month 6**	Pain VAS ↓ (cb, ccB, ccC), qDASH ↓ (cb, ccB, ccC)	/	/					
Yildirim et al. (Turkey, 2016)	II	**-**	**ESWT (A)**		**LCI (B)**	**ESWT**		**LCI**	No	/
		**Month 1**	Pain VAS ↓ (cb), TFAfq ↓ (cb), TFAs ↓ (cb), TFAfi ↓ (cb), qDASH ↓ (cb)		Pain VAS ↓ (cb), TFAfq ↓ (cb), TFAs ↓ (cb), TFAfi ↓ (cb), qDASH ↓ (cb)	/		/		
		**Month 3**	Pain VAS ↓ (cb), TFAfq ↓ (cb), TFAs ↓ (cb), TFAfi ↓ (cb), qDASH ↓ (cb)		Pain VAS ↓ (cb), TFAfq ↓ (cb), TFAs ↓ (cb), TFAfi ↓ (cb), qDASH ↓ (cb)					
		**Month 6**	Pain VAS ↓ (cb), TFAfq ↓ (cb), TFAs ↓ (cb), TFAfi ↓ (cb), qDASH ↓ (cb)		Pain VAS ↓ (cb), TFAfq ↓ (cb), TFAs ↓ (cb), TFAfi ↓ (cb), qDASH ↓ (cb)					

Zyluk and Mosiejczuk (Poland, 2020)	II	**Week 1**	Pain NRS ↓ (cb), Froimson Triggering = 38% (19 fingers) grade I, 58% (29 fingers) grade II, 4% (2 fingers) grade III (unchanged)	/	No	Recovered = no tenderness
		**Month 3**	Pain NRS ↓ (cb), Froimson Triggering = 92% (46 fingers) recovered			
Dogru et al. (Turkey, 2020)	II	**Month 0**	/	/	/	/
		**Month 3**	Pain NRS ↓ (cb), qDASH ↓ (cb), hand-grip strength ↑ (cb), pinch strength ↑ (cb), ROM ↑ (cb)			

ESWT = Extracorporeal Shockwave Therapy; LCI = Local Corticosteroid Injection; postt. = post-treatment; VAS = Visual Analog Scale; NRS = Numeric Rating Scale; DASH = Disabilities of the arm, shoulder, and hand score; qDASH = quick DASH; ROM = Range of Motion; R&Ms = Roles and Maudsley score; TFAf = Trigger Finger Assessment of frequency; TFAi = Trigger Finger Assessment of functional impact; TFAs = Trigger Finger Assessment of severity of triggering; NA = Not Available.
There were no significant differences at baseline between the groups. The following abbreviations and symbols specify in which relation the specific outcome measure is significant:
cb = compared to baseline significantly different; cp = compared to previous value significantly different; ccX = compared to group X (if existent) at the same follow-up; ↑ = significant increase; ↓ = significant decrease.
Example: VAS ↓ (cp, cc) = The VAS score is significantly lower than the previously recorded VAS score of the same group and is significantly lower than the VAS score of the other group at the same follow-up. In the column of “No significant changes” are only outcome measures that yielded no significant changes at all in respective group.

Table S13: Trigger finger study results

Authors (Country, Year)	Level of Evidence	Postt. follow-up	Significant changes		No significant changes		Adverse effects	Notes
Haghighat et al. (Iran, 2020)	II	-	**ESWT (A)**	**Sham ESWT (B)**	**ESWT**	**Sham ESWT**	/	/
		**Week 0**	Pain VAS ↓ (cb, ccB), DASH ↓ (cb, ccB)	DASH ↓ (cb)	Hand-grip strength	Pain VAS, hand-grip strength		
		**Week 3**	Pain VAS ↓ (cp, ccB), DASH ↓ (cb, cp, ccB)	DASH ↓ (cb, cp)				
		**Week 6**	Pain VAS ↓ (cp, ccB), DASH ↓ (cb, cp, ccB)	DASH ↓ (cb, cp)				
Notarnicola et al. (Italy, 2022)	II	-	**ESWT (A)**	**LCI (B)**	**ESWT**	**LCI**	No	/
		**Month 3**	Pain VAS ↓ (cb), DASH ↓ (cb), SF-36 ↑ (cb), hand-grip strength ↑ (cb), Ritchie's tenderness ↓ (cb)	Pain VAS ↓ (cb), DASH ↓ (cb), SF-36 ↑ (cb), hand-grip strength ↑ (cb), Ritchie's tenderness ↓ (cb)	/	/		
		**Month 6**	Pain VAS ↓ (cb, cp), DASH ↓ (cb, cp), SF-36 ↑ (cb, cp), hand-grip strength ↑ (cb, cp), Ritchie's tenderness ↓ (cb, cp)	Pain VAS ↓ (cb, cp), DASH ↓ (cb, cp), SF-36 ↑ (cb, cp), hand-grip strength ↑ (cb, cp), Ritchie's tenderness ↓ (cb, cp)				

ESWT = Extracorporeal Shockwave Therapy; LCI = Local Corticosteroid Injection; postt. = post-treatment; VAS = Visual Analog Scale; DASH = Disabilities of the arm, shoulder, and hand score; SF-36 = 36-item Short Form Health Survey Questionnaire.
There were no significant differences at baseline between the groups. The following abbreviations and symbols specify in which relation the specific outcome measure is significant:
cb = compared to baseline significantly different; cp = compared to previous value significantly different; ccX = compared to X group (if existent) at the same follow-up; ↑ = significant increase; ↓ = significant decrease.
Example: VAS ↓ (cp, cc) = The VAS score is significantly lower than the previously recorded VAS score of the same group and is significantly lower than the VAS score of the other group at the same follow-up. In the column of "No significant changes" are only outcome measures that yielded no significant changes at all in respective group.

Table S14: DeQuervain's tenosynovitis study results

Authors (Country, Year)	Level of Evidence	Postt. follow-up	Significant changes	No significant changes	Adverse effects	Notes
D'Agostino et al. (Italy, 2011)	II	**Month 0**	/	/	No	MRI scans showed a reduction of the bone marrow edema postt. but was not quantified.
		**Month 2**	Pain VAS ↓ (cb), ROM ↑ (cb)			
		**Month 6**	Pain VAS ↓ (cb), ROM ↑ (cb)			
		**Year 1**	Pain VAS ↓ (cb), ROM ↑ (cb)			

Postt. = post-treatment; VAS = Visual Analog Scale; ROM = Range of Motion.
There were no significant differences at baseline between the groups. The following abbreviations and symbols specify in which relation the specific outcome measure is significant:
cb = compared to baseline significantly different; cp = compared to previous value significantly different; ccX = compared to group X (if existent) at the same follow-up; ↑ = significant increase; ↓ = significant decrease.
Example: VAS ↓ (cp, cc) = The VAS score is significantly lower than the previously recorded VAS score of the same group and is significantly lower than the VAS score of the other group at the same follow-up. In the column of "No significant changes" are only outcome measures that yielded no significant changes at all in respective group.

Table S15: Osteonecrosis of the lunate study results

Authors (Country, Year)	Level of Evidence	Postt. follow-up	Significant changes			No significant changes			Adverse effects	Notes
Vahdatpour et al. (Iran, 2016)	II	-	**ESWT + Splinting (A)**	**Sham ESWT + Splinting (B)**		**ESWT**	**Sham ESWT**		/	/
		**Month 3**	Pain VAS ↓ (cb, ccB), BCTQs ↓ (cb, ccB), BCTQf ↓ (cb, ccB) CMAPL ↓ (ccB), SNAPL ↓ (ccB)	Pain VAS ↓ (cb), BCTQf ↓ (cb) CMAPL ↓ (cb), SNAPL ↓ (cb)		/	BCTQs			
		**Month 6**	Pain VAS ↓ (cb, ccB), BCTQs ↓ (cb, ccB), BCTQf ↓ (cb, ccB) CMAPL ↓ (cb, ccB), SNAPL ↓ (cb, ccB)	Pain VAS ↓ (cb) & VAS ↑ (cp), BCTQf ↓ (cb)		/	BCTQs CMAPL, SNAPL			
Atthakomol et al. (Thailand, 2018)	II	-	**ESWT (A)**	**LCI (B)**		**ESWT**	**LCI**		Slight pain only during the treatment session.	Electrodiagnostic studies were conducted only at pre-treatment baseline and week 12 post-treatment. DSL at baseline differed with only a p-value of 0.057.
		**Week 1**	/	BCTQs ↓ (cb)		Pain VAS, BCTQs, BCTQf	Pain VAS, BCTQf			
		**Week 4**	BCTQs ↓ (cb)	BCTQs ↓ (cb)		Pain VAS, BCTQf	Pain VAS, BCTQf			
		**Week 12**	Pain VAS ↓ (cb), BCTQs ↓ (cb), BCTQf ↓ (cb) DSL ↓ (cb)	DSL ↓ (cb, ccA)		SNAPA, DML, CMAPA	Pain VAS, BCTQs, BCTQf SNAPA, DML, CMAPA			
		**Week 24**	Pain VAS ↓ (cb), BCTQs ↓ (cb, ccB), BCTQf ↓ (cb, ccB)	/		/	Pain VAS, BCTQs, BCTQf			
Notarnicola et al. (Italy, 2015)	**II**	-	**ESWT (A)**	**Nutraceutical group (B)**		**ESWT**	**Nutraceutical group**		/	R&Ms and electrodiagnostic studies were conducted only at pre-treatment baseline and month 6 post-treatment.
		**Month 1**	Pain VAS ↓ (cb), BCTQs ↓ (cb), BCTQf ↓ (cb)	Pain VAS ↓ (cb), BCTQs ↓ (cb), BCTQf ↓ (cb)		/	/			
		**Month 2**	Pain VAS ↓ (cb), BCTQs ↓ (cb), BCTQf ↓ (cb)	Pain VAS ↓ (cb), BCTQs ↓ (cb), BCTQf ↓ (cb)		/	/			
		**Month 4**	Pain VAS ↓ (cb), BCTQs ↓ (cb), BCTQf ↓ (cb)	Pain VAS ↓ (cb), BCTQs ↓ (cb), BCTQf ↓ (cb)		/	/			
		**Month 6**	Pain VAS ↓ (cb), BCTQs ↓ (cb), BCTQf ↓ (cb), R&Ms = 88.2% "excellent" or "good" DML ↓ (cb), SNCV ↑ (cb)	Pain VAS ↓ (cb), BCTQs ↓ (cb), BCTQf ↓ (cb), R&Ms = 100% "excellent" or "good" DML ↓ (cb), SNCV ↑ (cb)		/	/			
Paoloni et al. (Italy, 2016)	I	-	**ESWT (A)**	**US (B)**	**Cryo-US (C)**	**ESWT**	**US**	**Cryo-US**	No	/
		**Week 0**	Pain VAS ↓ (cb), Paresthesia VAS ↓ (cb), BCTQs ↓ (cb), BCTQf ↓ (cb)	Pain VAS ↓ (cb), Paresthesia VAS ↓ (cb), BCTQs ↓ (cb), BCTQf ↓ (cb)	Pain VAS ↓ (cb), Paresthesia VAS ↓ (cb), BCTQs ↓ (cb, ccA, ccB), BCTQf ↓ (cb)	/	/	/		

Attachment 2 to: Al-Mousllie I, Vogt PM, Jokuszies A. State of shock – a systematic review of extracorporeal shockwave therapy in hand surgery. GMS Interdiscip Plast Reconstr Surg DGPW. 2025;14:Doc03. DOI: 10.3205/iprs000192

		**Week 4**	Pain VAS ↓ (cb), Paresthesia VAS ↓ (cb), BCTQs ↓ (cb), BCTQf ↓ (cb)	Paresthesia VAS ↓ (cb), BCTQs ↓ (cb), BCTQf ↓ (cb)	Pain VAS ↓ (cb), Paresthesia VAS ↓ (cb), BCTQs ↓ (cb, ccA, ccB), BCTQf ↓ (cb)	/	Pain VAS	/		
		**Week 12**	Pain VAS ↓ (cb), BCTQs ↓ (cb), BCTQf ↓ (cb)	BCTQs ↓ (cb), BCTQf ↓ (cb)	Pain VAS ↓ (cb), BCTQs ↓ (cb, ccA, ccB), BCTQf ↓ (cb)	Paresthesia VAS	Pain VAS, Paresthesia VAS	Paresthesia VAS		

Seok and Kim (Italy, 2013)	II	-	**ESWT (A)**	**LCI (B)**	**ESWT**	**LCI**	No	/
		**Month 1**	Pain VAS ↓ (cb), BCTQs ↓ (cb)	Pain VAS ↓ (cb)<br>SNAPA ↑ (cb), DSL ↓ (cb), DML ↓ (cb)	BCTQf<br>NCV, SNAPA, CMAPA, DSL, DML	BCTQs, BCTQf<br>CMAPA		
		**Month 3**	Pain VAS ↓ (cb), BCTQs ↓ (cb)	Pain VAS ↓ (cb), BCTQs ↓ (cb)<br>DSL ↓ (cb)	BCTQf<br>NCV, SNAPA, CMAPA, DSL, DML	BCTQf<br>SNAPA, CMAPA, DML		
Raissi et al. (Iran, 2017)	II	-	**ESWT + Splinting (A)**	**Splinting (B)**	**ESWT**	**Splinting**	Slight transient pain	/
		**Week 3**	Pain VAS ↓ (cb), qDASH ↓ (cb)<br>SNAPL↓ (cb)	Pain VAS ↓ (cb)<br>SNAPL↓ (cb)	SNAPA, CMAPL, CMAPA	qDASH<br>SNAPA, CMAPL, CMAPA		
		**Week 8**	Pain VAS ↓ (cb), qDASH ↓ (cb)<br>SNAPL↓ (cb), CMAPL ↓ (cb)	Pain VAS ↓ (cb), qDASH ↓ (cb)<br>SNAPL↓ (cb), CMAPL ↓ (cb)	SNAPA, CMAPA	SNAPA, CMAPA		
		**Week 12**	Pain VAS ↓ (cb), qDASH ↓ (cb)<br>SNAPL↓ (cb)	Pain VAS ↓ (cb), qDASH ↓ (cb)<br>SNAPL↓ (cb), CMAPL ↓ (cb)	SNAPA, CMAPL, CMAPA	SNAPA, CMAPA		
Wu et al. (China, 2016)	I	-	**ESWT + Splinting (A)**	**Sham ESWT + Splinting (B)**	**ESWT**	**Sham ESWT**	No	/
		**Week 1**	Pain VAS ↓ (cb, ccB), BCTQs ↓ (cb, ccB), BCTQf ↓ (cb, ccB), finger pinch ↑ (cb)<br>CSA ↓ (cb)	Pain VAS ↓ (cb), BCTQs ↓ (cb), BCTQf ↓ (cb), finger pinch ↑ (cb)<br>CSA ↓ (cb), SNCV ↑ (cb)	SNCV	/		
		**Week 4**	Pain VAS ↓ (cb, ccB), BCTQs ↓ (cb, ccB), BCTQf ↓ (cb, ccB), finger pinch ↑ (cb)<br>CSA ↓ (cb), SNCV ↑ (cb)	Pain VAS ↓ (cb), BCTQs ↓ (cb), BCTQf ↓ (cb), finger pinch ↑ (cb)<br>CSA ↓ (cb), SNCV ↑ (cb)	/	/		
		**Week 8**	Pain VAS ↓ (cb, ccB), BCTQs ↓ (cb, ccB), BCTQf ↓ (cb, ccB), finger pinch ↑ (cb)<br>CSA ↓ (cb, ccB), SNCV ↑ (cb)	Pain VAS ↓ (cb), BCTQs ↓ (cb), finger pinch ↑ (cb)<br>CSA ↓ (cb), SNCV ↑ (cb)	/	BCTQf		
		**Week 12**	Pain VAS ↓ (cb, ccB), BCTQs ↓ (cb), BCTQf ↓ (cb, ccB), finger pinch ↑ (cb)<br>CSA ↓ (cb, ccB), SNCV ↑ (cb)	Pain VAS ↓ (cb), BCTQs ↓ (cb), finger pinch ↑ (cb)<br>CSA ↓ (cb), SNCV ↑ (cb)	/	BCTQf		

Ke et al.<br>(China, 2016)	II	-	**3x Sessions ESWT (A)**	**1x Sessions ESWT (B)**	**Sham ESWT (C)**	**3x Sessions ESWT**	**1x Sessions ESWT**	**Sham ESWT**	No	The significance levels of the post-treatment values to baseline were not reported.
		**Week 2**	BCTQs ↓ (ccB, ccC), BCTQf ↓ (ccB, ccC)	/	/	SNCV, CSA	BCTQs, BCTQf SNCV, CSA	BCTQs, BCTQf SNCV, CSA		
		**Week 8**	BCTQs ↓ (ccB, ccC), BCTQf ↓ (ccB, ccC)	/	/	SNCV, CSA	BCTQs, BCTQf SNCV, CSA	BCTQs, BCTQf SNCV, CSA		
		**Week 12**	BCTQs ↓ (ccB, ccC), BCTQf ↓ (ccB, ccC)<br>CSA ↓ (ccC)	/	/	CSA	BCTQs, BCTQf SNCV, CSA	BCTQs, BCTQf SNCV, CSA		
Xu et al.<br>(China, 2020)	I	-	**ESWT (A)**	**LCI (B)**		**ESWT**	**LCI**		Slight transient pain	/
		**Week 3**	Pain VAS ↓ (cb), BCTQ ↓ (cb)	Pain VAS ↓ (cb), BCTQ ↓ (cb)		SNAPA, CMAPA, SNAPL, CMAPL	SNAPA, CMAPA, SNAPL, CMAPL			
		**Week 9**	Pain VAS ↓ (cb, ccB), BCTQ ↓ (cb, ccB)<br>SNAPL ↓ (cb)	BCTQ ↓ (cb)		SNAPA, CMAPA, CMAPL	Pain VAS<br>SNAPA, CMAPA, SNAPL, CMAPL			
		**Week 12**	Pain VAS ↓ (cb, ccB), BCTQ ↓ (cb, ccB)<br>SNAPL ↓ (cb, ccB), CMAPL ↓ (cb)	/		SNAPA, CMAPA	Pain VAS, BCTQ<br>SNAPA, CMAPA			
Chang et al.<br>(China, 2020)	I	-	**ESWT + PRP (A)**	**Sham ESWT + PRP (B)**		**ESWT**	**Sham ESWT**		No	/
		**Month 1**	BCTQs ↓ (cb, ccB), BCTQf ↓ (cb)<br>SNCV ↑ (cb), DML ↓ (cb), CSA ↓ (cb)	BCTQs (cb), BCTQf (cb)<br>SNCV ↑ (cb, ccA), DML ↓ (cb), CSA ↓ (cb)		/	/			
		**Month 3**	BCTQs ↓ (cb), BCTQf ↓ (cb)<br>SNCV ↑ (cb), DML (cb, ccB), CSA ↓ (cb)	BCTQs ↓ (cb), BCTQf ↓ (cb)<br>SNCV ↑ (cb), DML ↓ (cb), CSA ↓ (cb)		/	/			
		**Month 6**	BCTQs ↓ (cb), BCTQf ↓ (cb)<br>SNCV ↑ (cb), DML ↓ (cb), CSA ↓ (cb)	BCTQs ↓ (cb), BCTQf ↓ (cb)<br>SNCV ↑ (cb), DML ↓ (cb), CSA ↓ (cb)		/	/			
Sweilam et al.<br>(Egypt, 2019)	**II**	-	**ESWT (A)**	**LCI (B)**		**ESWT**	**LCI**		/	/
		**Week 2**	Pain VAS ↓ (cb), BCTQ ↓ (cb)<br>DML ↓ (cb), CMAPA ↑ (cb)	Pain VAS ↓ (cb), BCTQ ↓ (cb)<br>DML ↓ (cb)		/	CMAPA			
		**Week 4**	Pain VAS ↓ (cb), BCTQ ↓ (cb, cp)<br>DML ↓ (cb), CMAPA ↑ (cb)	Pain VAS (cb), BCTQ (cb)<br>DML ↓ (cb), CMAPA ↑ (cb)		/<br>NCV	/<br>NCV			
	II	-	**ESWT (A)**	**Sham ESWT (B)**		**ESWT**	**Sham ESWT**		No	

Gesslbauer et al. (Austria, 2021)		**Week 3**	Pain VAS ↓ (cb), SF-36 ↑ (cb)	/		Hand-grip strength, BCTQs, BCTQf DML, SNCV	Pain VAS, SF-36, hand-grip strength, BCTQs, BCTQf DML, SNCV			Electrodiagnostic studies were only conducted at pre-treatment baseline and at week 12 post-treatment.
		**Week 12**	Pain VAS ↓ (cb), hand-grip strength ↑ (cb), BCTQs ↓ (cb, cp) DML ↓ (cb), SNCV ↑ (ccB)	/		SF-36, BCTQf	Pain VAS, SF-36, hand-grip strength, BCTQs, BCTQf DML, SNCV			
Habibzadeh et al. (Iran, 2022)	I	**-**	**Point ESWT (A)**	**Sweep ESWT (B)**	**Physiotherapy (C)**	**Point ESWT**	**Sweep ESWT**	**Physio-therapy**	Slight transient pain and redness after treatment.	Electrodiagnostic studies were only conducted at pre-treatment baseline and at week 1 post-treatment.
		**Week 1**	Pain VAS ↓ (cb, ccC), Paresthesia VAS ↓ (cb, ccC), BCTQs ↓ (cb, ccC), BCTQf ↓ (cb) DSL↓ (cb, ccC), DML↓ (cb)	Pain VAS ↓ (cb, ccC), Paresthesia VAS ↓ (cb, ccC), BCTQs ↓ (cb, ccC), BCTQf ↓ (cb) DSL↓ (cb, ccC), DML↓ (cb, ccC)	Pain VAS ↓ (cb), Paresthesia VAS ↓ (cb), BCTQs ↓ (cb), BCTQf ↓ (cb)	/	/	DSL, DML		
		**Week 4**	Pain VAS ↓ (cb, cp, ccC), Paresthesia VAS ↓ (cb, cp, ccC), BCTQs ↓ (cb, cp, ccC), BCTQf ↓ (cb)	Pain VAS ↓ (cb, cp, ccC), Paresthesia VAS ↓ (cb, cp, ccC), BCTQs ↓ (cb, cp, ccC), BCTQf ↓ (cb, ccC)	Pain VAS ↓ (cb), Paresthesia VAS ↓ (cb), BCTQs ↓ (cb)	/	/	BCTQf		
Durmaz et al. (Turkey, 2022)	**II**	**-**	**ESWT (A)**	**LCI (B)**	**Splinting (C)**	**ESWT**	**LCI**	**Splinting**	No	Electrodiagnostic studies were only conducted at pre-treatment baseline and at week 12 post-treatment. SNAP values differed at pre-treatment baseline already and therefore SNAP values was excluded.
		**Week 1**	Pain VAS ↓ (cb), Numbness VAS ↓ (cb), BCTQs ↓ (cb)	Pain VAS ↓ (cb, ccA, ccC), Numbness VAS ↓ (cb, ccA, ccC), BCTQs ↓ (cb), BCTQf ↓ (cb, ccA), hand-grip strength ↑ (cb)	Pain VAS ↓ (cb), Numbness VAS ↓ (cb), BCTQs ↓ (cb)	hand-grip strength, BCTQf	/	hand-grip strength, BCTQf		
		**Week 12**	Pain VAS ↓ (cb), Numbness VAS ↓ (cb), BCTQs ↓ (cb), BCTQf ↓ (cb) CMAPA ↑ (cb), SNCV ↑ (cb)	Pain VAS ↓ (cb, ccA), Numbness VAS ↓ (cb), BCTQs ↓ (cb, ccA, ccC), BCTQf ↓ (cb, ccA), hand-grip strength ↑ (cb) DML ↓ (cb, ccC)	Pain VAS ↓ (cb), Numbness VAS ↓ (cb), BCTQs ↓ (cb, ccA), BCTQf ↓ (cb) DML ↓ (cb, ccA), SNCV ↑ (cb)	Hand-grip strength, SNAPA, DML, MNCV	SNAP, CMAPA, SNCV, MNCV	Hand-grip strength, SNAPA, CMAPA, MNCV		
Razali et al. (Malaysia, 2022)	II	**-**	**US + physiotherapy (A)**	**ESWT + physiotherapy (B)**	**Physiotherapy (C)**	**US + physio-therapy**	**ESWT + physio-therapy**	**Physio-therapy**	No	/
		**Week 2 (of treatment)**	/	BCTQs ↓ (cb, ccA, ccC), BCTQf ↓ (cb, ccA, ccC)	/	BCTQs, BCTQf	/	BCTQs, BCTQf		
		**Week 0**	BCTQs ↓ (cb, ccC), BCTQf ↓ (cb, ccC)	BCTQs ↓ (cb, cp, ccA, ccC), BCTQf ↓ (cb, cp, ccA, ccC)	/	/	/	BCTQs, BCTQf		
		**Week 4**	BCTQs ↓ (cb, ccC), BCTQf ↓ (cb, ccC)	BCTQs ↓ (cb, ccA, ccC), BCTQf ↓ (cb, ccA, ccC)	BCTQf ↓ (cb)	/	/	BCTQs		

Saglam et al. (Turkey, 2022)	II	-	**Splinting + Exercise (A)**	**ESWT + Exercise (B)**	**Physiotherapy (C)**		**Splinting + Exercise**	**ESWT + Exercise**	**Physical therapy**		No	/
		**Week 3**	Pain VAS ↓ (cb), BCTQs ↓ (cb), BCTQf ↓ (cb), LANSS ↓ (cb)<br>SNCV ↑ (cb)	Pain VAS ↓ (cb, ccA, ccC), BCTQs ↓ (cb, ccA, ccC), BCTQf ↓ (cb, ccA, ccC), LANSS ↓ (cb, ccA, ccC)<br>SNCV ↑ (cb, ccA, ccC)	Pain VAS ↓ (cb, ccA), BCTQs ↓ (cb, ccA), BCTQf ↓ (cb, ccA), LANSS ↓ (cb, ccA)<br>SNCV ↑ (cb, ccA)		/	/	/			
		**Week 12**	Pain VAS ↓ (cb), BCTQs ↓ (cb), BCTQf ↓ (cb), LANSS ↓ (cb)<br>SNCV ↑ (cb)	Pain VAS ↓ (cb, ccA, ccC), BCTQs ↓ (cb, ccA, ccC), BCTQf ↓ (cb, ccA, ccC), LANSS ↓ (cb, ccA, ccC)<br>SNCV ↑ (cb, ccA, ccC)	Pain VAS ↓ (cb, ccA), BCTQs ↓ (cb, ccA), BCTQf ↓ (cb, ccA), LANSS ↓ (cb, ccA)<br>SNCV ↑ (cb, ccA)		/	/	/			
Zhang et al. (China, 2023)	II	-	**ESWT (A)**		**Physiotherapy + Splinting (B)**		**ESWT**		**Physiotherapy + Splinting**		Swelling and numbness (but significantly less frequent in in group A than in group B).	Authors did not clarify at which time point exactly the measurement of BCTQ and the electrodiagnostic studies were conducted. Therefore, the results reported under “After treatment”. The results for the measured ADL score were not reported properly.
		**Week 1**	Pain VAS ↓ (cb, ccB), GSS ↓ (cb, ccB)		/		/		Pain VAS, GSS			
		**Week 2**	Pain VAS ↓ (cb, ccB), GSS ↓ (cb, ccB)		Pain VAS ↓ (cb), GSS ↓ (cb)		/		/			
		**After treatment**	BCTQs ↓ (cb, ccB), BCTQf ↓ (cb, ccB)<br>DML ↓ (cb, ccB), CMAPA ↑ (cb, ccB), SNCV ↑ (cb, ccB), SNAPA ↑ (cb, ccB)		BCTQs ↓ (cb), BCTQf ↓ (cb)		/		DML, CMAPA, SNCV, SNAPA			
Ghasemi et al. (Iran, 2024)	I	-	**ESWT (A)**		**LLLT (B)**		**ESWT**		**LLLT**		/	/
		**Week 2**	Pain VAS ↓ (cb), CSA ↓ (cb)		Pain VAS ↓ (cb), CSA ↓ (cb)		/		/			
Ulucaköy et al. (Turkey, 2020)	II	-	**Splinting (A)**	**Splinting + ESWT (B)**	**ESWT (C)**	**Splinting + Sham ESWT (D)**	**Splinting**	**Splinting + ESWT**	**ESWT**	**Splinting + ESWT**	No	Electrodiagnostic studies were conducted to establish a baseline and at month 3 post-treatment.
		**Month 1**	Pain VAS ↓ (cb), BCTQs ↓ (cb), BCTQf ↓ (cb), LANSS ↓ (cb)	Pain VAS ↓ (cb), BCTQs ↓ (cb), BCTQf ↓ (cb), finger pinch ↑ (cb, ccA, ccD), LANSS ↓ (cb)	Pain VAS ↓ (cb), BCTQs ↓ (cb), BCTQf ↓ (cb), finger pinch ↑ (cb), LANSS ↓ (cb)	Pain VAS ↓ (cb), BCTQs ↓ (cb), BCTQf ↓ (cb), finger pinch ↑ (cb), LANSS ↓ (cb)	finger pinch	/	/	/		
		**Month 3**	Pain VAS ↓ (cb), BCTQs ↓ (cb), BCTQf ↓ (cb), finger pinch ↑ (cb), LANSS ↓ (cb)<br>CMAPA ↑ (cb)	Pain VAS ↓ (cb), BCTQs ↓ (cb), BCTQf ↓ (cb), finger pinch ↑ (cb, ccD)	Pain VAS ↓ (cb), BCTQs ↓ (cb), BCTQf ↓ (cb), finger pinch ↑ (cb), LANSS ↓ (cb)	Pain VAS ↓ (cb), BCTQs ↓ (cb), BCTQf ↓ (cb), finger pinch ↑	DML, MNCV, DSL, SNAPA, SNCV	LANSS DML, CMAPA, SNCV	DML, CMAPA, MNCV, DSL,	DML, CMAPA, MNCV, SNAPA, SNCV		

			MNCV ↑ (cb), DSL ↓ (cb), SNAPA ↓ (cb)	(cb), LANSS ↓ (cb)<br>DSL ↓ (cb)		SNAPA, SNCV		
Gholipour et al. (Iran, 2023)	II	**-**	**ESWT + LCI (A)**	**Sham ESWT + LCI (B)**	**ESWT + LCI**	**Sham ESWT + LCI**	No	/
		**Month 1**	Pain VAS ↓ (cb), GSS ↓ (cb)	Pain VAS ↓ (cb), GSS ↓ (cb)	/	/		
		**Month 3**	Pain VAS ↓ (cb, ccB), GSS ↓ (cb, ccB)	Pain VAS ↓ (cb), GSS ↓ (cb)	/	/		
		**Month 6**	Pain VAS ↓ (cb, ccB), GSS ↓ (cb, ccB)	/	/	Pain VAS, GSS		
		**After study period**	Surgical intervention = 40% (ccB)	Surgical intervention = 75%	/	/		
Menekseoglu et al. (Turkey, 2023)	II	**-**	**ESWT (A)**	**Sham ESWT (B)**	**ESWT**	**Sham ESWT**	No	/
		**Month 1**	Pain VAS ↓ (cb, ccB), BCTQs ↓ (cb, ccB), BCTQf ↓ (cb, ccB), LANSS ↓ (cb, ccB)<br>DSL ↓ (cb, ccB), SNCV ↑ (cb, ccB), DML ↓ (cb, ccB)	/	SNAPA, CMAPA, MNCV	Pain VAS, BCTQs, BCTQf, LANSS<br>DSL, SNCV, DML, SNAPA, CMAPA, MNCV		
Vongvachvasin et al. (Thailand, 2024)	I	**-**	**ESWT (A)**	**Sham ESWT (B)**	**ESWT**	**Sham ESWT**	No	/
		**Week 3**	BCTQs ↓ (cb, ccB), BCTQf ↓ (cb, ccB)<br>DML ↓ (cb, ccB), DSL ↓ (cb, ccB)	BCTQf ↓ (cb)	SNAPA, CMAPA, CSA	BCTQs<br>SNAPA, CMAPA, DML, CSA		
		**Week 6**	BCTQs ↓ (cb, ccB), BCTQf ↓ (cb, ccB)<br>SNAPA ↑ (cb)	BCTQs ↓ (cb), BCTQf ↓ (cb)	CMAPA, DML, CSA	SNAPA, CMAPA, DML, CSA		
Zong et al. (China, 2023)	IV	**Week 1**	BCTQ ↓ (cb)<br>DSL ↓ (cb), SNCV ↑ (cb), SNAPA ↑ (cb)		DML, MNCV, CMAPA		No	CMAPA was measured different methods and was inconsistent. It improved significantly or not significantly from before treatment depending on the method of measurement.
Bula-Oyola et al. (Spain, 2021)	II	**-**	**ESWT (A)**	**Sham-ESWT (B)**	**ESWT**	**Sham-ESWT**	/	4 studies evaluating ESWT were included.
		**unclear**	Pain VAS ↓ (cb), BCTQs ↓ (cb, ccB), BCTQf ↓ (cb, ccB),<br>SNCV ↑(cb), CMAPA ↑ (cb)	/	Hand-grip strength, pinch strength<br>DSL, DML, SNAPA	Pain VAS, BCTQs, BCTQf, hand-grip strength, pinch strength<br>DSL, DML, SNCV, SNAPA, CMAPA		

Kim et al. (South Korea, 2019)	II	-	**ESWT (A)**	**Sham-ESWT (B)**		**ESWT**	**Sham-ESWT**		/	6 studies evaluating ESWT were included. ESWT = LCI & rESWT = fESWT in all outcome measures
		**Month 2-4**	BCTQs ↓ (cb, ccB), BCTQf ↓ (cb, ccB) DML ↓ (cb, ccB), CMAPL ↓ (cb, ccB), SNCV ↑ (cb, ccB)	/		/	BCTQs, BCTQf DML, CMAPL, SNCV			
Li et al. (China, 2020)	II	**unclear**	**ESWT (A)**	**LCI (B)**		/			No	5 studies evaluating ESWT were included.
			Pain VAS ↓ (cb), BCTQ ↓ (cb), DSL ↓ (cb), SNCV ↑ (cb) CMAPA ↑ (cb, ccB), SNAPA ↑ (cb, ccB)	Pain VAS ↓ (cb), BCTQ ↓ (cb), DSL ↓ (cb), SNCV ↑ (cb) DML ↓ (cb, ccA)						
Xie et al. (China, 2022)	II	-	**ESWT (A)**	**Sham-ESWT (B)**		**ESWT**	**Sham-ESWT**		/	10 studies evaluating ESWT were included. The CSA was only measured in 3 studies.
		**< month 3**	BCTQs ↓ (cb, ccB)	/		Pain VAS, BCTQf SNAPL, SNAPA, CMAPL, CMAPA, SNCV, CSA	Pain VAS, BCTQs, BCTQf SNAPL, SNAPA, CMAPL, CMAPA, SNCV, CSA			
		**> month 3**	Pain VAS ↓ (cb, ccB), BCTQs ↓ (cb, ccB), BCTQf ↓ (cb, ccB) SNAPA ↑ (cb, ccB)	/		SNAPL, CMAPL, CMAPA, SNCV, CSA	Pain VAS, BCTQs, BCTQf SNAPL, SNAPA, CMAPL, CMAPA, SNCV, CSA			
Chen et al. (Taiwan, 2022)	II		**ESWT + Splinting (A)**	**Splinting (B)**		**ESWT + Splinting**	**Splinting**		No	7 studies evaluating ESWT were included. The qDASH score was measured in only one study. Electrodiagnostic studies were conducted only at week 12-16 postt.
		**Week 3-4**	Pain VAS ↓ (cb, ccB), BCTQs ↓ (cb, ccB), BCTQf ↓ (cb, ccB) & qDASH ↓ (cb, ccB)	/		/	Pain VAS, BCTQs, BCTQf, qDASH			
		**Week 8-10**	/	/		Pain VAS, BCTQs, BCTQf, qDASH	Pain VAS, BCTQs, BCTQf, qDASH			
		**Week 12-14**	/	/		Pain VAS, BCTQs, BCTQf, qDASH DML, SNCV	Pain VAS, BCTQs, BCTQf, qDASH DML, SNCV			
Zhang et al (China, 2023)	II	-	**ESWT (A)**	**LCI (B)**	**Sham-ESWT (C)**	**ESWT**	**LCI**	**Sham-ESWT**	No	19 studies evaluating ESWT were included.
		**<1 month**	Pain VAS ↓ (cb, ccC), BCTQ ↓ (cb, ccC)	Pain VAS ↓ (cb, ccC), BCTQ ↓ (cb, ccC)	/	DML	DML, CMAPA	Pain VAS, BCTQ		

			SNCV ↑ (cb, ccC), CSA ↓ (cb, ccC)	SNAPA ↑ (cb), DSL ↓ (cb), CSA ↓ (cb, ccC)				SNCV, DML, CSA		
		**Month 1-6**	Pain VAS↓ (cb, ccB, ccC), BCTQ↓ (cb, ccB, ccC) SNCV ↑ (cb, ccC), DML↓ (cb, ccC), DSL ↓ (cb, ccB), CSA↓ (cb, ccC)	Pain VAS↓ (cb, ccC), BCTQ↓ (cb, ccC) SNAPA ↑ (cb), DML ↓ (cb), DSL ↓ (cb), CSA↓ (cb, ccC)	/	/	CMAPA	Pain VAS, BCTQ SNCV, DML, CSA		
		**>6 months**	Pain VAS ↓ (cb, ccB), BCTQ ↓ (cb, ccB, ccC)	Pain VAS ↓ (cb), BCTQ ↓ (cb, ccC)	/	Pain VAS SNCV, DML, CSA	/	Pain VAS, BCTQ SNCV, DML, CSA		

ESWT = Extracorporeal Shockwave Therapy; LCI = Local Corticosteroid Injection; US = Ultrasonography; LLLT = low-level laser therapy; PRP = Platelet-rich plasma; ALA = Alpha Lipoic Acid; GLA = Gamma Linolenic Acid; PPNL = Polarized Polychromatic Noncoherent Light Therapy; Transcutaneous Electrical Nerve Stimulation Therapy; SWD = Short-Wave Diathermy Therapy; Microwave Diathermy Therapy; PRF = Pulsed Radiofrequency; Pret. baseline = pre-treatment baseline; postt. = post-treatment; VAS = Visual Analog Scale; DASH = Disabilities of the arm, shoulder, and hand score; qDASH = quick DASH; SF-36 = 36-item Short Form Health Survey Questionnaire; R&Ms = Roles and Maudsley score; BCTQs = Boston Carpal Tunnel Questionnaire for symptom severity; BCTQf = Boston Carpal Tunnel Questionnaire for functional status; ADL = Activities of Daily Activity; GSS = Global Symptoms/System Severity; LANSS = Leeds Assessment of Neuropathic Symptoms and Signs; CSA = nerve cross-sectional area; CMAPA = compound muscle action potential amplitude; CMAPL = compound muscle action potential latency; SNAPA = sensory nerve action potential amplitude; SNAPL = sensory nerve action potential latency; DML = distal motor latency; DSL = distal sensory latency; MNCV = motor nerve conduction velocity; SNCV = sensory nerve conduction velocity.
There were no significant differences at baseline between the groups. The following abbreviations and symbols specify in which relation the specific outcome measure is significant:
cb = compared to baseline significantly different; cp = compared to previous value significantly different; ccX = compared to group X (if existent) at the same follow-up; ↑ = significant increase; ↓ = significant decrease.
Example: VAS ↓ (cp, ccX) = The VAS score is significantly lower than the previously recorded VAS score of the same group and is significantly lower than the VAS score of the group X at the same follow-up time. In the column of “No significant changes” are only outcome measures that yielded no significant changes at all in respective group.

Table S16: Carpal Tunnel Syndrome study results

Attachment 2 to: Al-Mousllie I, Vogt PM, Jokuszies A. State of shock – a systematic review of extracorporeal shockwave therapy in hand surgery. GMS Interdiscip Plast Reconstr Surg DGPW. 2025;14:Doc03. DOI: 10.3205/iprs000192
